# Non-reproductive tumor progression impairs ovarian function through CXCL10–IL18R1 axis

**DOI:** 10.1038/s12276-026-01790-4

**Published:** 2026-07-22

**Authors:** Shi-Ya Xie, Shu-Ping Zhang, Cong-Rong Li, Yan Liu, Xian-Zhe Gu, Yan-Jie Yang, Zhi-Xia Yang, Qian Wang, Hong-Jie Yao, Yun-Xia Cao, Zhao-Lian Wei, Enrica Bianchi, Ping Zhou, Cui-Lian Zhang, Xiang-Shun Cui, Feng-Song Wang, Dong Zhang

**Affiliations:** 1 Department of Gynaecology and Obstetrics, The First Affiliated Hospital of Anhui Medical University, Hefei, Anhui China; 2 State Key Lab of Reproductive Medicine and Offspring Health, Nanjing Medical University, Nanjing, Jiangsu China; 3 NHC Key Laboratory of Study on Abnormal Gametes and Reproductive Tract (Anhui Medical University), Hefei, Anhui China; 4 Key Laboratory of Population Health Across Life Cycle (Anhui Medical University), Ministry of Education of the People’s Republic of China, Hefei, Anhui China; 5 Hefei Cancer Hospital, Chinese Academy of Sciences, Hefei, Anhui China; 6 Reproductive Medical Center, Henan Provincial People’s Hospital, People’s Hospital of Zhengzhou University, Zhengzhou, Henan China; 7 Guangzhou National Laboratory, University of Chinese Academy of Sciences, Guangzhou, Guangdong China; 8 Department of Biomedicine and Prevention, University of Rome tor vergata, Rome, Italy; 9 Department of Animal Science, Chungbuk National University, Cheongju, Chungbuk South Korea; 10 College of Life Sciences, Anhui Medical University, Hefei, Anhui China

**Keywords:** Experimental models of disease, Infertility

## Abstract

The ovary is susceptible to harmful environmental factors. Tumor progression (TP) is an intracorporal harmful environmental factor. It is known that TP could impair adjacent tissues through pro-inflammatory cytokines. However, it is unclear whether and how non-reproductive tumor progression (NRTP) impairs ovarian function. In this study, we used MCA205 (mouse fibrosarcoma) cell-allotransplanted B6 mice as a basic model (M group); meanwhile, we established two popular therapeutic models — PD-1 monoclonal antibody injection in allotransplanted mice (PD-1 group) and whole cancer cell vaccine (WCV) injection in allotransplanted mice (WCV group) — to facilitate the discovery process. As expected, TP in M group decreased ovarian function multifacetedly. Interestingly, WCV injection significantly reversed these abnormalities, whereas PD-1 did not. Next, plasma cytokine microarray characterized CXCL10 with both the biggest increment in M group and best rescue in WCV group. Next, we baited the only CXCL10 receptor, IL18R1, within ovaries. Next, we found that CXCL10 directly bound to IL18R1 to impair ovarian function through three pathways: inducing ovarian fibrosis through p-JNK→p-JUN → COL1A1, promoting primordial follicle overactivation through p-AKT→p-FOXO3A, and increasing ovarian inflammation through p-P65 → IFNγ. We have also preliminarily verified the CXCL10–IL18R1 axis and the upper three downstream pathways in MC38 (mouse colon cancer cells)-allotransplanted and B16F10 (mouse melanoma cells)-allotransplanted B6 mice. Finally, we rescued the decreased ovarian function in the M group by blocking the CXCL10 → IL18R1 pathway with CXCL10 antibody or a CXCL10–IL18R1 interface peptide, CIBB. This study provides mechanical evidence and translational strategies of how NRTP impairs ovarian functions and how to target-protect ovaries under NRTP.

## CXCL10–IL18R1 pathway disrupts ovarian health

This study explores the impact of non-reproductive tumor progression on ovarian function, focussing on the role of the cytokine CXCL10. Using a mouse model of fibrosarcoma, researchers identified CXCL10 as a key factor in ovarian damage, acting through the IL18R1 receptor to trigger harmful pathways. The study used various methods, including RNA sequencing and cytokine assays, to uncover the mechanisms by which CXCL10 induces fibrosis, inflammation, and follicle depletion in ovaries. Notably, treatments targeting the CXCL10–IL18R1 interaction, such as CXCL10 antibodies and a designed peptide (CIBB), effectively mitigated ovarian damage. These findings highlight CXCL10 as a potential marker for early ovarian damage in non-reproductive tumor progression and suggest targeted therapies to protect ovarian function. Future research could explore the broader applicability of these findings across different tumor types and refine therapeutic strategies for clinical use.

This summary was initially drafted using artificial intelligence, then revised and fact-checked by the author.

## Introduction

The ovary is sensitive to harmful environmental factors (hEFs)^1,2^. hEFs can be categorized into extracorporeal hEFs and intracorporal hEFs. Non-reproductive tumor progression (NRTP) is a type of intracorporal hEFs, and increased levels of pro-inflammatory cytokines were induced in tumor tissues and caused significant damages to adjacent tissues^3–5^. Presumably, these pro-inflammatory cytokines can reach the ovaries through circulation and exert damaging effects on ovaries before they are diagnosed. However, until now, no related clinical studies have provided definite evidence that NRTP itself impairs ovarian function. The reason is that very soon after diagnosis, cancer therapy and fertility preservation become the primary issues^6,7^. Moreover, most clinical cancer therapies, including conventional chemotherapies^8^, radiotherapy^9^, or recent immune therapies^10,11^, have also been reported to undermine female fertility in various ways, which further distracts researchers’ attention on NRTP-induced ovarian damage.

Considering that the impact of NRTP itself on ovarian function and related mechanisms is difficult to address in clinical females, investigations in a mouse model are necessary. Using a mouse model, we identified the key factor through which NRTP damages ovaries and then designed targeted therapies to protect ovaries in a mouse model, thereby providing novel strategies for the future clinical application of targeted protection of ovaries from NRTP-induced damage. However, no such studies have been conducted in a female mouse model. Notably, only one study reported that acute myeloid leukemia invasion could impair multiple aspects of male fertility, including sperm parameters, spontaneous acrosome reactions, and fertility capacity^12^.

In this study, we created a mouse model of fibrosarcoma, which has no reported relationship with ovaries, in B6 mice (M group) to identify the key NRTP-induced factor that impairs the ovaries and investigate the detailed functional mechanism involved. We also established two popular therapeutic models — PD-1 monoclonal antibody (mAb) injection and whole cancer cell vaccine (WCV) injection — in fibrosarcoma-allografted mice (PD-1 group and WCV group) in parallel to facilitate the discovery process. We found that CXCL10 is the key NRTP-induced cytokine and that IL18R1 is the major ovarian receptor that transduces deleterious downstream signals into ovaries. On the basis of these mechanistic findings, we also developed targeted therapies that specifically prevent CXCL10–IL18R1 binding and thereby protect ovaries from NRTP-induced damage.

## Materials and methods

### Antibodies

Information on all commercial primary and secondary antibodies is included in Supplementary Tables 1.1 and 1.2, respectively.

Rabbit anti-CXCL10 was produced against CLNPESKTIKNL by ZoonBio BioTech (Nanjing, China) and was purified by affinity purification.

### Mouse model

All experimental procedures involving animals were approved by the Institutional Animal Care and Use Committee (IACUC) of Nanjing Medical University (Approval No. IACUC-2205056) and Anhui Medical University (Approval No. LLSC-20232253). All mice were housed under standard specific pathogen-free (SPF) conditions at the animal core facility (ACF). To acquire the ovaries and other tissues, the mice were anesthetized with CO_2_ and then sacrificed by cervical dislocation.

Three mouse models were created as follows:

1. **Mouse tumor cell-allograft NRTP model, WCV, and PD-1 mAb immunotherapy models in B6BL mice**
  We employed four mouse tumor cell lines: MCA205, mouse fibrosarcoma cells; MC38, mouse colon cancer cells; B16F10, mouse melanoma cells; ID8, mouse ovarian epithelial cancer cells. Grouping for these four cell lines is the same; therefore, we used MCA205 cells as an example.
  The mouse model was established as described previously^13^. Four groups were established: the CTR group, the M (MCA205) group, the VM (WCV + MCA205) group, and the PM (anti-PD-1 antibody + MCA205) group.
  For the CTR group, female C57BL/6 mice aged 7–8 weeks were injected with 100 μl of phosphate-buffered saline (PBS) s.c. (subcutaneously) once daily. For the M group, female C57BL/6 mice aged 7–8 weeks received s.c. injections of 1 × 10^6^ MCA205 cells, a malignant cell line derived from mouse fibrosarcoma. Tumor diameters were measured with digital calipers at multiple time points (days 3, 5, 7, 9, and 11), and the tumor area (in mm^2^) was calculated with the following formula: area = 3.14 × width × length/4. For the VM group, on day 12, tumor cells were pretreated with 2 μM MTA (mitoxantrone) for 16 h (WCV), washed with PBS, and injected s.c. (1 × 10^6^ WCV cells in 100 μl of PBS) into naive C57BL/6 mice; then, on day 0, tumor cells were injected as in the M group. For the PM group, on day 0, the tumor cells were injected as in the M group; then, on days 5 (when the tumor size reached 25–45 mm^2^), 8, and 11 (three injections in total for one model), the mice were injected i.v. (intravascularly) with 10 mg/kg anti-PD-1 mAb (in 100 μl of PBS, clone RMP1-14, BioXCell, Lebanon, NH, USA).
2. **CXCL10 antibody injection in tumor-bearing mice**
  Three groups were established: the CTR group, the M (MCA205) group, and the CM (anti-CXCL10 antibody + MCA205) group.
  For the CTR or M group, the treatments were as described earlier. For the CM group, on day 0, tumor cells were injected as mentioned earlier; then, on days 5 (when the tumor size reached 25–45 mm^2^), 8, and 11 (three injections in total for one model), the mice were injected i.v. with 10 mg/kg anti-CXCL10 antibody (in 100 μl of PBS).
3. **Competitive peptide injection in tumor-bearing mice**
  For this, three groups were set up: the CTR group, the M (MCA205) group, and the CI-M (CIBB + MCA205) group.
  For the CTR group, the treatments were as described earlier. For the M group, tumor cells were injected as described earlier, plus 6 mg/kg control cell-penetrating peptide sequence TAT (*trans*-acting activator of transcription of HIV, CYGRKKRRQRRR) injected daily (in 100 μl of PBS). For the CI-M group, on day 0, tumor cells were injected as mentioned earlier; then, beginning the next day, the mice were injected i.p. (intraperitoneally) with 6 mg/kg CIBB peptide daily (in 100 μl of PBS).

For the CIBB design, which inhibits the interaction between CXCL10 and IL18R1, the protein structure of CXCL10 interacting with IL18R1 was predicted by AlphaFold. The sequences in CXCL10 that bound to IL18R1 in the top two prediction models were the M1 sequence (CLNPESKTI) and the M2 sequence (IGKLEIIPASLSCPRVEIIATMK). The Flag sequence (DYKDDDDK) and TAT sequence were fused with the M1 or M2 sequence and synthesized by Shanghai Bootech Bioscience & Technology (Shanghai, China). The combination of M1 and M2 was named CIBB (CXCL10–IL18R1 binding blocker), which was dissolved in 10% dimethyl sulfoxide (Sigma, St Louis, MO, USA) to a stock concentration of 5 mg/ml. The stock peptide was diluted with PBS to a final concentration of 0.5 mg/ml, and the injection dosage was 6 mg/kg.

### Ovary bulk RNA sequencing and analysis

RNA samples were obtained from the ovaries of the mice. Isolating RNA, high-throughput sequencing, and data analysis were done by Seq Health Technology (Wuhan, China), following standard protocols. The library products were then sequenced on a DNBSEQ-T7 sequencer (MGI Tech, Shenzhen, China) with the PE150 model. The original sequence datasets were submitted to the NCBI Sequence Read Archive database and assigned an accession number. Specifically, for the ovary bulk RNA-seq data, the accession number was GSE248829. Genes whose absolute value of the log2 (treated/control) ratio was greater than or equal to 1.2 and whose *q*-value was less than 0.001 were considered differentially expressed genes (DEGs).

### Multiplex immunoassay

Multiplex immunoassays were performed by Shanghai Universal Biotech (Shanghai, China). Blood plasma was collected from the CTR group, the M group, the VM group, and the PM group (five replicates per group). An aliquot of 50 μl of the collected sample was subjected to cytokine measurement via Luminex magnetic beads according to the Mouse LX-MultiDTM-31 protocol (Bio-Rad, Hercules, CA, USA; Cat. No. 12009159). The information was gathered with a Bio-Plex 200 system (Bio-Rad) with high-throughput fluidics and analyzed via Bio-Plex Manager software version 6.1 (Bio-Rad).

### Flow cytometric analysis

Six mouse ovaries were used for each sample. The freshly dissected ovaries were immediately placed in ice-cold PBS, carefully dissected, and minced with scissors. Next, the ovaries were digested with 3 ml of digestion buffer containing RPMI-1640 medium, 4 mg of collagenase IV (Sigma; Cat. No. AC5138), and 0.25 mg of deoxyribonuclease I (Sigma; Cat. No. DN25) for 40 min on a shaker at 120 rpm and 37 °C. Next, the digestion was stopped by the addition of 1 ml of PBS containing 20% FBS (fetal bovine serum).

The cell pellets were centrifuged for 10 min at 600×*g* and 4 °C, resuspended in fluorescence-activated cell sorting (FACS) buffer (PBS with 0.5% bovine serum albumin (BSA) and 5 mM EDTA), and filtered through a 70 μm nylon mesh filter (Biosharp, Beijing, China; Cat. No. BS-70-CS).

The cells were stained for live & dead viability via a Zombie Violet™ Fixable Viability Kit (BioLegend, Beijing, China; Cat. No. 423113) for 20 min, followed by incubation with an anti-CD16/CD32 mAb (BioLegend; Cat. No. 156603) to block nonspecific antibody staining. The cells were incubated with cell surface antigen-specific antibodies, such as an anti-CD45 antibody (BioLegend; Cat. No. 103116), an anti-F4/80 antibody (BioLegend; Cat. No. 123110), an anti-CD11b antibody (BioLegend; Cat. No. 101206), and an anti-CD86 antibody (BioLegend; Cat. No. 105014), on ice for 60 min. After washing, ~100 μl of residual volume was retained, and the cells were fixed with 100 μl of IC fixation solution (Thermo Fisher, Waltham, MA, USA; Cat. No. 88-8824-00) for 60 min. The cells were washed twice with 2 ml of permeabilization solution and then incubated with an anti-CD206 antibody (BioLegend; Cat. No. 141712) for 60 min. Finally, the cells were resuspended in 500 μl of PBS and passed through a 70-μm nylon mesh filter for analysis via a Cytoflex LX cell analyzer (Beckman, Miami, FL, USA) through CytExpert software.

After being gated, CD45^+^, F4/80^+^, and CD11b^+^CD86^+^/CD206^−^ cells were analyzed as M1 macrophages, whereas CD86^−^/CD206^+^ cells were analyzed as M2 macrophages. The gating strategy is shown in Supplementary Fig. 5.

### Expression and purification of IL18R1 in SF9 cells

The IL18R1 protein, which was fused with EGFP-Strep II, was cloned and expressed through a Bac-to-Bac system (Thermo Fisher). In brief, the corresponding sequence (Supplementary Table 2) was cloned and inserted into pFastBacHTA (Supplementary Table 4) and then transformed into DH10Bac (Vazyme, Nanjing, China)-competent *Escherichia* *coli*. The bacmid was isolated from *E. coli* via the QIAFILTER plasmid purification method (QIAGEN, Tegelen, The Netherlands) and then transfected into Sf9 cells (Genetime ExCell Technology, Shanghai, China; Cat. No. ATCC CRL-3357) with Cellfectin II Transfection reagent (Thermo Fisher) to produce first-round baculovirus. Fresh Sf9 cells were infected with the first-round baculovirus for 48 h for second-round and third-round virus amplification. An aliquot of 20 μl of the third-round virus mixture was used to infect 500 ml (SFM900-II medium with 5% FBS, Thermo Fisher) of Sf9 cells (1.5 × 10^6^/ml) for protein expression in an orbital shaker (Shanghai Zhichu Instrument) at 27 °C and 200 rpm. The infected cells were subsequently resuspended in lysis buffer (containing 50 mM Tris, 10% sucrose, 50 µM ATP, 1 mM phenylmethylsulfonyl fluoride (PMSF), 5 mM dithiothreitol (DTT), 1% NP40, 10 mM imidazole, 1× protease inhibitor and phosphatase inhibitor, pH 7.0, with HCl). The cell lysate was further lysed with a high-pressure cell disrupter (Union Biotech, Shanghai, China) and centrifuged; the lysate supernatant was incubated with 1 ml of Ni-NTA Superflow resin (QIAGEN) for 1 h at 4 °C. The resin was then transferred into a 5 ml chromatography column (Biocomma, Shenzhen, China) and washed with four column volumes of wash buffer (40 mM imidazole, free of PMSF). Finally, the protein was eluted with resuspension buffer (500 mM imidazole without PMSF). The eluted protein was concentrated by a size-exclusion spin column and exchanged into BRB80 buffer (80 mM HEPES, 1 mM MgCl_2_, and 1 mM EGTA, pH 6.8 by KOH) with 10% glycerol, 50 μM ATP and 5 mM DTT. The protein was aliquoted and stored at −80 °C for future use. The concentration of IL18R1 was determined by comparing its intensity with that of 0.4 mg/ml BSA.

### Expression and purification of CXCL10 in *E. coli*, GST pull-down assay, and mass spectrometry

The construct containing pGEX-6P1-CXCL10-StrepII was propagated in BL-21 *E. coli* (Vazyme; Cat. No. C504). An aliquot of 10 ml of overnight culture was added to 1000 ml of Luria–Bertani medium, and the *E. coli* was grown on an orbital shaker at 27 °C and 200 rpm until the OD_600_ reached 0.6. Then, the *E. coli* culture was induced with 0.1 mM IPTG (isopropyl-β-D-thiogalactoside, Yeasen, Shanghai, China; Cat. No. 10902ES10) at 16 °C for 16 h. Next, the *E. coli* culture was collected by centrifugation for 10 min at 3500 rpm and 4 °C, washed with ice-cold PBS at pH 7.3, and lysed in 25 ml of lysis buffer (PBS, pH 7.3; 1 mM DTT (Amresco, Framingham, MA, USA; Cat. No. M109); 1 mM PMSF (Amresco; Cat. No. M145); 1:100 InStab™ protease inhibitor cocktail (Yeasen; Cat. No. 20124ES10); 1:100 InStab™ phosphatase inhibitor cocktail (Yeasen; Cat. No. 20109ES20); and 1% Triton X-100). After standing on ice for 15 min, the *E. coli* was broken through a high-pressure crusher, and the supernatant was collected by centrifugation at 4 °C. The fusion protein was captured with 600 μl of glutathione agarose resin (BBI Life Science, Shanghai, China; Cat. No. C600031-0010) at 4 °C for 30 min and then collected by low-speed centrifugation. To purify the fusion protein, the GST beads were washed with 20 ml of washing buffer three times. The concentration of CXCL10 was determined by comparing its intensity with that of 0.4 mg/ml BSA.

To identify CXCL10-interacting proteins in the ovaries, the ovarian tissue of the mice was lysed in lysis buffer (Yeasen), and the ovarian lysate was incubated with CXCL10-bound glutathione agarose beads at 4 °C for 4 h. The beads were washed with lysis buffer and washing buffer, and the antibody immunocomplex was subsequently sent to Biotech-Pack (Beijing, China) for mass spectrometry analysis of CXCL10-interacting proteins. The raw data generated by mass spectrometry were submitted to the ProteomeXchange Consortium (http://proteomecentral.proteomexchange.org) through the iProX partner repository and can be accessed via the dataset identifier PXD047305.

### Cell culture, plasmid transfection, and co-immunoprecipitation (co-IP)

Mouse fibrosarcoma cells (MCA205) were obtained from the National Cancer Institute and sold by Shanghai Yu Bo Biotech (Shanghai, China; Cat. No. YB867). The cells were confirmed to be free of mycoplasma contamination and were routinely maintained in a culture medium supplemented with MycAway™ mycoplasma elimination reagent (Yeasen; Cat. No. 40607ES08). The cells were cultured in RPMI-1640 with 10% FBS. MCA205 cells were transfected with pcDNA3.1(+)-IL18R1-EGFP-StrepII and pcDNA3.1(+)-CXCL10-TagRFP-Flag for IP. A total of 2 × 10^6^ cells were lysed in 250 μl of IP buffer, and protein A/G beads (Yeasen) were preincubated at 4 °C for 4 h to prevent nonspecific binding. Next, 3 μg of mouse anti-Strep II-Tag mAb or rabbit anti-DDDDK-tag (Flag) mAb, along with rabbit or mouse control IgG, was coupled to the protein A/G beads in 250 μl of IP buffer at 40 °C for 4 h on a rotating wheel. The protein A/G-coupled control IgG or specific antibodies were subsequently incubated with precleaned MCA205 cell lysates at 4 °C overnight. Finally, after being washed three times in IP buffer, protein blot analysis was conducted on the immunocomplexes bound to the anti-Strep II-Tag mAb or rabbit anti-DDDDK-tag (Flag) mAb.

### Ovarian hematoxylin–eosin staining and follicle counting

To minimize the systematic errors caused by the estrus cycle, for all oocyte-related experiments and follicle counting, we used pregnant mare serum gonadotropin (PMSG) (5 IU per mouse; Ningbo Second Hormone Factory, Ningbo, China) to synchronize the cycle and promote follicle maturation. About 48 h after PMSG injection, the mice were sacrificed, and the ovaries were harvested; one ovary was frozen in liquid nitrogen for protein sample preparation, and the other was washed with 0.9% NaCl solution, fixed with 4% paraformaldehyde (PFA) overnight, embedded in paraffin, and sectioned continuously at a thickness of 5 µm. The sections were deparaffinized in xylene, rehydrated gradually in high-to-low-concentration ethanol and distilled water, stained with hematoxylin and eosin, dehydrated in ethanol, cleared in xylene, and mounted with neutral resin (Solarbio, Beijing, China) for follicle counting under a microscope (Nikon Eclipse SI, Japan).

Every other section was counted because the distance between every three sections is approximately the size of an oocyte nucleus, and only visible and clearly identifiable follicles were included in the count. The follicle stages were classified according to the Pedersen criteria^14,15^. Primordial or primary follicles were surrounded by a single layer of flattened or cuboidal granulosa cells, respectively. Follicles that had multiple layers of cuboidal granulosa cells surrounding the oocyte were defined as secondary follicles, whereas follicles that had a clear cavity containing follicular fluid were defined as AFs.

### Masson staining

Mouse ovaries were collected and washed with 0.9% NaCl solution, soaked in 4% PFA, and fixed overnight on a rotary shaker. The ovaries were embedded in paraffin and cut into 5-µm-thick sections. The sections were stained with Masson’s trichrome staining solution, according to the manufacturer’s protocol (SbjBio, Nanjing, China). In brief, the sections were dewaxed and incubated in Bouin’s solution overnight at room temperature and then stained with aniline blue and Scarlet to visualize the collagen fibers and nuclei, respectively, under dark conditions at 4 °C. After differentiation with Azure A, phosphomolybdic acid, and Fast Green, the sections were dehydrated, mounted, and observed under a microscope (Nikon Eclipse SI) after weak acid treatment.

### Immunofluorescence and immunohistochemistry in paraffin sections

The paraffin sections were dewaxed, hydrated, and then boiled in citrate buffer for antigen retrieval. For immunohistochemistry, the sections were incubated in 3% hydrogen peroxide at room temperature for 15 min. The sections were circled with an immunohistochemical pen. After being blocked with goat serum for 1 h, the paraffin sections were incubated with the primary antibody overnight at 4 °C. For immunofluorescence, the next day, the secondary antibody and 4’,6-diamino-2-phenylpyridine (DAPI) nuclear stain were applied to the paraffin sections, which were then mounted with an anti-quenching solution. For immunohistochemistry, a rabbit two-step detection kit (ZSGB-BIO, Beijing, China; Cat. No. PV-9001) and a diaminobenzidine (DAB) chromogenic kit (ZSGB-BIO; Cat. No. ZLI-9017) were used, according to the manufacturer’s instructions. The paraffin sections were counterstained with hematoxylin, dehydrated, cleared, and mounted with neutral resin (Absin Biotech, Shanghai, China; Cat. No. abs9177). Fluorescence imaging was performed under a confocal microscope (Zeiss LSM 810, Germany or Nikon AXR, Japan), and immunohistochemistry imaging was performed under a microscope (Nikon Eclipse SI).

### Oocyte collection and in vitro maturation

For ovulated oocytes, the mice were first injected with 5 IU PMSG i.p.; 48 h later, the mice were injected with 5 IU HCG (human chorionic gonadotropin, Ningbo Second Hormone Factory) to induce ovulation. Twelve hours later, the mice were sacrificed, and the ampulla of the fallopian tube was torn open with two needles. The ovulated COCs (cumulus–oocyte complexes) with mature MII (metaphase II) oocytes were released into the HEPES buffer.

For fully grown germinal vesicle oocytes, the ovaries were harvested as described earlier, large antral follicles were punched with a syringe needle in HEPES buffer, and the COCs were released. Nude oocytes were separated from the COCs by repeated blowing-and-suction with a mouth glass pipette (~160 μm in diameter) in MEM (minimum essential medium) + medium (0.01 mM EDTA, 0.23 mM Na-pyruvate, 0.2 mM penicillin/streptomycin, and 3 mg/ml BSA in MEM; Thermo Fisher). Every 50 oocytes were cultured in 100 μl mini-drops of MEM+ containing 20% FBS covered with mineral oil in an incubator at 37.0 °C, 5% O_2_, and 5% CO_2_ in a humidified atmosphere. Before the experimental treatment, 2.5 μM milrinone was added to all media to inhibit the onset of meiosis.

### Immunofluorescence staining of oocytes

After a brief rinse with PBS/0.05% polyvinylpyrrolidone (to prevent adhesion), the oocytes were permeabilized with 0.5% Triton X-100/PHEM for 5 min, fixed in 3.7% PFA/PHEM (18.14 g PIPES, 6.5 g HEPES, 3.8 g EGTA, 0.99 g MgSO4, pH 7.0 w/KOH) for 20 min, and washed three times (10 min each) with PBS/polyvinylpyrrolidone. The oocytes were then incubated at room temperature in blocking buffer (1% BSA/PHEM, 100 mM glycine), followed by overnight incubation at 4 °C with the primary antibody. After being washed three times (10 min each) with PBST (PBS containing 0.05% Tween-20), the oocytes were incubated with a fluorescently labeled secondary antibody at room temperature for 45 min. The oocytes were stained with DAPI for 15 min, washed three times with PBST, and mounted on a glass slide with double-sided tape to secure the edges of the coverslip. Excess water was removed, an anti-quenching agent was added, and the coverslip was sealed with colorless nail polish around the edges. Imaging was performed under a confocal microscope (Zeiss LSM 810, Germany or Nikon AXR).

### *Cxcl10*-KO mice

*Cxcl10* global knockout (KO) mice were bought from Shanghai Model Organisms Center, Inc. (Shanghai, China; Cat. No. NM-KO-190066). The mouse was generated by CRISPR–Cas9 technology to remove exons 2–4, resulting in frameshift and protein reading frame premature termination, the genotyping primers are: forward primer, 5ʹ-TGCTGCCGTCATTTTCTGCCTCAT-3ʹ; reverse primer, 5ʹ-GCCTTTTCCCTTTTTGCCTCACCA-3ʹ. The genotyping PCR program with 2× Magic Green Tag SuperMix greenmix (TOLOBIO, Shanghai, China; Cat. No. 21502-04) was as follows: 94 °C for 5 min, 35 cycles of melting at 95 °C for 30 s, annealing at 63 °C for 30 s, and extension at 72 °C for 10 s, with final extension at 72 °C for 5 min in the end.

### Single-cell RNA sequencing and data analysis

Single-cell suspensions of 1 × 10^5^ cells/ml in PBS were prepared. Single-cell suspensions were then loaded onto microfluidic devices, and single-cell RNA sequencing (scRNA-seq) libraries were constructed according to the Singleron GEXSCOPE protocol via the GEXSCOPE Single-Cell RNA Library Kit (Singleron), which included cell disruption, mRNA trapping, cell labeling (with barcodes) and mRNA labeling (with unique molecular identifier (UMI)), reverse transcription of mRNA into complementary DNA (cDNA) and amplification, cDNA fragmentation, and finally cDNA fragment cloning. Individual libraries were diluted to 4 nM and pooled for sequencing. Pools were sequenced on a NovaSeq 6000 (Illumina, San Diego, CA, USA) with 150 bp paired-end reads. The raw reads were processed with fastQC and fastp to remove low-quality reads. Poly-A tails and adaptor sequences were removed via Cutadapt. After quality control, the reads were mapped to the reference genome GRCm38 (Ensembl version 99 annotation) via STAR. Gene counts and UMI counts were acquired via featureCounts software. Expression matrix files for subsequent analyses were generated on the basis of gene counts and UMI counts. We selected the genes expressed in more than 10% of the cells in either of the compared groups of cells and with an average log (fold change) value greater than 1 as DEGs. The adjusted *P*-value was calculated via Benjamini–Hochberg correction, and a value of 0.05 was used as the criterion to evaluate the statistical significance. For cluster DEGs inclusion, the threshold values of log2(M/CTR), log2(VM/M), and log2(PM/VM) are ≥0.25 or ≤ −0.25.

### In vitro culture and treatment of mouse ovaries

The ovarian culture medium used was α-MEM (Gibco; Cat. No. C12571500BT) supplemented with 3 mg/ml BSA, 0.23 mM pyruvic acid, 50 μg/ml vitamin C, 0.03 U/ml FSH (follicle-stimulating hormone, Sigma; Cat. No. F4021), 75 mg/l penicillin, and 50 mg/l streptomycin. Gelatin sponges of appropriate size were cut and placed in 24-well culture plates with ~100 μl of culture medium. Mouse ovaries were obtained as described earlier, separated, cut into small pieces, and then placed on gelatin sponges. For CXCL10 or CXCL10 + CXCL10 antibody treatment, the final concentrations of the purified recombinant CXCL10 and CXCL10 antibody were 1 μg/ml and 10 μg/ml, respectively; for CXCL10 or CXCL10 + IL18R1 antibody treatment, the final concentrations of the purified recombinant CXCL10 and IL18R1 antibody were 1 μg/ml and 10 μg/ml, respectively.

### Animal and ovary grouping, sample picking, sample size estimation, and animal and ovary inclusion/exclusion

For grouping, sample picking, and sample size estimation of the mouse experiments, under the prerequisite of the 3R principle, we employed enough mice to ensure that the results were credible. Ten mice per group were used for each batch of experiments (additional batches under the same experimental conditions were used depending on the statistical results of the previous batch). Mouse grouping before the start of the experiments and mouse picking for data collection at the end were determined with an online random number generator (https://www.jyshare.com/front-end/6680/). Sample size estimation mainly depends on statistics. Unpicked spare samples (ovaries and plasma) were stored at −80 °C. Data collection, data analysis, and statistics will end if the data are normally distributed and if the statistics reach *P* ≤ 0.05, and at this point, the sample size will be fixed; if the statistics do not reach a significant level, more samples from the unpicked spare samples will be chosen randomly for additional data collection and analysis until the final statistics reach *P* ≤ 0.05. By contrast, for some indices, if there seemed to be no significant trend between groups with three repeats, we determined that there were no significant differences between groups and that the sample size would not increase. For other experiments (for example, experiments using in vitro-cultured ovaries), the principles are the same as mentioned earlier.

For mouse inclusion/exclusion, before the start, the enrolled mice were approximately the same age (7–8 weeks) and were expected to be of similar weight under standardized SPF housing conditions; therefore, mice with significantly greater weights (20% above the average weight of all enrolled mice) were excluded. Any mice with obvious unexpected sickness (slow movement, messy hair, and a glassy eye) were excluded. Considering the potential impacts of stress on various aspects of mouse physiology, any mouse subjected to acute stress (sudden heavy drop owing to operational error, and injury owing to injection error) was also excluded. All properly treated mice will be included.

For ovary inclusion/exclusion, as we always synchronize the estrus cycle before the start, the ovary weight within the same group should be close to the average weight; any ovary with a significantly greater weight (20% above the average weight of all enrolled ovaries) will be excluded. All properly treated ovaries will be included.

### Sample inclusion/exclusion, cell and region selection, image acquisition, measurement, and data entry

Any sample partially destroyed by unexpected operational errors (for example, immunostaining slides exposed to strong mercury lamp light, ovaries with overdrying, frozen blot samples falling into a warm water bath, immunostaining and immunohistochemistry cell and tissue samples disrupted by accidental coverslip displacement) will be excluded; all randomly picked properly made samples will be included.

Cell and region picking and raw image acquisition were performed in a blinded manner. In detail, the first author only assigned numbers to a finished sample (for example, an immunohistochemistry slide) without actual group info on it (named groups 1, 2, and 3); the first author documented the actual group and slide information in the Excel file. One of the co-first authors randomly picks the cell/region and then performs image acquisition (image taking), and enough raw images are acquired and exported in .tif format and saved in the raw image folder. Then, the same co-first author or another co-first author randomly (in the same way as above) picked the proper amount (depending on the first round of statistics) of the tif image for image measurement and data entry. The first author acted as a group leader and assigned the tasks and then coordinated the data measured by the co-first author with the documented group and slide information. The signal intensity in the original tif images (blot, DNA gel, and immunofluorescence image) was measured in ImageJ.

### Statistical analysis

All the statistical graphs showing the western blots and DNA gels are based on three to six independent repetitions, and follicle counting was based on five independent repetitions. The statistical analysis of the immunofluorescence images, Masson’s trichrome staining, and immunohistochemistry results were based on three independent repetitions. Each dot on the graphs represents one data point. The normal distribution of the data points was examined in GraphPad (Prism, San Diego, CA, USA) before statistical analysis. Detailed information about the number of repeats and the number of data points is provided in the figure legends. When the standard error of all individual data points in a group collected randomly was significantly smaller than the average value, the corresponding sample size was deemed appropriate and reliable. The data are presented as the mean ± standard error of the mean (SEM). For statistical comparisons between two groups, we employed an unpaired two-tailed *t*test in GraphPad (Prism); for statistical comparisons between three or more groups, we used ANOVA (one-way non-parametric analysis of variance, Prism). Statistical significance was determined at P-values less than 0.05.

## Results

### NRTP significantly impaired ovarian function

At present, no studies have investigated the impact of NRTP itself on ovaries, making it impossible to target and protect against NRTP-induced ovarian damage and decreased fertility before cancer diagnosis and cancer therapy in females. To address this, we established an allograft NRTP tumor model in normal B6 mice with MCA205 cells (M group). This fibrosarcoma cell line is a non-reproductive malignant tumor line with rapid progression and a high death rate; it can form tumors in normal B6 mice in 1–2 weeks, making it an ideal model to study female fertility under various treatments.

**Fig. 1:**
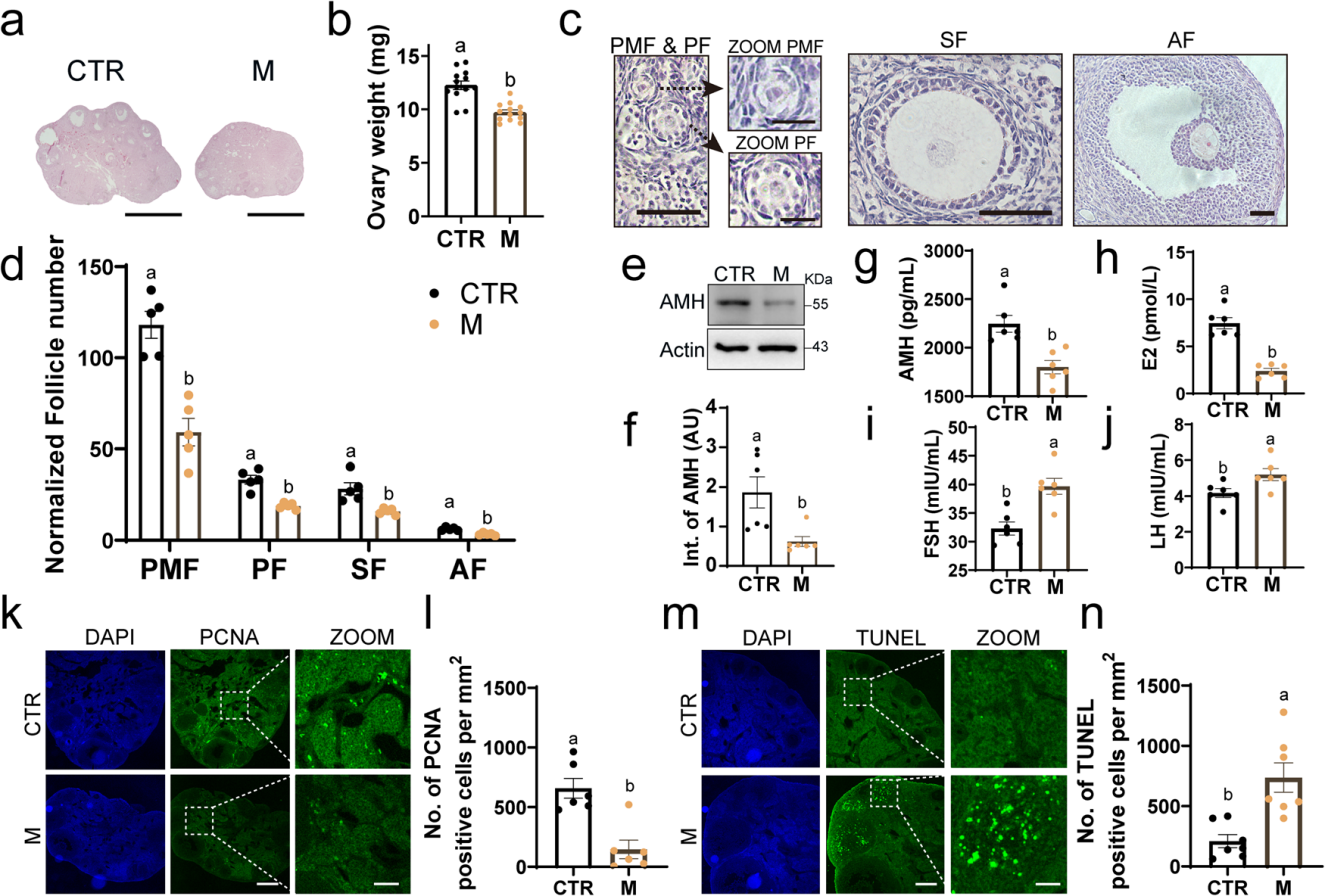
Allotransplanted non-reproductive tumors diminished ovarian function. **a**,**b**, Allotransplanted mouse tumor cells (MCA205) significantly decreased ovary weight; *N* = 13 for both groups; unpaired two-tailed *t*test; *P* < 0.0001. Scale bar in part **a**, 1 mm. **c**, Standard hematoxylin–eosin staining images of primordial follicles (PMFs), primary follicles (PFs), secondary follicles (SFs), and antral follicles (AFs) for follicle counting in part **d**. Scale bar, 50 µm; scale bars in the zoom panels, 20 µm. **d**, The number of follicles (normalized by ovary weight) at each stage significantly decreased in the tumor-bearing B6 mice; *N* = 5 for both groups; for PMF, *P* = 0.0005; for PF, *P* = 0.0004; for SF, *P* = 0.0066; for AF, *P* = 0.0005. **e**,**f**, Western blot analysis and quantification revealed that the levels of the anti-Müllerian hormone (AMH), an indicator of ovarian reserve function, decreased in the ovaries of the tumor-bearing mice; *n* = 6 for both groups; unpaired two-tailed *t*test, *P* = 0.0237. **g**–**j**, Enzyme-linked immunosorbent assay (ELISA) showed the decrease of plasma AMH (G, *N* = 6, *P* = 0.0024) and E2 (H, *N* = 6, *P* < 0.0001) and the increase of follicle-stimulating hormone (FSH) (G, *N* = 6, *P* = 0.0022) and luteinizing hormone (LH) (G, *N* = 6, *P* = 0.0318) in M group. **k–n**, Immunofluorescence staining of ovarian paraffin sections revealed that the signal of the cell proliferation marker proliferating cell nuclear androgen (PCNA) (parts **k** and **l**) decreased (*N* = 6 for both groups; *P* = 0.0011), whereas the terminal deoxynucleotidyl transferase dUTP nick-end labeling (TUNEL) signal (parts **m** and **n**), an apoptosis marker, increased in the ovaries of the tumor-bearing mice (*N* = 7 for both groups; *P* = 0.002). PCNA or TUNEL is shown in green, and DNA is shown in blue. Scale bars in parts **k** and **m**, 100 µm; scale bars in zoom, 25 µm. Unpaired two-tailed *t*test is used for statistical comparison between two groups. Different letters above the columns in the graphs indicate significant differences. For follicle counting, the number of independent biological repeats (R) was 5; for western blot and ELISA, the number of independent biological repeats (R) was the same as technical repeats (*N*); for all other experiments, the number of independent biological repeats (R) was 3. CTR, control; DAPI, 4′,6-diamidino-2-phenylindole.

After approximately day 7 of s.c. injection, MCA205 cells formed observable tumor nodes; these results are similar to those previously reported^13^, indicating that the tumor model was successfully established. Next, we found that in the M group, the size and weight of the ovaries, as well as the number of follicles at each stage, significantly decreased (Fig. 1a–d and Supplementary Fig. 1), indicating that cancer cells caused prominent damage to ovarian function. Moreover, western blot analysis and enzyme-linked immunosorbent assay confirmed a decrease in anti-Müllerian hormone (AMH) levels (Fig. 1e–g) and alteration in E2, FSH, and luteinizing hormone levels (Fig. 1h–j), indicating the ovary dysfunction. Furthermore, in the M group, the health status of the ovarian cells significantly worsened, as indicated by a decreased proliferating cell nuclear androgen (PCNA) signal and an increased TUNEL (terminal deoxynucleotidyl transferase dUTP nick-end labeling) signal (Fig. 1k–n and Supplementary Fig. 15a,b).

**Fig. 2:**
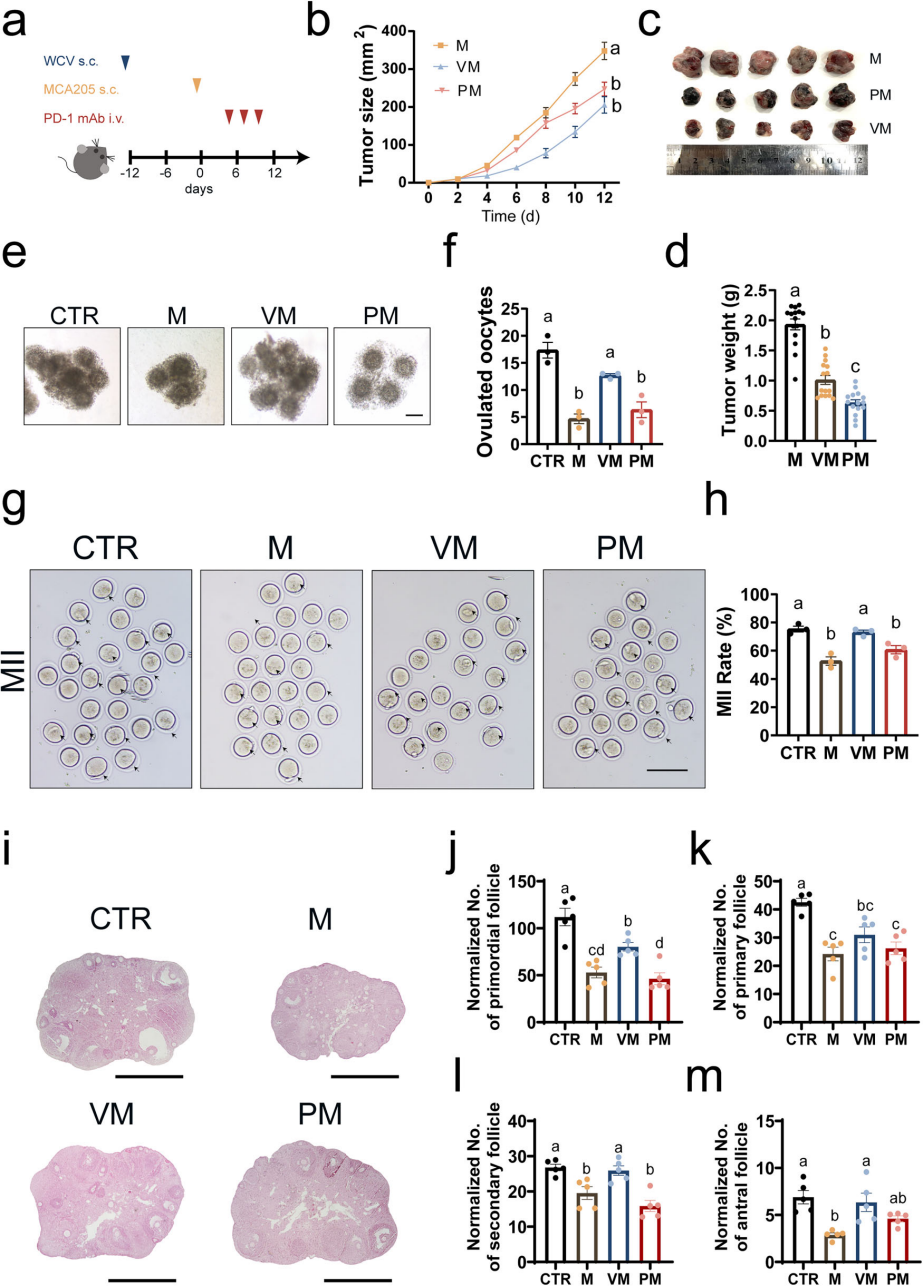
WCV but not anti-PD-1 antibody protected ovarian function. **a**, Diagram showing the schedule for different treatments in the multigroup experiment. The whole cancer cell vaccine (WCV, MCA205 tumor cells treated with MTA) was injected subcutaneously (s.c.) on day 12; MCA205 tumor cells were injected s.c. on day 0; and the anti-PD-1 antibody was injected intravascularly on days 5, 8, and 11. **b**, Four groups were established: the CTR group (control) — normal B6 mice injected with phosphate-buffered saline only; the M (MCA205) group — normal B6 mice injected s.c. with MCA205 cells; the VM (WCV + MCA205) group — normal B6 mice treated with WCV plus MCA205 cells; and the PM (anti-PD-1 antibody + MCA205) group — normal B6 mice treated with anti-PD-1 antibody plus MCA205 cells. Tumor size measurements at multiple time points (days 3, 5, 7, 9, and 11) revealed that tumor growth in the VM and PM groups was significantly slower than that in the M group and that tumor growth in the VM group tended to be even slower than that in the PM group. *N* = 9 for the VM group; *N* = 10 for the M and PM groups. VM versus M, *P* = 0.0002; M versus PM, *P* = 0.0052. **c**, Images of tumors from each group. **d**, Tumor weight measurements at the end of the experiments revealed that the tumor weight in the VM and PM groups was significantly lower than that in the M group and that the tumor size in the VM group tended to be even lower than that in the PM group. *N* = 15 for all groups; M versus VM, *P* < 0.0001; M versus PM, *P* < 0.0001; VM versus PM, *P* = 0.0019. **e**,**f**, Quantification revealed that the number of ovulated oocytes in the M and PM groups was significantly lower than that in the CTR and VM groups; *N* = 3 for all groups; CTR versus M, *P* = 0.0002; CTR versus PM, *P* = 0.0006; M versus VM, *P* = 0.0046; VM versus PM, *P* = 0.0175. **g**,**h**, In vitro maturation and quantification revealed that the metaphase II (MII) rate in the M group was the lowest, whereas no significant differences were observed between the CTR and VM groups, and the MII rate in the VM group tended to be greater than that in the PM group; *N* = 3 for all groups; for CTR versus M, *P* = 0.0008; CTR versus PM, *P* = 0.0117; M versus VM, *P* = 0.0016; VM versus PM, *P* = 0.0293. **i**–**m**, Hematoxylin and eosin staining and follicle counting (normalized by ovary weight) revealed a significant reduction in the number of primordial follicles (PMFs), primary follicles (PFs), secondary follicles (SFs), and antral follicles (AFs) in the M group; WCV treatment significantly reversed the reduction in these numbers in the VM group, whereas anti-PD-1 antibody treatment did not rescue these numbers in the PM group. *N* = 5 for all groups. For PMF, CTR versus M, *P* < 0.0001; CTR versus VM, *P* = 0.0191; CTR versus PM, *P* < 0.0001; M versus VM, *P* = 0.0466; VM versus PM, *P* = 0.0015. For PF, CTR versus M, *P* < 0.0002; CTR versus VM, *P* = 0.0114; CTR versus PM, *P* = 0.0006. For SF, CTR versus M, *P* = 0.0129; CTR versus PM, *P* = 0.0004; M versus VM, *P* = 0.0301; VM versus PM, *P* = 0.0008. For AF, CTR versus M, *P* = 0.0019; M versus VM, *P* = 0.0065. Scale bars in part **e**, 100 µm; scale bars in part **g**, 200 µm; scale bars in part **i**, 1 mm. One-way analysis of variance is used for statistical comparison between multiple (three or more) groups in the graphs, different letters above the columns indicate significant differences. For follicle counting, the number of independent biological repeat (R) was 5; for all other experiments, the number of independent biological repeats (R) was 3. mAb, monoclonal antibody.

Next, we used two popular immunotherapies, anti-PD-1 mAb (PM) therapy and WCV therapy, and investigated whether they worsened or rescued the ovarian damage caused by cancer. In our preliminary experiments, WCV seemed to protect ovary compared with M group, whereas another popular immunotherapy, PD-1, showed no beneficial effect. Therefore, we guessed that a multigroup comparative approach would help narrow the range of candidate key factors responsible for NRTP‑induced ovarian injury. Additionally, it would also be valuable to find out which key factor decides the distinct effects of these two popular immunotherapies on the ovary. We did not include other standard treatments, such as all kinds of chemotherapies and radiotherapies, as these therapies cause random and widespread DNA damage, which might confound the identification of specific key mechanisms. As previously reported, both PD-1 injection and WCV injection significantly reduced MCA205 tumor progression (TP) (Fig. 2a–d); however, there were large differences in their impacts on ovarian performance. The number of ovulated oocytes (mature MII oocytes) in the M group was substantially lower than that in the control group; WCV treatment significantly increased this number in the VM group, whereas PD-1 antibody treatment did not affect the PM group (Fig. 2e,f). Second, the in vitro maturation rate (MII rate) of germinal vesicle oocytes retrieved from ovaries in the M group was significantly lower than that in the control group; WCV treatment significantly improved the MII rate in the VM group, whereas PD-1 antibody treatment did not affect PM group mice (Fig. 2g,h). Third, the numbers of primordial follicles (PMFs) and developing follicles at each stage were significantly lower in the M group than in the control group; WCV treatment increased these numbers in the VM group, whereas PD-1 treatment did not have an obvious rescue effect on the PM group (Fig. 2i–m and Supplementary Fig. 1).

### CXCL10 is the primary factor for impaired ovarian function upon NRTP

**Fig. 3:**
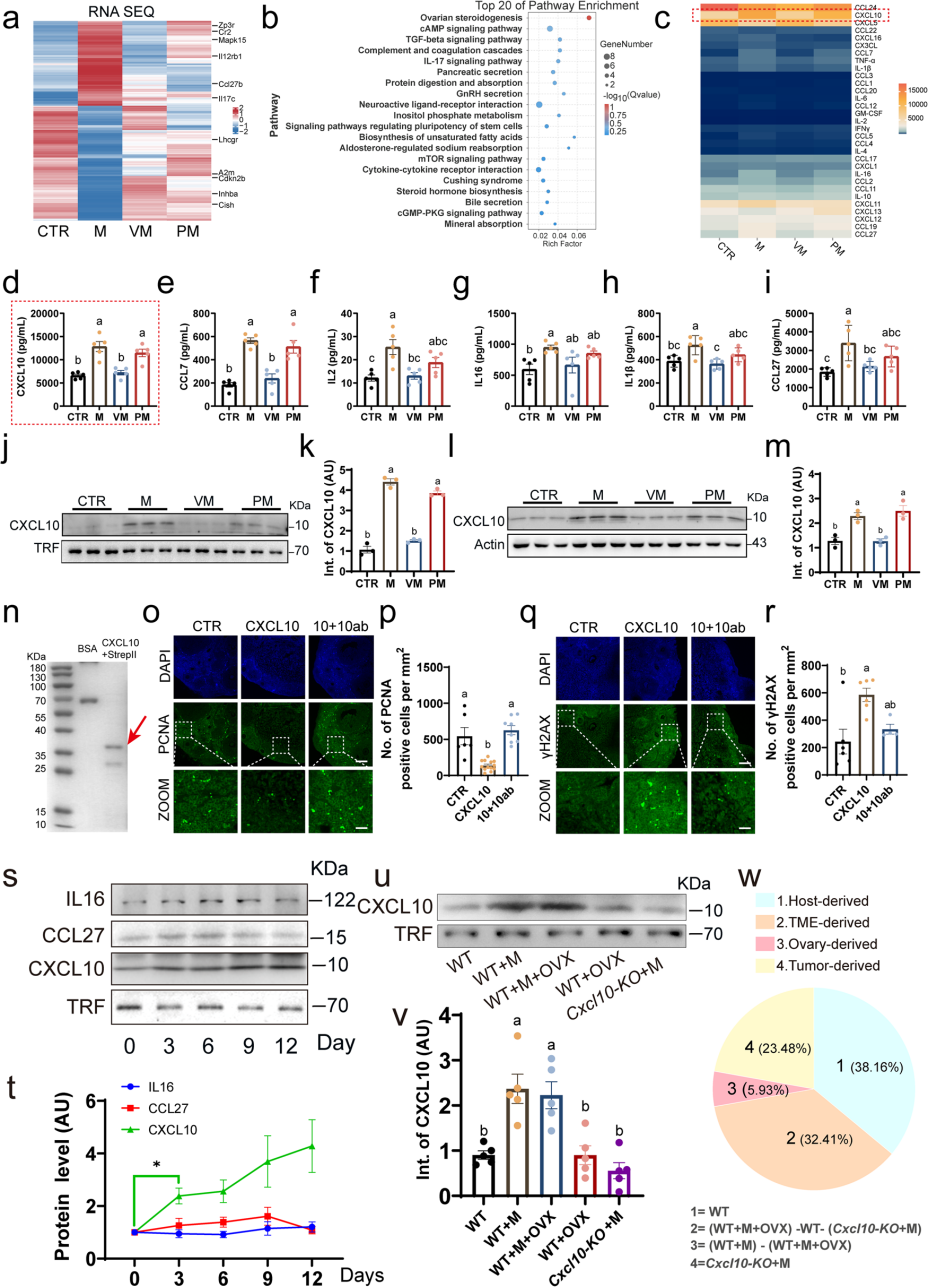
CXCL10 is the primary factor for impaired ovarian function upon NRTP. **a**, The RNA-sequencing heatmap revealed 435 differentially expressed genes between the control (CTR) group and the M group at the threshold of |log2(M/CTR)| ≥ 1. The expression profile in the VM group was very close to that in the CTR group, whereas the expression profile in the PM group was only somewhat close to that in the CTR group. **b**, The top 10 Kyoto Encyclopedia of Genes and Genomes pathways of the differentially expressed genes were involved mainly in inflammation (highlighted in the red dotted rectangle). **c**, Detection of plasma cytokine protein levels via a cytokine microarray revealed that among these cytokines, CXCL10 was the most upregulated in the M group (red dotted rectangle), and whole cancer cell vaccine treatment significantly rescued the CXCL10 level in the VM group, whereas anti-PD-1 antibody treatment did not affect the CXCL10 level in the PM group. **d**–**i**, Levels of multiple representative cytokines from microarray assay, *N* = 5 for all groups. For CXCL10 (part **d**), CTR versus M, *P* < 0.0001; CTR versus PM, *P* = 0.0012; M versus VM, *P* = 0.0003; VM versus PM, *P* = 0.0042. CXCL10 was the most highly upregulated gene in the M group (red dotted rectangle), which was effectively rescued in the VM group. For CCL7 (part **e**), CTR versus M, *P* < 0.0001; CTR versus PM, *P* < 0.0001; M versus VM, *P* < 0.0001; VM versus PM, *P* = 0.0004. For IL2 (part **f**), CTR versus M, *P* = 0.0027; M versus VM, *P* = 0.005. For IL16 (part **g**), CTR versus M, *P* = 0.0316. For IL-1β (part **h**), CTR versus M, *P* = 0.0122; M versus VM, *P* = 0.0034. For CCL27 (part **i**), CTR versus M, *P* = 0.003; M versus VM, *P* = 0.0159. **j**–**m**, Western blot and quantification verified that CXCL10 was significantly increased in both the plasma (parts **j** and **k**) and ovaries (parts **l** and **m**) in the M group. Whole cancer cell vaccine treatment significantly rescued the CXCL10 level in the VM group, whereas anti-PD-1 antibody treatment had no rescue effect in the PM group. *N* = 3 for all groups. For the CXCL10 levels in the serum, CTR versus M, *P* < 0.0001; CTR versus PM, *P* < 0.0001; M versus VM, *P* < 0.0001; VM versus PM, *P* < 0.0001. For CXCL10 levels in the ovaries, CTR versus M, *P* = 0.0078; CTR versus PM, *P* = 0.0025; M versus VM, *P* = 0.0074; and VM versus PM, *P* = 0.0024. **n**, Coomassie blue staining of purified recombinant GST-CXCL10-StrepII protein (arrow). Bovine serum albumin (BSA) (0.4 mg/ml) was used as a reference to determine the concentration of recombinant CXCL10. **o**–**r**. Immunofluorescence and quantification revealed that treating in vitro-cultured ovaries with recombinant CXCL10 protein led to decreased proliferation (indicated by proliferating cell nuclear androgen (PCNA), parts **o** and **p**) and increased double-strand breaks (indicated by γH2AX, parts **q** and **r**), whereas co-treatment with anti-CXCL10 antibodies significantly rescued the levels of PCNA and γH2AX. For PCNA, *N* = 13 for the CXCL10 group; *N* = 6 for the CTR group; *N* = 8 for the 10+10ab group. CTR versus CXCL10, *P* = 0.0002; CXCL10 versus 10+10ab, *P* < 0.0001. For γH2AX, *N* = 6 for the CTR and CXCL10 groups; *N* = 4 for the 10+10ab groups. CTR versus CXCL10, CTR versus CXCL10, *P* = 0.0069. PCNA or γH2AX is shown in green, and DNA is shown in blue. Scale bars in parts **o** and **q**, 100 µm; scale bars in zoom, 25 µm. **s**,**t**, Western blot and quantification verified that plasma CXCL10 level significantly increased on day 3 of in MCA205 tumor progression group, whereas IL16 or CCL27 did not show a significant trend. Plasma-level comparison between day 3 and day 0 of MCA205 TP, for CXCL10, *P* = 0.0194. *N* = 4 for all cytokines at each time point. **u**–**w**, Parallel CXCL10 western blot on the plasma of wild-type (WT), tumor-bearing WT, tumor-bearing ovariectomized WT, ovariectomized WT, and tumor-bearing *Cxcl10*‑KO mice on day 12 of tumor transplantation. Quantification indicated that in tumor-bearing WT mice, host-derived, tumor microenvironment (TME)-derived, tumor-derived, and ovary-derived CXCL10 accounted for 38.16%, 32.4%, 23.8%, and 5.93% of total plasma CXCL10, respectively. *N* = 5 for each group. Unpaired two-tailed *t*test is used for statistical comparison between two groups, one-way analysis of variance is used for comparisons between multiple (three or more) groups in the graphs, different letters above the columns indicate significant differences. For western blot and cytokine microarray, the number of independent biological repeats (R) was the same as technical repeats (*N*); for all other experiments, the number of independent biological repeats (R) was 3. AU, arbitrary unit; DAPI, 4’,6-diamino-2-phenylpyridine; IFN, interferon; NRTP, non-reproductive tumor progression; TGF-β, transforming growth factor-beta; TNF, tumor necrosis factor.

The distinct effects of WCV and PD-1 immunotherapies on ovaries under NRTP provided a better opportunity for us to discover the key factor through which mouse fibrosarcoma damages ovaries. Ovarian RNA sequencing revealed more than 400 DEGs between the M group and the control group at the threshold of |log2(M/CTR)| ≥ 1. Principal component analysis showed that the cluster of all three repeats in the M group almost completely separates from the CTR group, the cluster of VM group has the largest overlap with CTR group, and the cluster of PM group has some overlap with both M and CTR groups (Supplementary Fig. 2a). The DEG profile of the WCV group was more similar to that of the control group, whereas the DEG profile of the PD-1 group was different from that of both the control group and the VM group (Fig. 3a, Supplementary Fig. 2b, and Supplementary Dataset 1). Kyoto Encyclopedia of Genes and Genomes (KEGG) analysis of the DEGs revealed that many were involved in inflammation (Fig. 3b).

Many studies have demonstrated that increased inflammation impairs ovarian function^16–18^; therefore, we focussed on inflammation-promoting cytokines next. We used a microarray including 31 cytokines to detect changes in the levels of these cytokines in the control, M, VM, and PM groups. Among these cytokines, the absolute concentration of CXCL10 was significantly greater than that of the other cytokines, and the most upregulated cytokine in the M group was CXCL10. WCV treatment reduced the CXCL10 level close to that of the control in the VM group, whereas PD-1 antibody treatment did not affect CXCL10 protein level in the PM group (Fig. 3c–i, Supplementary Fig. 2c, Supplementary Fig. 3a–e1, red dotted-line rectangle, and Supplementary Dataset 2). We verified this change in both the plasma (Fig. 3j,k) and the ovaries (Fig. 3l,m). We subsequently used purified CXCL10 (Fig. 3n) to treat in vitro-cultured ovaries and found that CXCL10 significantly decreased proliferation (indicated by PCNA) (Fig. 3o,p and Supplementary Fig. 15c) and increased DNA double-strand breaks (indicated by γH2AX) (Fig. 3q,r and Supplementary Fig. 15d). Anti-CXCL10 antibody treatment completely reversed these changes, suggesting that CXCL10 is a key cytokine impairing ovarian function. Finally, we examined the serum levels of CXCL10, IL16, and CCL27, which are the richest within the plasma, at different time points of NRTP. Results showed that CXCL10 levels elevated fastest on days 3, 6, 9, and 12 of tumor bearing in M group, whereas the increment speed of IL16 and CCL27 was much slower (Fig. 3s,t). This suggested that CXCL10 is predominant over other cytokines to be a potential novel marker indicating the NRTP-induced early damage on ovaries.

Another question concerns the source of increased plasma CXCL10 in tumor-bearing mice. To investigate this, we utilized ovariectomized and *Cxcl10*-KO mice. A logical model was established as follow: (1) total plasma CXCL10 in tumor-bearing mice is from the host (the recipient mouse), the tumor, the TME (tumor microenvironment), and the ovary; (2), tumor-derived plasma CXCL10 = the plasma CXCL10 in tumor-bearing *Cxcl10*-KO mice; (3), TME-derived plasma CXCL10 = (plasma CXCL10 of tumor-bearing ovariectomy mice) – (tumor-derived plasma CXCL10) – (plasma CXCL10 of control mice); (4) ovary-derived plasma CXCL10 = (plasma CXCL10 of WT mice) – (plasma CXCL10 of ovariectomy mice). On the basis of the upper model, we did parallel CXCL10 western blot on the plasma of wild-type (WT), tumor-bearing WT, tumor-bearing ovariectomized WT, ovariectomized WT, and tumor-bearing *Cxcl10*‑KO mice on day 12 of tumor transplantation. Quantification indicated that in tumor-bearing WT mice, host-derived, TME-derived, tumor-derived, and ovary-derived CXCL10 accounted for 38.16%, 32.4%, 23.8%, and 5.93% of total plasma CXCL10, respectively (Fig. 3u–w). This indicates that tumor and TME together (56.2%) are responsible for nearly all the elevated CXCL10 observed in the tumor-bearing group.

### Comprehensive analysis of gene expression patterns in NRPT-induced ovary damage

**Fig. 4:**
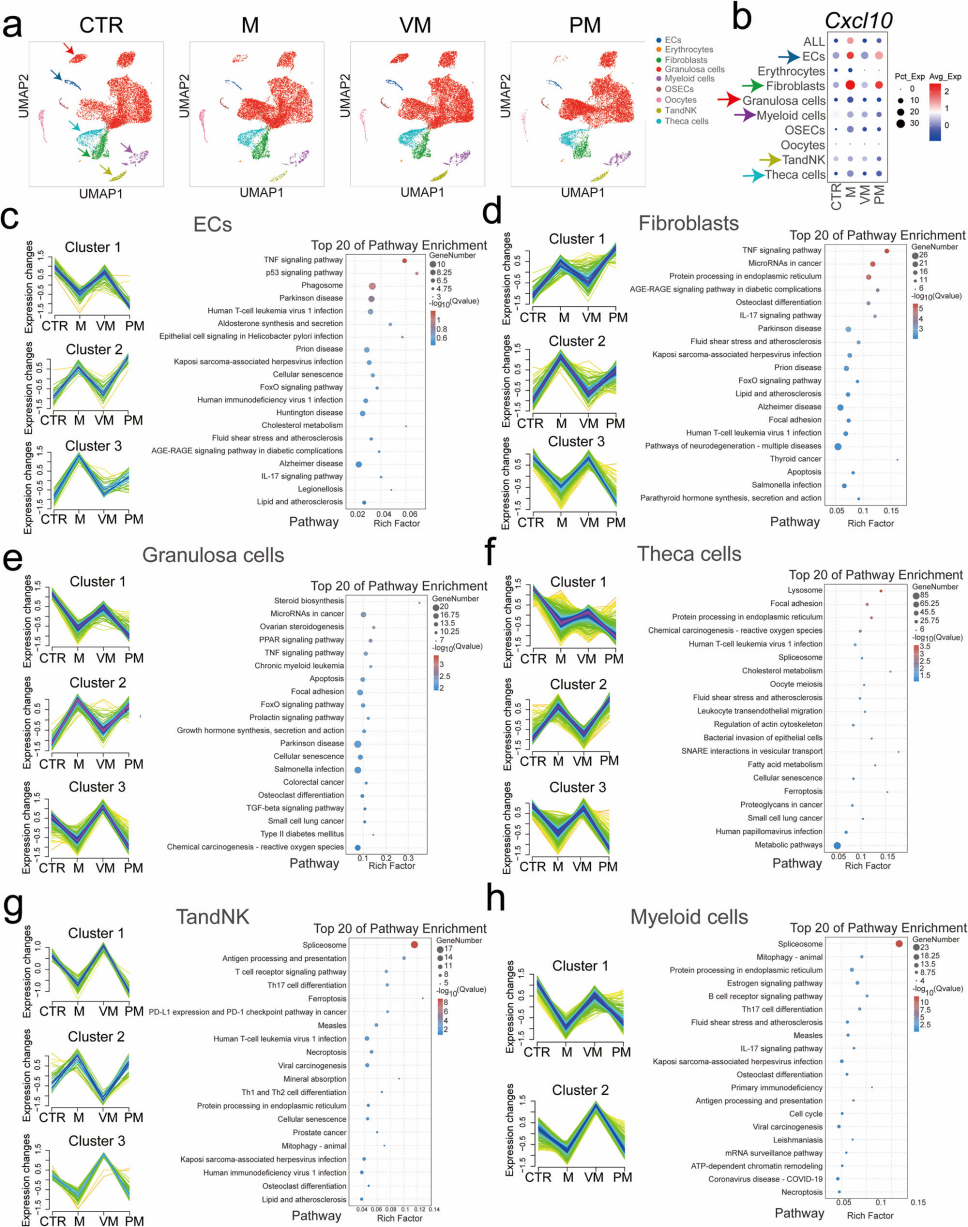
Comprehensive analysis of gene expression patterns in NRTP-induced ovary damage. **a**, Single-cell RNA-sequencing analysis of ovarian samples from the control (CTR), M, VM, and PM groups. In total, more than 10,000 qualified cells per group were collected, cells of each group were randomly clustered into 23 subclusters and annotated into 9 major cell types, namely, granulosa cells (GCs), fibroblasts, theca cells, endothelial cells (ECs), ovarian surface epithelial cells (OSECs), oocytes, myeloid cells, erythrocytes, T and NK cells, and myeloid cells, among which T and NK cells and myeloid cells are immune cells. **b**, In six (out of nine) types of cells (ECs, fibroblasts, GCs, theca cells, T and NK cells, and myeloid cells), Cxcl10 significantly increased in M group, was rescued by whole cancer cell vaccine treatment in VM group, whereas it was not changed by PD-1 antibody treatment in PM. **c–h**, Many differentially expressed genes within the upper six types of cells showed similar trend: upregulated and downregulated in M group, rescued by whole cancer cell vaccine but not by PD-1 antibody. These genes were involved in many important pathways that could regulate fibrosis, follicle activation, and inflammation. The threshold values of log2(M/CTR), log2(VM/M), and log2(PM/VM) are ≥ 0.25 or ≤ −0.25. NRTP, non-reproductive tumor progression; PPAR, peroxisome proliferator-activated receptor; TGF-β, transforming growth factor-beta; TNF, tumor necrosis factor; UMAP, uniform manifold approximation and projection.

To comprehensively understand how NRTP caused ovary damage, WCV treatment significantly rescues NRTP-induced ovarian damage, and PD-1 antibody treatment does not, we performed scRNA-seq analysis of ovarian samples from the CTR, M, VM, and PM groups. In total, more than 10,000 qualified cells per group were collected, cells of each group were randomly clustered into 23 subclusters and annotated into 9 major cell types, namely, granulosa cells (GCs), fibroblasts, theca cells, endothelial cells (ECs), ovarian surface epithelial cells (OSECs), oocytes, myeloid cells, erythrocytes, T and natural killer (NK) cells, and myeloid cells, among which T and NK cells and myeloid cells are immune cells (Fig. 4a). Notably, in six (out of nine) types of cells (ECs, fibroblasts, GCs, theca cells, T and NK cells, and myeloid cells), Cxcl10 significantly increased in M group, was rescued by WCV treatment in VM group, whereas it was not changed by PD-1 antibody treatment in PM at all (Fig. 4b). Moreover, many DEGs within these six types of cells showed similar trend: upregulated and downregulated in M group, rescued by WCV but not by PD-1 antibody. These DEGs were involved in many important pathways that could regulate fibrosis, follicle activation, and inflammation (Fig. 4c–h).

These results indicated that the rescue effect of WCV is extensive and support that the CXCL10 level was largely well rescued by WCV.

### IL18R1 is the dominant CXCL10 receptor in the ovaries

**Fig. 5:**
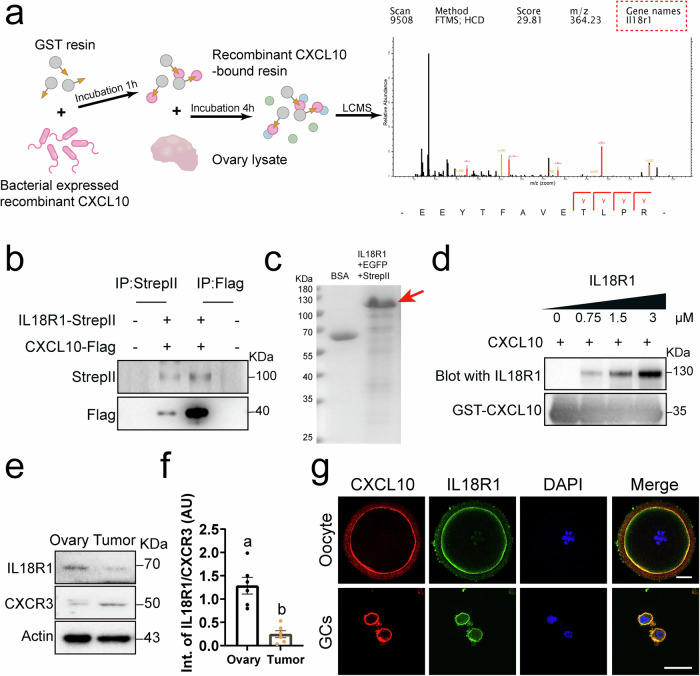
IL18R1 is the dominant CXCL10 receptor in the ovaries. **a**, The purified GST-CXCL10 protein was immobilized onto glutathione agarose beads and then incubated with ovarian lysates to identify potential CXCL10-interacting proteins in the ovaries via liquid chromatography–mass spectrometry (LC–MS). IL18R1 was the only membrane receptor protein identified. **b**, Co-transfection of pcDNA3.1-IL18R1-EGFP-StrepII and pcDNA3.1-CXCL10-TagRFP-Flag plasmids into MCA205 cells followed by co-immunoprecipitation (co-IP) revealed the interaction between IL18R1 and CXCL10 in vivo. **c**, Coomassie blue staining showing the purity of the recombinant IL18R1-EGFP-StrepII protein expressed through the bac-to-bac system (arrow). Bovine serum albumin (BSA) (0.4 mg/ml) was used as a reference to determine the concentration of the recombinant IL18R1. **d**, In vitro pull-down experiments revealed that when an increased amount of recombinant IL18R1 protein was added to the mixture, the addition of recombinant GST-CXCL10 (Fig. 3n) coupled with glutathione agarose beads increased the amount of recombinant IL18R1. **e**,**f**, Blotting and quantification revealed that the level of IL18R1/CXCR3 is fivefold greater in ovaries than in tumors; *N* = 6 for all groups; *P* = 0.0003. **g**, Immunofluorescence showed that when recombinant Strep II-tagged, CXCL10 protein was added to culture media containing oocytes or granulosa cells (GCs), CXCL10 and IL18R1 colocalized on the membranes. CXCL10 is in red, and IL18R1 is in green. Unpaired two-tailed *t*test is used for statistical comparison between two groups. Scale bars in part **g**, 20 µm. For western blot, the number of independent biological repeats (R) was the same as technical repeats (*N*); for all other experiments, the number of independent biological repeats (R) was 3. AU, arbitrary unit; DAPI, 4’,6-diamino-2-phenylpyridine.

CXCR3 is a CXCL10 receptor expressed on CD8^+^ T cells^18^, regulatory T cells^19^, resident memory B cells^20^, and tumor cells^21^. CXCL10 binding to CXCR3 mediates the recruitment of CXCR3-expressing cells to tumors or infected regions^22^; however, the impact of CXCL10 on ovaries appears to differ. Therefore, we hypothesized that another receptor dominating CXCR3 might induce ovarian damage. We bound recombinant GST-CXCL10 to glutathione agarose resin, incubated the resin with ovarian lysates, and then conducted mass spectrometry. We characterized 61 proteins that may interact with CXCL10, among which IL18R1 was the only receptor (Fig. 5a and Supplementary Dataset 3). We verified the interaction between CXCL10 and IL18R1 through co-IP (Fig. 5b). Furthermore, we found that as an increasing amount of recombinant IL18R1 (Fig. 5c) was added, GST-CXCL10 bound to glutathione agarose resin pulled down increasing amounts of IL18R1 (Fig. 5d).

Interestingly, we did not bait CXCR3, the known CXCL10 receptor expressed by T cells, as the CXCL10-interacting protein in the ovaries. We examined the relative expression levels of CXCL10 and IL18R1 in the ovaries and MCA205 tumors and found that the IL18R1:CXCR3 ratio in the ovaries was six fold greater than that in the MCA205 tumors, indicating that IL18R1 dominates over CXCR3 in binding to CXCL10 in the ovaries (Fig. 5e,f). In addition, we found that recombinant CXCL10-Strep II colocalized with IL18R1 on the membrane of both oocytes and GCs (Fig. 5g).

### NRTP induced fibrosis through the CXCL10–IL18R1 → COL1A1 pathway

**Fig. 6:**
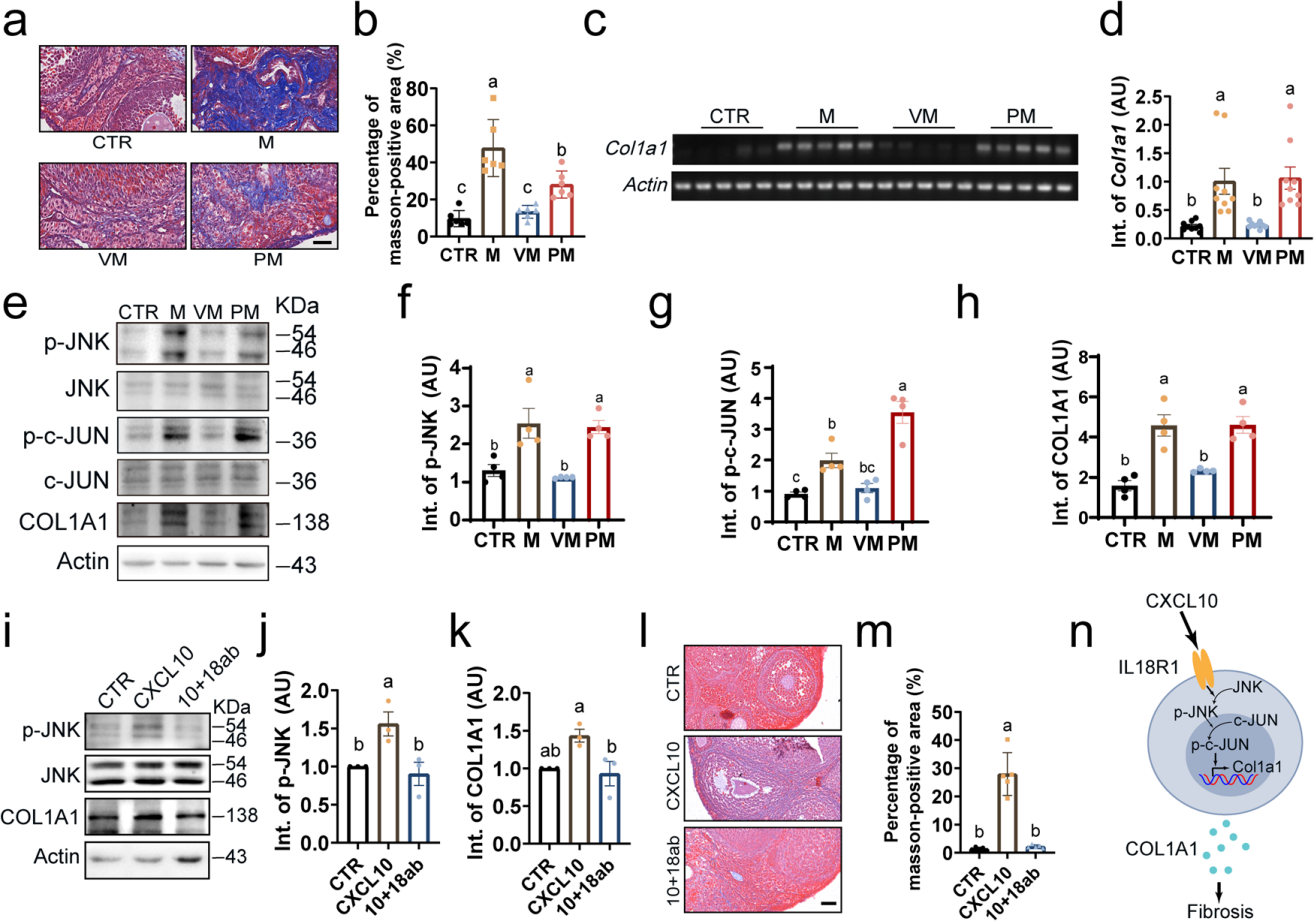
NRTP induced fibrosis through the CXCL10–IL18R1 → COL1A1 pathway. **a**,**b**, Masson staining and quantification revealed that fibrosis, as indicated by the percentage of the collagen fraction (blue staining), significantly increased in the M group. Whole cancer cell vaccine (WCV) treatment significantly rescued the fibrosis level in the VM group, whereas anti-PD-1 antibody treatment had no significant rescue effect in the PM group; *N* = 6 for all groups. Control (CTR) versus M, *P* < 0.0001; CTR versus PM, *P* = 0.0097; M versus VM, *P* < 0.0001; M versus PM, *P* = 0.0055; VM versus PM, *P* = 0.0449. **c**,**d**, RT-PCR and quantification revealed that the mRNA level of *Col1a1*, which encodes the collagen protein α1(I) chain, was significantly increased in the M group. WCV treatment significantly rescued *Col1a1* level in the VM group, whereas anti-PD-1 antibody treatment had no significant rescue effect in the PM group. *N* = 9 for all groups. CTR versus M, *P* = 0.0044; CTR versus PM, *P* = 0.0021; M versus VM, *P* = 0.0056; VM versus PM, *P* = 0.0027. **e**–**h**, Western blot and quantification revealed that the levels of COL1A1 and its upstream regulators, p-JNK and p-JUN, significantly increased in the M group. WCV treatment significantly rescued their levels in the VM group, whereas anti-PD-1 antibody treatment had no significant rescue effect in the PM group. *N* = 4 for all groups. For p-JNK, CTR versus M, *P* = 0.0108; CTR versus PM, *P* = 0.0184; M versus VM, *P* = 0.0039; VM versus PM, *P* = 0.0067. For p-JUN, CTR versus M, *P* = 0.0275; CTR versus PM, *P* < 0.0001; M versus PM, *P* = 0.002; VM versus PM, *P* < 0.0001. For COL1A1, CTR versus M, *P* = 0.0004; CTR versus PM, *P* = 0.0004; M versus VM, *P* = 0.0042; VM versus PM, *P* = 0.0038. **i**–**k**, Western blot and quantification revealed that in in vitro-cultured ovaries, recombinant CXCL10 significantly increased p-JNK and COL1A1 levels, whereas anti-IL18R1 antibody co-treatment significantly rescued p-JNK and COL1A1 levels. *N* = 3 for all groups. For p-JNK, CTR versus CXCL10, *P* = 0.0463; CXCL10 versus 10+18ab, *P* = 0.0243. For COL1A1, CXCL10 versus 10+18ab, *P* = 0.0343. **l**,**m**, Masson staining and quantification revealed that in the in vitro-cultured ovaries, recombinant CXCL10 significantly increased the degree of fibrosis, whereas anti-IL18R1 antibody co-treatment significantly rescued the degree of fibrosis. *N* = 5 for all groups. CTR versus CXCL10, *P* < 0.0001; CXCL10 versus 10+18ab, *P* < 0.0001. **n**, Model. CXCL10 binds the membrane receptor IL18R1 in oocytes and granulosa cells, subsequently activates p-JNK and p-JUN, and finally promotes the transcription of *Col1a1* and ovarian fibrosis. Scale bars in parts **a** and **l**, 50 µm. One-way analysis of variance is used for statistical comparisons between multiple (three or more) groups in the graphs, different letters above the columns indicate significant differences. For western blot, the number of independent biological repeats (R) was the same as technical repeats (*N*); for all other experiments, the number of independent biological repeats (R) was 3. AU, arbitrary unit; NRTP, non-reproductive tumor progression.

Fibrosis severely impairs ovarian function and is the main feature of reproductive aging^23^ and polycystic ovary syndrome^24^; in a previous study, antifibrosis drugs reinstated ovulation in reproductively aged and obese mice by eradicating fibrotic collagen^25^. IL18R1 was shown to promote COL1A1 expression through JNK^26^. Therefore, we hypothesized that CXCL10 could induce fibrosis through IL18R1. Our results revealed that NRTP significantly increased fibrosis in the M group; WCV treatment reduced fibrosis close to that of the control in the VM group, whereas PD-1 antibody treatment did not affect fibrosis in the PM group (Fig. 6a,b and Supplementary Fig. 4). Accordingly, the transcription levels of *Col1a1* significantly increased in the M group; although WCV treatment reduced *Col1a1* close to that of the control in the VM group, PD-1 antibody treatment did not have any rescue effects in the PM group (Fig. 6c,d and Supplementary Table 3).

JNK and JUN, which regulate *Col1a1* transcription in the ovaries, are downstream proteins of IL18R1^26^. COL1A1, p-JUN, and p-JNK were significantly elevated in the ovaries of the M group; WCV treatment reduced the levels of these proteins close to those of the control in the VM group, whereas PD-1 antibody treatment did not rescue the levels of these proteins in the PM group (Fig. 6e–h).

Next, to further verify the connection between CXCL10 and IL18R1 in fibrosis induction, we compared the effects of recombinant CXCL10 treatment (CXCL10 group) and CXCL10 plus IL18R1 antibody treatment (10+18ab) in in vitro-cultured ovaries. Western blot analysis revealed that recombinant CXCL10 treatment sharply increased the levels of COL1A1, p-JUN, and p-JNK in the CXCL10 group, whereas IL18R1 antibody treatment reversed their levels close to those of the control in the 10+18ab group (Fig. 6i–k). Accordingly, Masson staining revealed that recombinant CXCL10 treatment sharply increased fibrosis in the CXCL10 group, whereas IL18R1 antibody treatment reversed fibrosis to levels close to those of the control in the 10+18ab group (Fig. 6l,m).

Moreover, p-JUN inhibitor (p-JUNi) treatment could also rescue CXCL10-induced COL1A1 upregulation, which further validates the downstream fibrosis pathway (Supplementary Fig. 5a–f).

These results indicate that in the ovaries, CXCL10 binds to IL18R1 to induce downstream JNK→JUN activation, which subsequently promotes the transcription of COL1A1 and ovarian fibrosis (Fig. 6n).

### NRTP caused primordial follicle decrement through the CXCL10–IL18R1 → AKT pathway

A decrease in PMFs is one of the main causes of reduced female fertility, and AKT overactivation is one of the main mechanisms for a decrease in PMFs^27^. IL18R1 has also been shown to promote AKT activation^28^. Therefore, we hypothesized that CXCL10 could induce a decrease in PMFs through IL18R1.

**Fig. 7:**
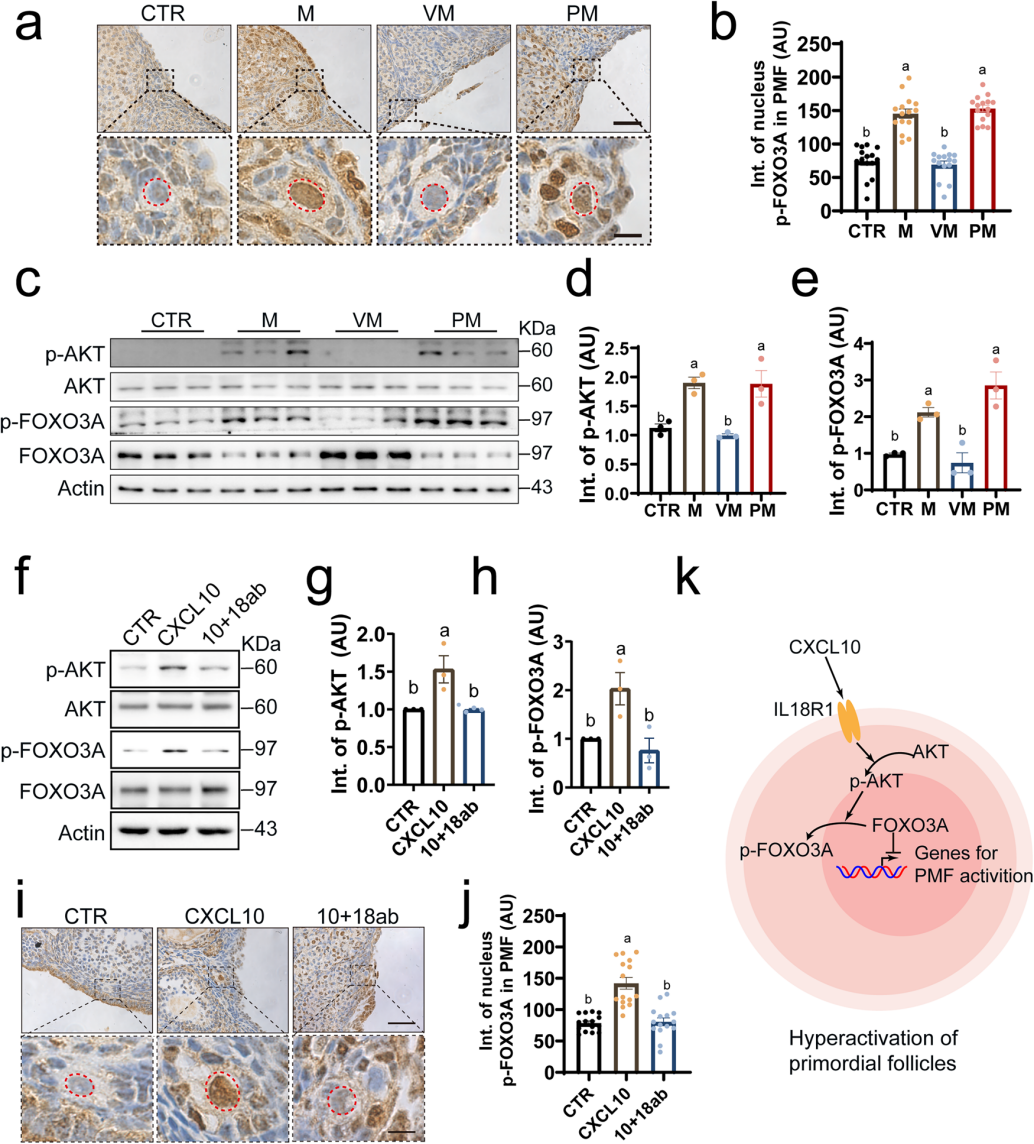
NRTP caused primordial follicle decrement through the CXCL10–IL18R1 → AKT pathway. **a**,**b**, p-FOXO3A immunohistochemistry of ovarian paraffin sections revealed that nuclear p-FOXO3A (red dotted-line circle), which indicates primordial follicle (PMF) activation, significantly increased in the M group; whole cancer cell vaccine (WCV) treatment significantly rescued the nuclear p-FOXO3A level in the VM group, whereas anti-PD-1 antibody treatment had no significant rescue effect in the PM group. *N* = 15 for all groups. Control (CTR) versus M, *P* < 0.0001; CTR versus PM, *P* < 0.0001; M versus VM, *P* < 0.0001; VM versus PM, *P* < 0.0001. **c**–**e**, Western blot and quantification revealed that p-FOXO3A and its upstream regulator p-AKT were significantly increased in the M group; WCV treatment significantly rescued their levels in the VM group, whereas anti-PD-1 antibody treatment had no significant rescue effect in the PM group. *N* = 3 for all groups. For p-AKT, CTR versus M, *P* = 0.0125; CTR versus PM, *P* = 0.0139; M versus VM, *P* = 0.005; VM versus PM, *P* = 0.0056. For p-FOXO3A, CTR versus M, *P* = 0.0381; CTR versus PM, *P* = 0.0023; M versus VM, *P* = 0.0148; VM versus PM, *P* = 0.0011. **f**–**h**, Western blot and quantification revealed that in in vitro-cultured ovaries, recombinant CXCL10 significantly increased p-FOXO3A and p-AKT levels, whereas anti-IL18R1 antibody co-treatment significantly rescued their levels. *N* = 3 for all groups. For p-AKT, CTR versus CXCL10, *P* = 0.0265; CXCL10 versus 10+18ab, *P* = 0.0249. For p-FOXO3A, CXCL10 versus 10+18ab, *P* = 0.0227. **i**,**j**, p-FOXO3A immunohistochemistry of ovarian paraffin sections and quantification revealed that in in vitro-cultured ovaries, recombinant CXCL10 significantly increased the nuclear p-FOXO3A intensity in PMFs, whereas anti-IL18R1 antibody co-treatment significantly rescued the p-FOXO3A level. *N* = 15 for all groups. CTR versus CXCL10, *P* < 0.0001; CXCL10 versus 10+18ab, *P* < 0.0001. **k**, Model. CXCL10 binds the membrane receptor IL18R1 of oocytes and granulosa cells, subsequently activates p-AKT and p-FOXO3A, and finally promotes the transcription of genes for PMF activation. Scale bars in parts **a** and **i**, 100 µm; scale bars in zoom, 20 µm. One-way analysis of variance is used for statistical comparisons between multiple (three or more) groups in the graphs; different letters above the columns indicate significant differences. For western blot, the number of independent biological repeats (R) was the same as technical repeats (*N*); for all other experiments, the number of independent biological repeats (R) was 3. AU, arbitrary unit; NRTP, non-reproductive tumor progression.

A previous study revealed that p-AKT can enter the nucleus and phosphorylate FOXO3A, which promotes the expression of genes necessary for PMF activation^29^. Immunohistochemistry revealed that NRTP significantly increased nuclear p-FOXO3A in ovarian PMFs in the M group. WCV treatment reduced the level of nuclear p-FOXO3A close to the control level in the VM group, whereas PD-1 antibody treatment did not rescue nuclear p-FOXO3A levels in the PM group (Fig. 7a,b). Similarly, western blot analysis revealed that NRTP significantly increased p-AKT and p-FOXO3A in the M group, and WCV treatment reduced their levels close to those of the control in the VM group, whereas PD-1 antibody treatment did not have a rescue effect on the PM group (Fig. 7c–e).

Next, to further verify the connection between CXCL10 and IL18R1 for PMF activation, we compared recombinant CXCL10 treatment and CXCL10 plus IL18R1 antibody treatment (10+18ab) in in vitro-cultured ovaries. Blot analysis revealed that recombinant CXCL10 treatment sharply increased the levels of p-AKT and p-FOXO3A in the CXCL10 group, whereas IL18R1 antibody treatment reversed their levels close to those of the control in the 10+18ab group (Fig. 7f–h). Accordingly, immunohistochemistry revealed that recombinant CXCL10 treatment sharply increased the level of p-FOXO3A in the nucleus of PMFs from the CXCL10 group, whereas IL18R1 antibody treatment reversed this increase to levels close to those of the control in the 10+18ab group (Fig. 7i,j).

Moreover, p-AKT inhibitor treatment could also rescue CXCL10-induced p-FOXO3A upregulation, which further validates the downstream follicle activation pathway (Supplementary Fig. 6a–e).

The above results indicate that in the ovaries, CXCL10 can bind to IL18R1 to induce downstream AKT → FOXO3A activation, which subsequently promotes the transcription of genes for PMF activation (Fig. 7k).

### NRTP induced inflammation through the CXCL10–IL18R1 → P65 pathway

Cancer tissues induce the production and release of various cytokines, many of which are pro-inflammatory^3,30^. Cytokines must bind corresponding membrane receptors that transduce pro-inflammatory signals^31^. IL18R1 has been shown to activate P65, which then translocates to the nucleus to promote the transcription of pro-inflammatory genes^32^; therefore, we hypothesized that CXCL10 could induce inflammation through IL18R1.

**Fig. 8:**
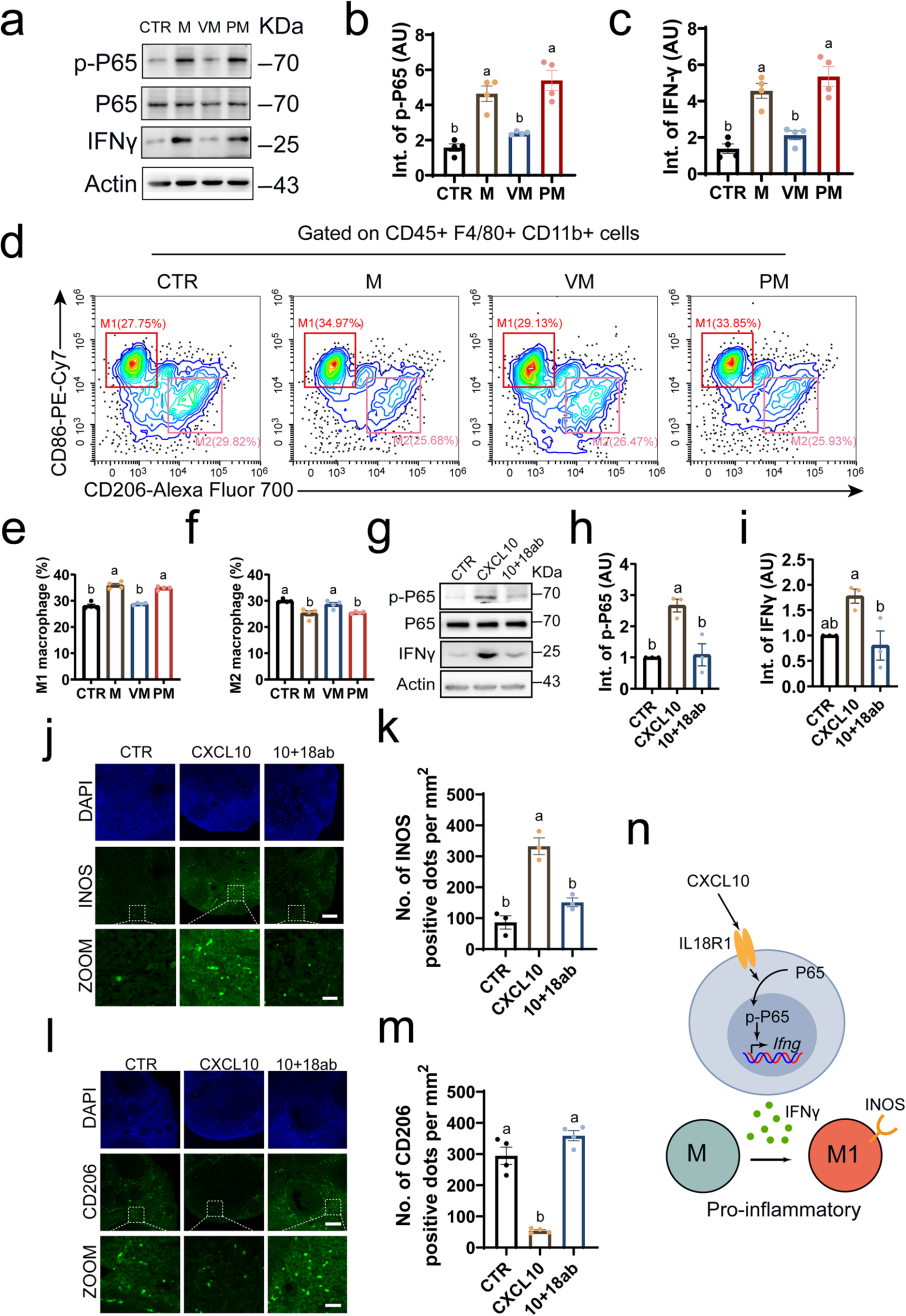
NRTP induced inflammation through the CXCL10–IL18R1 → P65 pathway. **a**–**c**, Western blot and quantification revealed that the levels of p-P65 and interferon-γ (IFNγ) significantly increased in the M group. Whole cancer cell vaccine (WCV) treatment significantly rescued their levels in the VM group, whereas anti-PD-1 antibody treatment had no significant rescue effect in the PM group. *N* = 4 for all groups. For p-P65, control (CTR) versus M, *P* = 0.0004; CTR versus PM, *P* < 0.0001; M versus VM, *P* = 0.0048; VM versus PM, *P* = 0.0005. For IFNγ, CTR versus M, *P* = 0.0005; CTR versus PM, *P* < 0.0001; M versus VM, *P* = 0.0021; VM versus PM, *P* = 0.0001. **d**–**f**, Fluorescence-activated cell sorting and quantification revealed that in the M group, the percentage of M1 macrophages significantly increased (34.97% in M and 27.75% in CTR), and the percentage of M2 macrophages significantly decreased (25.68% in M and 29.82% in CTR). WCV treatment significantly reduced the percentage of M1 macrophages (29.13% in WCV) but increased the percentage of M2 macrophages (26.47% in VM) close to that of the control in the VM group, whereas anti-PD-1 antibody treatment had no significant rescue effect in the PM group (M1, 33.85% and M2, 25.93%). *N* = 4 for all groups. For M1 macrophages, CTR versus M, *P* < 0.0001; CTR versus PM, *P* < 0.0001; M versus VM, *P* < 0.0001; VM versus PM, *P* < 0.0001. For M2 macrophages, CTR versus M, *P* = 0.0011; CTR versus PM, *P* = 0.0018; M versus VM, *P* = 0.0148; VM versus PM, *P* = 0.0242. **g**–**i**, Western blot and quantification revealed that in in vitro-cultured ovaries, recombinant CXCL10 significantly increased p-P65 and IFNγ levels, whereas anti-IL18R1 antibody co-treatment significantly rescued their levels. *N* = 3 for all groups. For p-p-65, CTR versus CXCL10, *P* = 0.0062; CXCL10 versus 10+18ab, *P* = 0.0079. For IFNγ, CXCL10 versus 10+18ab, *P* = 0.0229. **j**,**k**, Immunofluorescence and quantification revealed that in in vitro-cultured ovaries, recombinant CXCL10 treatment significantly increased the number of dots with inducible nitric oxide synthase (INOS), a marker of pro-inflammatory M1 macrophages, whereas anti-IL18R1 antibody co-treatment significantly rescued the number of INOS dots. INOS, green; 4′,6-diamidino-2-phenylindole (DAPI), blue. *N* = 3 for all groups. CTR versus CXCL10, *P* = 0.0005; CXCL10 versus 10+18ab, *P* = 0.0024. **l**,**m**, Immunofluorescence and quantification revealed that in in vitro-cultured ovaries, recombinant CXCL10 treatment significantly decreased the number of dots expressing CD206, a marker of anti-inflammatory M2 macrophages, whereas anti-IL18R1 antibody co-treatment significantly rescued the number of CD206 dots. CD206, green and DAPI, blue. *N* = 4 for all groups. CTR versus CXCL10, *P* = 0.0001; CXCL10 versus 10+18ab, *P* < 0.0001. **n**, Model. CXCL10 binds the membrane receptor IL18R1 on oocytes and granulosa cells, subsequently activates p-P65, and finally promotes the transcription of *Ifng*. Scale bars in parts **j** and **l**, 100 µm; scale bars in zoom, 20 µm. One-way analysis of variance is used for statistical comparisons between multiple (three or more) groups in the graphs, different letters above the columns indicate significant differences. For western blot and fluorescence-activated cell sorting, the number of independent biological repeats (R) was the same as technical repeats (*N*); for all other experiments, the number of independent biological repeats (R) was 3. AU, arbitrary unit; NRTP, non-reproductive tumor progression.

Western blot analysis revealed that NRTP significantly increased p-P65 and interferon-γ (IFNγ) levels in the ovaries of the M group; WCV treatment reduced these levels close to those of the control in the VM group, whereas PD-1 antibody treatment did not rescue these levels in the PM group (Fig. 8a–c). Furthermore, FACS and quantification (Supplementary Fig. 7 and Fig. 8d–f) revealed that in the M group, the percentage of M1 macrophages significantly increased (34.97% in the M group and 27.75% in the CTR group), and the percentage of M2 macrophages significantly decreased (25.68% in the M group and 29.82% in the CTR group). WCV treatment significantly reduced the percentage of M1 macrophages (29.13% in the WCV group) and increased the percentage of M2 macrophages (26.47% in the VM group), which was close to the control in the VM group, whereas anti-PD-1 antibody treatment had no significant rescue effect in the PM group (M1, 33.85% and M2, 25.93%).

Next, to further verify the connection between CXCL10 and IL18R1 in the induction of inflammation, we compared the effects of recombinant CXCL10 treatment and CXCL10 plus IL18R1 antibody treatment in in vitro-cultured ovaries. Western blot analysis revealed that recombinant CXCL10 treatment sharply increased the levels of p-P65 and IFNγ in the CXCL10 group, whereas IL18R1 antibody treatment reduced their levels close to those of the control in the 10+18ab group (Fig. 8g–i). Accordingly, we investigated the change in the number of macrophages in the ovaries. CXCL10 treatment significantly increased the level of the pro-inflammatory M1 macrophage marker, inducible nitric oxide synthase (INOS), in the CXCL10 group, whereas CXCL10 plus IL18R1 antibody treatment reduced the INOS level close to that of the control in the 10+18ab group (Fig. 8j,k and Supplementary Fig. 15e). By contrast, CXCL10 treatment significantly decreased the level of the anti-inflammatory M2 macrophage marker CD206 in the CXCL10 group, whereas CXCL10 plus IL18R1 antibody treatment elevated the CD206 level close to that of the control in the 10+18ab group (Fig. 8l,m and Supplementary Fig. 15f).

Moreover, p-P65 inhibitor treatment could also rescue CXCL10-induced IFNγ upregulation (Supplementary Fig. 8a–c), M1 macrophage increase (Supplementary Fig. 8d,e), and M2 macrophage decrease (Supplementary Fig. 8f,g) which further validate the downstream follicle activation pathway.

The aforementioned results indicated that in the ovaries, CXCL10 could bind to IL18R1 to activate downstream p-P65 → IFNγ signaling, which subsequently promoted the transformation of macrophages into pro-inflammatory type I macrophages (Fig. 8n).

### CXCL10 antibody injection rescued the NRTP-induced decrease in ovarian function

**Fig. 9:**
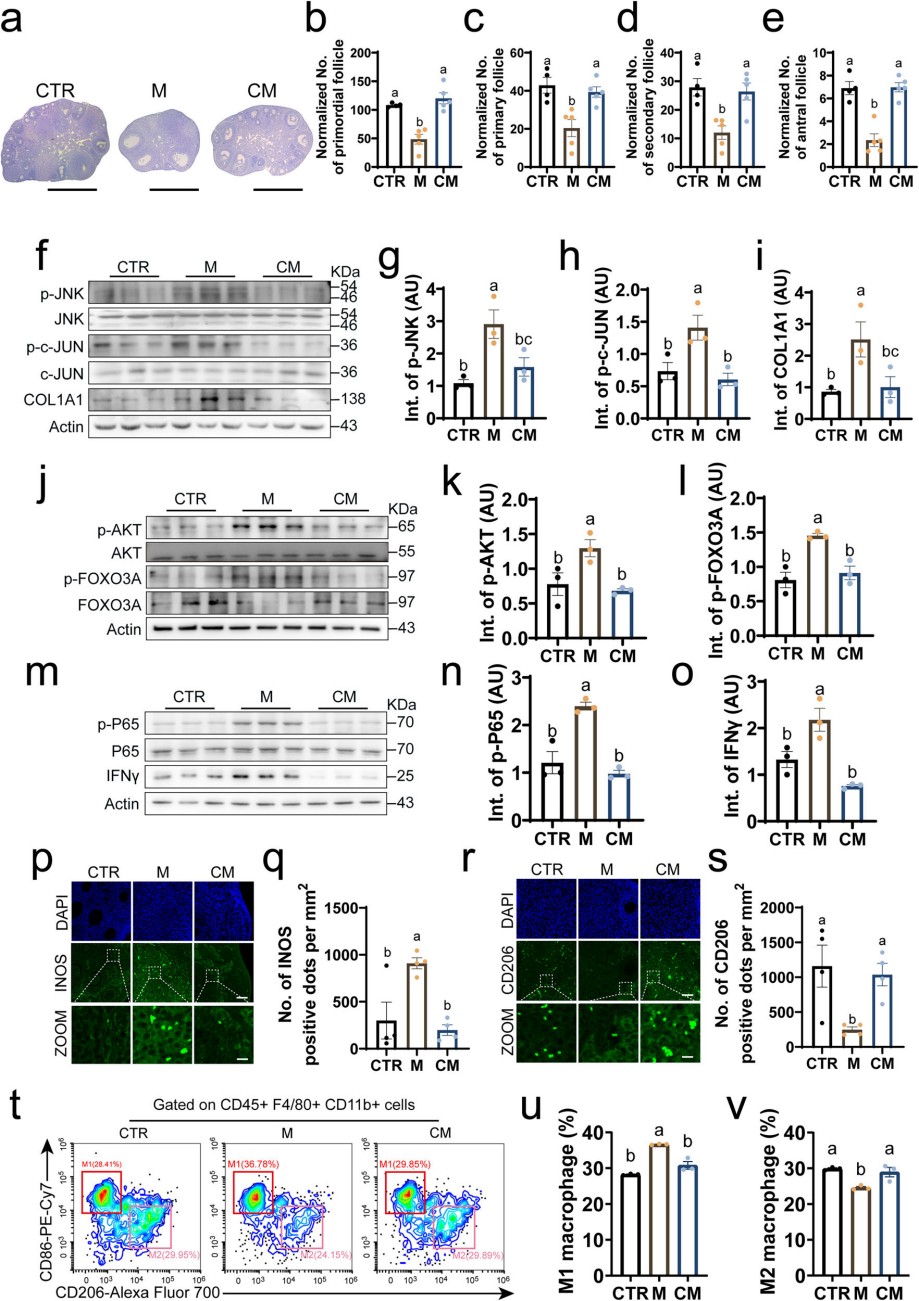
CXCL10 antibody injection rescued the NRTP-induced decrease in ovarian function. **a–e**, Hematoxylin and eosin staining and follicle counting revealed that the number of follicles at each stage significantly decreased in tumor-bearing mice in the M group, whereas anti-CXCL10 antibody treatment significantly rescued the number of follicles in the CM (anti-CXCL10 antibody plus MCA205 tumor) group. *N* = 4 for the control (CTR) group, *N* = 5 for the M and CM groups. For primordial follicles, CTR versus M, *P* = 0.0009; M versus CM, *P* = 0.0001. For primary follicles, CTR versus M, *P* = 0.0053; M versus CM, *P* = 0.011. For secondary follicles, CTR versus M, *P* = 0.0061; M versus CM, *P* = 0.0079. For antral follicles, CTR versus M, *P* < 0.0002; M versus CM, *P* = 0.0001. **f–i**, Western blotting and quantification revealed that the levels of p-JNK, p-JUN, and COL1A1 significantly increased in the M group, whereas anti-CXCL10 antibody treatment significantly rescued their levels in the CM group; *N* = 3 for all groups. For p-JNK, CTR versus M, *P* = 0.0138. For p-JUN, CTR versus M, *P* = 0.0398; M versus CM, *P* = 0.0193. For COL1A1, CTR versus M, *P* = 0.047. **j–l**, Western blotting and quantification revealed that the levels of p-AKT and p-FOXO3A significantly increased in the M group, whereas anti-CXCL10 antibody treatment significantly rescued their levels in the CM group. *N* = 3 for all groups. For p-AKT, CTR versus M, *P* = 0.0495; M versus CM, *P* = 0.0251. For p-FOXO3A, CTR versus M, *P* = 0.005; M versus CM, *P* = 0.0116. **m**–**o**, Western blotting and quantification revealed that the levels of p-P65 and interferon-γ (IFNγ) significantly increased in the ovaries of the M group, whereas anti-CXCL10 antibody treatment significantly rescued their levels in the CM group. *N* = 3 for all groups. For p-P65, CTR versus M, *P* = 0.031; M versus CM, *P* = 0.0012. For IFNγ, CTR versus M, *P* = 0.0312; M versus CM, *P* = 0.0029. **p**,**q**, Immunofluorescence and quantification revealed that the number of dots with inducible nitric oxide synthase (INOS), a marker of pro-inflammatory M1 macrophages, significantly increased in the M group, whereas anti-CXCL10 antibody treatment significantly rescued the number of INOS dots in the CM group. *N* = 4 for all groups. CTR versus M, *P* = 0.016; M versus CM, *P* = 0.0067. INOS, green; 4′,6-diamidino-2-phenylindole (DAPI), blue. **r**,**s**, Immunofluorescence and quantification revealed that the number of dots with CD206, a marker of anti-inflammatory M2 macrophages, significantly decreased in the M group, whereas anti-CXCL10 antibody treatment significantly rescued the number of CD206 dots in the CM group. *N* = 4 for all groups. CTR versus M, *P* = 0.0247; M versus CM, *P* = 0.0483. CD206, green; DAPI, blue. **t**–**v**, Fluorescence-activated cell sorting and quantification revealed that in the M group, the percentage of M1 macrophages significantly increased (36.78% in M and 28.41% in CTR), and the percentage of M2 macrophages significantly decreased (24.15% in M and 29.95% in CTR), whereas anti-CXCL10 antibody treatment significantly reduced the percentage of M1 macrophages (29.85% in CM) while increasing the percentage of M2 macrophages (29.89% in CM) in the CM group. *N* = 3 for all groups. For M1 macrophages, CTR versus M, *P* = 0.0002; M versus CM, *P* = 0.0012. For M2 macrophages, CTR versus M, *P* = 0.0081; M versus CM, *P* = 0.02. Scale bar in part **a**, 1 mm. Scale bars in parts **p** and **r**, 100 µm; scale bars in zoom, 20 µm. One-way analysis of variance is used for statistical comparisons between multiple (three or more) groups in the graphs, and different letters above the columns indicate significant differences. For western blot and fluorescence-activated cell sorting, the number of independent biological repeats (R) was the same as technical repeats (*N*); for all other experiments, the number of independent biological repeats (R) was 3. AU, arbitrary unit; NRTP, non-reproductive tumor progression.

Next, we explored whether CXCL10 inhibition via CXCL10 antibodies could rescue ovarian function. CXCL10 antibody injection significantly restored the number of follicles at each stage (Fig. 9a–e). Furthermore, we examined whether CXCL10 antibody injection could rescue the upper signal abnormalities caused by NRTP.

First, CXCL10 antibody treatment in the CM group reversed the MCA205 tumor-induced upregulation of p-JNK, p-JUN, and COL1A1 (Fig. 9f–i). In addition, CXCL10 antibody treatment in the CM group reversed the MCA205 tumor-induced increase in ovarian fibrosis (Supplementary Fig. 9a,b). Second, CXCL10 antibody treatment in the CM group reversed the MCA205 tumor-induced increase in p-AKT and p-FOXO3A levels (Fig. 9j–l). In addition, CXCL10 antibody treatment in the CM group reversed the MCA205 tumor-induced increase in nuclear p-FOXO3A in PMFs (Supplementary Fig. 9c,d). Third, CXCL10 antibody treatment in the CM group reversed the MCA205 tumor-induced increase in p-P65 and IFNγ levels (Fig. 9m–o). Additionally, immunofluorescence revealed that CXCL10 antibody treatment in the CM group significantly reversed the MCA205 tumor-induced increase in pro-inflammatory M1 macrophage levels (Fig. 9p,q and Supplementary Fig. 15g) and decrease in anti-inflammatory M2 macrophage levels (Fig. 9r,s and Supplementary Fig. 15h). Finally, FACS and quantification (Fig. 9t–v) revealed that in the M group, the percentage of M1 macrophages was significantly increased (36.78% in M and 28.41% in CTR), and the percentage of M2 macrophages was significantly reduced (24.15% in M and 29.95% in CTR), whereas anti-CXCL10 antibody treatment significantly reduced the percentage of M1 macrophages (29.85% in CM) and increased the percentage of M2 macrophages (29.89% in CM) close to the control in the CM group.

These results further indicate that CXCL10 is the key cytokine that induces ovarian damage and that CXCL10 antibodies can be used to prevent ovarian damage caused by NRTP.

### Recombinant CXCL10–IL18R1 interface peptide injection rescued the NRTP-induced decrease in ovarian function

**Fig. 10:**
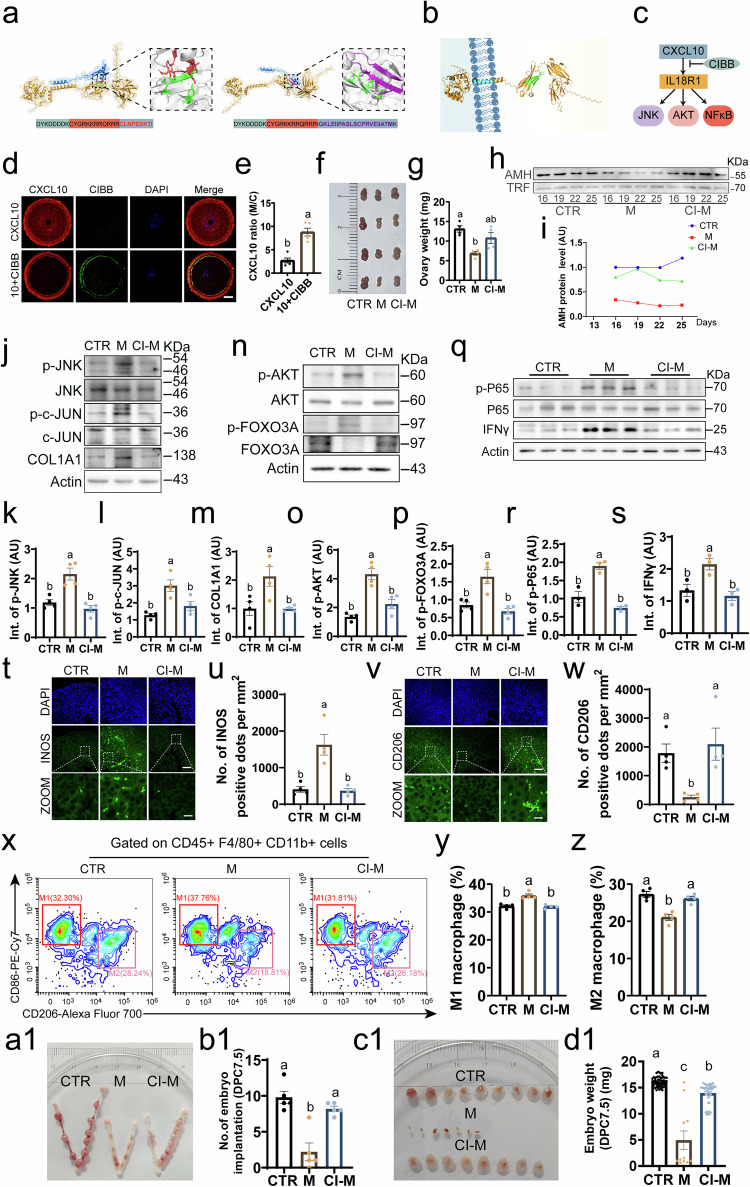
Recombinant CXCL10–IL18R1 interface peptide injection rescued the NRTP-induced decrease in ovarian function. **a**, The protein–protein interaction prediction by AlphaFold showed that the two interaction models had the highest scores. In model 1 (left), 74–82 animo acids (AAs) of CXCL10 bind near IL18R1; in model 2 (right), 45–67 AAs of CXCL10 bind near IL18R1. We fused Flag (DYKDDDDK) and TAT (CYGRKKRRQRRR) to these two peptides. We used these two compounds together as competitive peptides to inhibit the binding of CXCL10 to IL18R1. We named the combination of these peptides CIBB (CXCL10–IL18R1 binding blocker). **b**, Protein structure of IL18R1 across the phospholipid bilayer, with the transmembrane region shown in cyan and the region binding to CXCL10 shown in green. **c**, Model. CIBB competes with CXCL10 to bind to IL18R1, thereby reversing the downstream signal induced by CXCL10. **d**,**e**, Immunofluorescence and quantification revealed that when oocytes were treated with recombinant CXCL10 only, some CXCL10 was internalized into the oocyte cytoplasm; however, when oocytes were co-treated with CXCL10 and CIBB, CXCL10 internalization significantly decreased. CXCL10 internalization is shown as the CXCL10 ratio (M/C, membrane CXCL10 intensity/cytoplasmic CXCL10 intensity). CXCL10 (red) was detected with anti-CXCL10 antibodies; CIBB (green) was detected with anti-Flag antibodies, and DNA is shown in blue; *N* = 5 for all groups, *P* = 0.0003. **f**,**g**, Ovarian weight significantly decreased in the M group, whereas CIBB treatment rescued the ovarian weight in the CI-M (CIBB plus MCA205 tumor) group. *N* = 4 for all groups, *P* = 0.0042. **h**,**i**, After tumor model period, at days 16, 19, 22, and 25, compared with control (CTR) (blue line), the anti-Müllerian hormone (AMH) level remains at significantly lower level in M group (red line), whereas the level remains at significantly higher level in CI-M group (green line). *N* = 5 for groups. **j**–**m**, Western blotting and quantification revealed that the levels of p-JNK, p-JUN, and COL1A1 significantly increased in the ovaries of the M group, whereas CIBB treatment significantly rescued their levels in the CI-M group. *N* = 4 for all groups. For p-JNK, CTR versusM, *P* = 0.0029; M versus CI-M, *P* = 0.0007. For p-JUN, CTR versus M, *P* = 0.0021; M versus CI-M, *P* = 0.0193. For COL1A1, CTR versus M, *P* = 0.0246; M versus CI-M, *P* = 0.0239. **n–p**, Western blotting and quantification revealed that the levels of p-AKT and p-FOXO3A significantly increased in the ovaries of the M group, whereas CIBB treatment significantly rescued their levels in the CI-M group. *N* = 4 for all groups. For p-AKT, CTR versus M, *P* = 0.0002; M versus CI-M, *P* = 0.0023. For p-FOXO3A, CTR versus M, *P* = 0.0055; M versus CI-M, *P* = 0.0015. **q**–**s**, Western blot and quantification revealed that the levels of p-P65 and interferon-γ (IFNγ) significantly increased in the ovaries of the M group, whereas CIBB treatment significantly rescued their levels in the CI-M group. *N* = 3 for all groups. For p-P65, CTR versus M, *P* = 0.003; M versus CI-M, *P* = 0.0006. For IFNγ, CTR versus M, *P* = 0.0354; M versus CI-M, *P* = 0.0157. **t**,**u**, Immunofluorescence and quantification revealed that the number of dots with inducible nitric oxide synthase (INOS), a marker of pro-inflammatory M1 macrophages, significantly increased in the M group, whereas CIBB treatment significantly rescued the number of dots in the CI-M group. *N* = 4 for all groups. CTR versus M, *P* = 0.002; M versus CI-M, *P* = 0.0017. INOS, green; 4′,6-diamidino-2-phenylindole (DAPI), blue. **v**,**w**, Immunofluorescence and quantification revealed that the number of dots with CD206, a marker of anti-inflammatory M2 macrophages, significantly decreased in the M group, whereas CIBB treatment significantly rescued the number of dots in the CI-M group. *N* = 4 for all groups. CTR versus M, *P* = 0.0427; M versus CI-M, *P* = 0.0171. CD206, green; DAPI, blue. **x**–**z**, Fluorescence-activated cell sorting and quantification revealed that, in the M group, the percentage of M1 macrophages significantly increased (37.76% in the M group and 32.30% in the CTR group). The percentage of M2 macrophages significantly decreased (18.81% in the M group and 28.24% in the CTR group). By contrast, CIBB treatment significantly reduced the percentage of M1 macrophages (31.81% in the CI-M group) while increasing the percentage of M2 macrophages (26.18% in the CI-M group) close to that of the control in the CI-M group. *N* = 4 for all groups. For M1 macrophages, CTR versus M, *P* = 0.0011; M versus CI-M, *P* = 0.0007. For M2 macrophages, CTR versus M, *P* = 0.0006; M versus CI-M, *P* = 0.0024. Scale bars in part **d**, 20 µm. Scale bars in parts **t** and **v**, 100 µm; scale bars in zoom, 20 µm. **a1**–**d1**, We started the tumor model with half number of cells (to make sure that tumor size does not exceed the ethical limitation at the end), did mating at the end of day 12 of tumor modeling, and then examined the embryo condition at DPC (day post-coitus) 7.5 between the corresponding three groups. Tumor progression in the M group significantly decreased both the number of implanted embryos and embryo weight. CIBB significantly rescued both indexes. For no. of implanted embryos, *N* = 5 for all groups; CTR versus M, *P* = 0.0002; M versus CI-M, *P* = 0.0013. For embryo weight, *N* = 49 for the CTR group; N = 11 for the M groups; *N* = 41 for the CI-M groups; CTR versus M, *P* < 0.0001; CTR versus CI-M, *P* < 0.0001; M versus CI-M, *P* < 0.0001. One-way analysis of variance is used for statistical comparisons between multiple (three or more) groups in the graphs, and different letters above the columns indicate significant differences. For western blot and fluorescence-activated cell sorting, the number of independent biological repeats (R) was the same as technical repeats (*N*); for all other experiments, the number of independent biological repeats (R) was 3. AU, arbitrary unit; NRTP, non-reproductive tumor progression.

The above results demonstrated that CXC10 antibody injection rescued against ovarian damage; however, it could also inhibit the normal function of CXCL10 outside of the ovaries^22^. Presumably, specifically inhibiting the binding between CXCL10 and IL18R1 could rescue ovarian damage without affecting its normal function in other tissues. We analyzed the interaction interface of CXCL10–IL18R1 with AlphaFold and selected the two models with the highest scores. In model 1, amino acids (AAs) 74–82 (highlighted in red) of CXCL10 (blue color) appeared to match AAs 245–306 (highlighted in green) of IL18R1 (gold color) closely; in model 2, AAs 45–67 (highlighted in purple) of CXCL10 (blue color) appeared to match AAs 243–297 (highlighted in green) of IL18R1 (gold color) closely. We fused the Flag sequence (DYKDDDDK) and cell-penetrating peptide sequence TAT (CYGRKKRRQRRR) to each of the upper CXCL10 regions and named them CIBB-1 (CXCL10–IL18R1 binding blocker 1) and CIBB-2 (CXCL10–IL18R1 binding blocker 2) (Fig. 10a,b and Supplementary Movies 1 and 2). We named the combination of these two sequences CIBB and expected that it could specifically block the binding between CXCL10 and IL18R1 (Fig. 10c).

We found that when in vitro-cultured oocytes were treated with recombinant CXCL10 only, some CXCL10 could be internalized into the oocyte cytoplasm; however, when the oocytes were co-treated with CXCL10 and CIBB, CXCL10 internalization significantly decreased, suggesting that CIBB effectively blocked the binding of CXCL10 to IL18R1 (Fig. 10d,e). Next, we found that CIBB treatment in the CI-M (MCA205 tumor + CIBB) group significantly reversed the MCA205 tumor-induced decrease in ovary weight (Fig. 10f,g). Notably, even after tumor model period, at days 16, 19, 22, and 25, compared with CTR (blue line), the AMH level remained at significantly lower level in the M group (red line), while at significantly higher level in the CI-M group (green line), suggesting that follicle reserve was improved for a long term (Fig. 10h,i).

Furthermore, we examined whether CIBB injection could rescue upper ovarian abnormalities caused by NRTP. First, CIBB treatment in the CI-M group reversed the MCA205 tumor-induced increase in p-JNK, p-JUN, and COL1A1 levels (Fig. 10j–m). Additionally, CIBB treatment in the CI-M group reversed the MCA205 tumor-induced increase in ovarian fibrosis (Supplementary Fig. 10a,b). Second, CIBB treatment in the CI-M group reversed the MCA205 tumor-induced increase in p-AKT and p-FOXO3A levels (Fig. 10n–p). Additionally, CIBB treatment in the CI-M group reversed the MCA205 tumor-induced increase in nuclear p-FOXO3A in PMFs (Supplementary Fig. 10c,d). Third, CIBB treatment in the CI-M group reversed the MCA205 tumor-induced increase in p-P65 and IFNγ levels (Fig. 10q–s). Moreover, CIBB treatment in the CI-M group reversed the MCA205 tumor-induced increase in pro-inflammatory M1 macrophages (Fig. 10t,u and Supplementary Fig. 15i) and decrease in anti-inflammatory M2 macrophages (Fig. 10v,w and Supplementary Fig. 15j). Finally, FACS and quantification (Fig. 10x–z) revealed that, in the M group, the percentage of M1 macrophages significantly increased (37.76% in the M group and 32.30% in the CTR group). The percentage of M2 macrophages significantly decreased (18.81% in the M group and 28.24% in the CTR group). By contrast, CIBB treatment in the CI-M group significantly reduced the percentage of M1 macrophages (31.81% in the CI-M group) and increased the percentage of M2 macrophages (26.18% in the CI-M group) close to that in the control group.

To exclude the possibility that IL18R1 binds as a result of the formation of the CXCL10–CXCR3 complex, we did comparative co-IP and quantification among CXCL10, CXCR3, and IL18R1. We found that as CXCL10 increased in the M group, IL18R1 could bait more CXCL10 than in Ctr ovary lysate and CXCL10 could also bait more IL18R1 than in Ctr ovary lysate; but CXCR3 could bait about the same amount of CXCL10 or IL18R1 in Ctr versus M group; and either CXCL10 or IL18R1 bait about the same amount of CXCR3 in Ctr versus M group. The upper result, plus the evidence that the ratio of IL18R1/CXCR3 is six fold more in the ovary than in the tumor, supports that IL18R1 is surplus and CXCL10 can directly bind to IL18R1 (Supplementary Fig. 11a–d).

To exclude the possibility that IL18R1 recruitment through signaling pathway activation is mediated by CXCL10 and CXCR# binding, we selected CXCR2 and CXCR4 that show higher binding affinity to CXCL10 than to other CXCR members through AlphaFold, and set up CTR versus CXCL10 versus CXCL10 + CXCR3 Ab (antibody) versus CXCL10 + CXCR2 Ab versus CXCL10 + CXCR4 Ab, and found that none of these three antibodies treatment rescues the increased level of p-JNK, P-AKT, and p-P65. These results suggested that CXCL10–IL18R1 activates the three downstream pathways independent of other CXCRs (Supplementary Fig. 11e–h).

To further verify the rescue outcome of CIBB, we started the tumor model with half number of cells (to make sure that tumor size does not exceed the ethical limitation at the end), did mating at the end of day 12 of tumor modeling, and then examined the embryo condition at DPC (day post-coitus) 7.5 between the corresponding three groups. We found that TP in the M group significantly decreased both the number of implanted embryos and embryo weight, CIBB significantly rescued both indexes (Fig. 10a1–d1).

These results further indicate that CXCL10 binding to IL18R1 is the key mechanism that induces ovarian damage and that CIBB can be used to specifically prevent CXCL10-induced ovarian damage caused by NRTP.

### CXCL10 might be a universal key factor that damages ovaries under diverse NRTPs

**Fig. 11:**
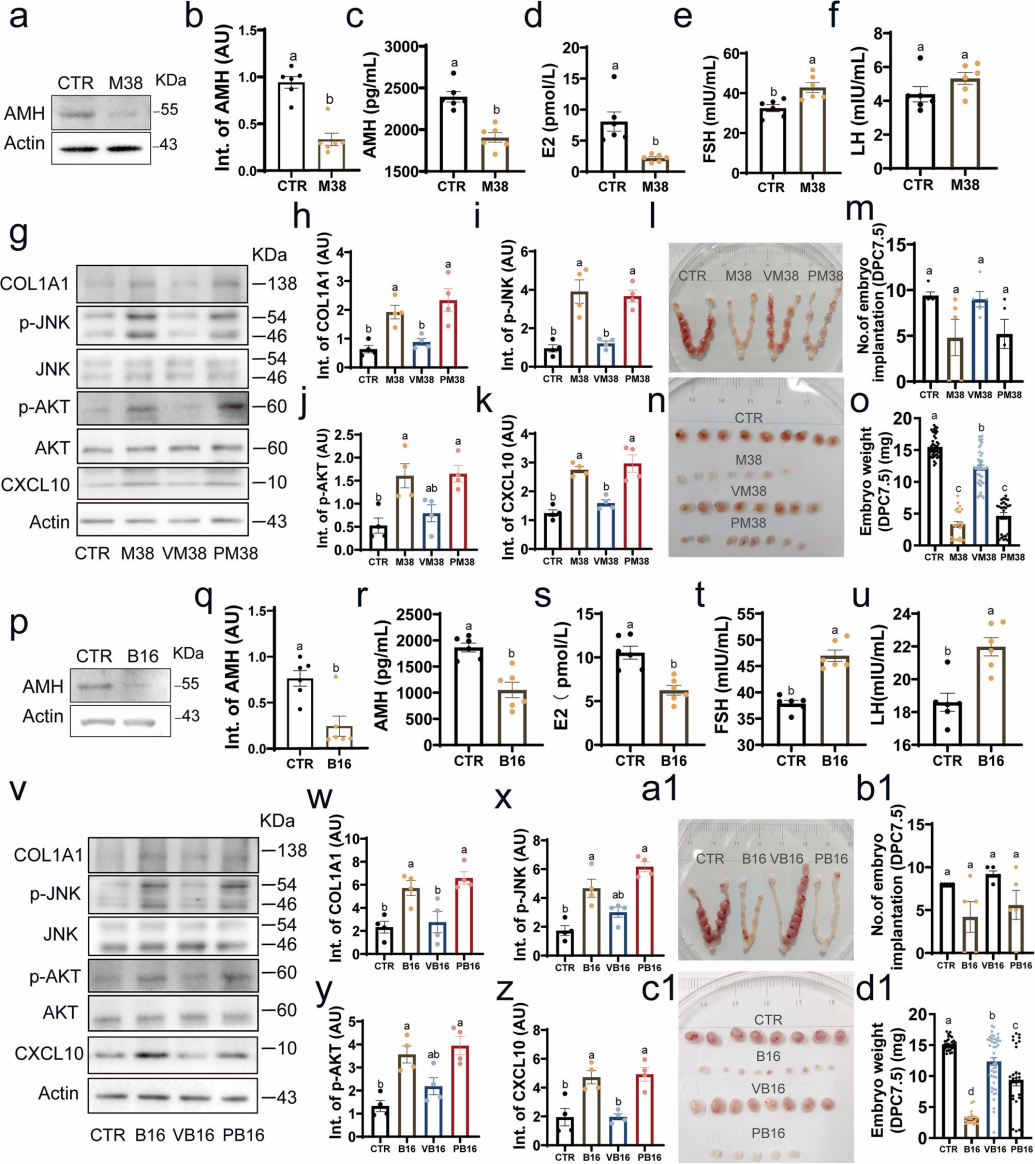
CXCL10 might be a universal key factor that damages ovaries under diverse NRTPs. **a**,**b**, Western blotting and quantification revealed that plasma anti-Müllerian hormone (AMH) level significantly decreased in M38 group. *N* = 6 for both groups, *P* < 0.0001. **c–f**. Enzyme-linked immunosorbent assay (ELISA) showed that AMH (*N* = 6, *P* = 0.0002) and E2 (*N* = 6, *P* = 0.0038) significantly decreased in the M38 group; follicle-stimulating hormone (FSH) (*N* = 6, *P* = 0.0067) significantly increased in the M38 group. **g–k**, Western blotting and quantification revealed that the ovarian levels of CXCL10, p-JNK, COL1A1, and p-AKT all significantly increased in M38 group, whole cancer cell vaccine (WCV) treatment significantly rescued their levels in the VM38 group, and PD-1 treatment had no obvious impact in the PM38 group. *N* = 4 for all groups. For COL1A1, control (CTR) versus M38, *P* = 0.0141; CTR versus PM38, *P* = 0.0018; M38 versus VM38, *P* = 0.0488; VM38 versus PM38, *P* = 0.0061. For p-JNK, CTR versus M38, *P* = 0.0004; CTR versus PM38, *P* = 0.0009; M38 versus VM38, *P* = 0.0009; VM38 versus PM38, *P* = 0.002. For p-AKT, CTR versus M38, *P* = 0.0121; CTR versus PM38, *P* = 0.0091; VM38 versus PM38, *P* = 0.0465. For CXCL10, CTR versus M38, *P* = 0.0004; CTR versus PM38, *P* = 0.0001; M versus VM38, *P* = 0.0032; VM versus PM38, *P* = 0.0008. **l–o**, We started the tumor model with half number of MC38 cells, did mating at the end of day 12 of tumor modeling, and then examined the embryo condition at DPC (day post-coitus) 7.5 among the corresponding three groups. Tumor progression (TP) in the M38 group significantly decreased both the number of implanted embryos and embryo weight, WCV treatment significantly rescued the number and size, and PD-1 antibody treatment did not show any effects. For the number of implanted embryos, *N* = 5 for all groups. For the embryo weight, *N* = 48 for the CTR group; *N* = 24 for the M38 groups; *N* = 46 for the VM38 groups; *N* = 25 for the PM38 groups; CTR versus M38, *P* < 0.0001; CTR versus VM38, *P* < 0.0001; CTR versus PM38, *P* < 0.0001; M38 versus VM38, *P* < 0.0001; VM38 versus PM38, *P* < 0.0001. **p**,**q**, Western blotting and quantification revealed that plasma AMH level significantly decreased in the B16 group. *N* = 6 for both groups, *P* = 0.0037. **r–u**, ELISA showed that AMH (*N* = 6, *P* = 0.0008) and E2 (*N* = 6, *P* = 0.0009) significantly decreased in the B16 group; FSH (*N* = 6, *P* < 0.0001) and luteinizing hormone (LH) (*N* = 6, *P* = 0.0016) significantly increased in the B16 group. **v–z**, Western blotting and quantification revealed that the ovarian levels of CXCL10, p-JNK, COL1A1, and p-AKT all significantly increased in the B16 group, WCV treatment significantly rescued their levels in the VB16 group, whereas PD-1 treatment had no obvious impact in the PB16 group. *N* = 4 for all groups. For COL1A1, CTR versus B16, *P* = 0.017; CTR versus PB16, *P* = 0.0035; B16 versus VB16, *P* = 0.0381; VB16 versus PB16, *P* = 0.0077. For p-JNK, CTR versus B16, *P* = 0.0021; CTR versus PB16, *P* < 0.0001; VB16 versus PB16, *P* = 0.0012. For p-AKT, CTR versus B16, *P* = 0.0032; CTR versus PB16, *P* = 0.0009; VB16 versus PB16, *P* = 0.0171. For CXCL10, CTR versus B16, *P* = 0.0053; CTR versus PB16, *P* = 0.0031; B16 versus VB16, *P* = 0.0056; VB16 versus PB16, *P* = 0.0033. **a1–d1**, We started the tumor model with half number of B16F10 cells, did mating at the end of day 12 of tumor modeling, and then examined the embryo condition at DPC 7.5 between the corresponding three groups. TP in the B16F10 group significantly decreased both the number of implanted embryos and embryo weight, WCV treatment significantly rescued the number and size, and PD-1 antibody treatment did not show any effect. For the number of implanted embryos, *N* = 5 for all groups; for the embryo weight, *N* = 32 for the CTR group; *N* = 21 for the B16 group; *N* = 46 for the VB16 groups; *N* = 28 for the PB16 group; CTR versus B16, *P* < 0.0001; CTR versus VB16, *P* = 0.0049; CTR versus PB16, *P* < 0.0001; B16 versus VB16, *P* < 0.0001; B16 versus PB16, *P* < 0.0001; VB16 versus PB16, *P* = 0.0043. Unpaired two-tailed *t*test is used for statistical comparison between two groups, one-way analysis of variance is used for statistical comparisons between multiple (three or more) groups in the graphs, and different letters above the columns indicate significant differences. For western blot and ELISA, the number of independent biological repeats (R) was the same as technical repeats (*N*); for all other experiments, the number of independent biological repeats (R) was 3. AU, arbitrary unit; NRTP, non-reproductive tumor progression.

To examine whether CXCL10 is still the key damaging factor on ovaries for the invasion of other NRTPs, we used another two cancer cell lines that are not related to female reproductive tissues: MC38, a mouse colon cancer cell line, and B16F10, a mouse melanoma cell line. We first showed that MC38 progression or B16F10 progression significantly decreased AMH level and altered the levels of several sex hormones (Fig. 11a–f for MC38 and Fig. 11p–u for B16F10). Similar to experiments with MCA205 cells, we set up four groups. For MCA38 model, the four groups are the CTR group, the M38 (MC38) group, the VM38 (WCV + MC38) group, and the PM38 (anti-PD-1 antibody + MC38) group; for B16F10, the four groups are the CTR group, the B16 (B16F10) group, the VB16 (WCV + B16F10) group, and the PB16 (anti-PD-1 antibody + B16F10) group. We first verified that both WCV and PD-1 treatment could significantly suppress MC38 or B16F10 tumor growth (Supplementary Fig. 12a,b for MC38 model and Supplementary Fig. 12e,f for B16F10 model). Next, we found that WCV but not PD-1 treatment significantly recovered ovary weight decrease under MCA38 or B16F10 TP (Supplementary Fig. 12c,d for MC38 model and Supplementary Fig. 12g,h for B16F10 model). These results suggested that these two NRTP models were successful, and WCV, instead of PD-1 treatment, could protect ovaries from NRTP-induced damage, which was similar to the upper results in the MCA205 model (Figs. 1 and 2).

Next, in parallel to the upper experiments (Fig. 3c–m), we examined the plasma level change of three cytokines with the highest plasma abundance, CXCL10, IL16, and CCL27, among four groups. Western blot showed that CXCL10 significantly increased in the plasma of M38 or B16 group, WCV treatment significantly reduced the CXCL10 level close to control, and PD-1 had no significant impacts (Supplementary Fig. 13a–d for MC38 model and Supplementary Fig. 13e–h for B16F10 model). Enzyme-linked immunosorbent assay had the similar results (Supplementary Fig. 13i–k for MC38 model and Supplementary Fig. 13l–n for B16F10 model). By contrast, plasma IL16 or CCL27 did not show a clear pattern between four groups.

Next, we examined whether we got similar results on ovaries for CXCL10 and its downstream signal pathways. We found that the levels of CXCL10 and its downstream effectors, p-JNK, COL1A1, and p-AKT, significantly increased in M38 or B16 group, WCV significantly reduced their levels close to control in VM38 or VB16 group, and PD-1 had no obvious impacts in PM38 or PB16 group (Fig. 11g–k for MC38 model and Fig. 11v–z for B16F10 model), which was also quite similar to the upper results in MCA205 model (Figs. Figs 6–8).

Next, we further verify the rescue outcome of WCV in these two types of tumor. Again, we started the tumor model with half number of cells, did mating at the end of day 12 of tumor modeling, and then examined the embryo condition at DPC 7.5 between the corresponding three groups. We found that TP in M38 or B16F10 group significantly decreased both the number of implanted embryos and embryo weight, WCV treatment significantly rescued the number and size, and PD-1 antibody treatment did not show any effects (Fig. 11l–o for MC38 model and Fig. 11a1–d1 for B16F10 model).

**Fig. 12:**
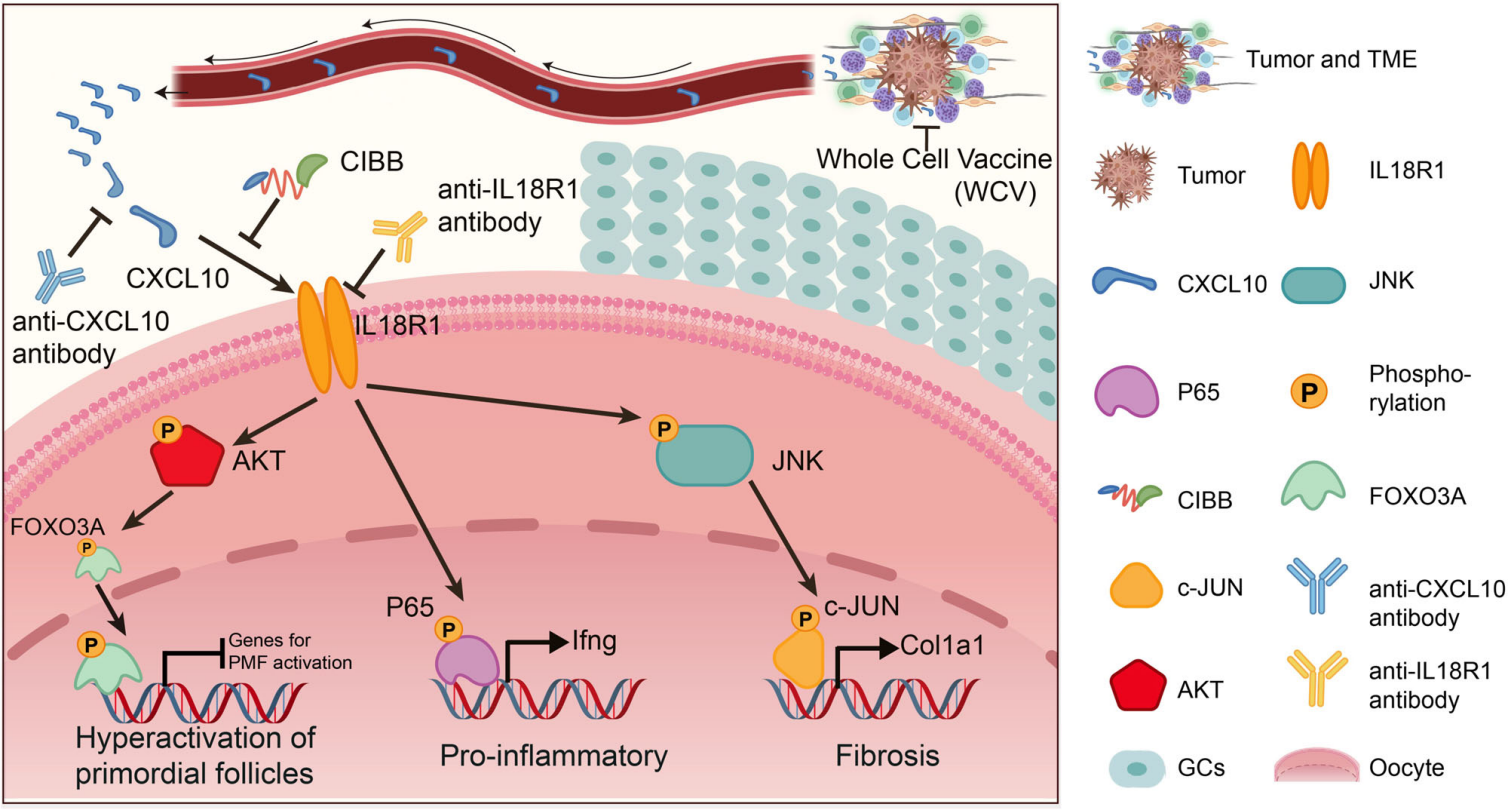
Model: blocking CXCL10–IL18R1 binding protects ovaries from NRTP-induced damage. Model: CXCL10 increases in blood circulation during NRTP and can be transported through the blood circulation to the ovaries. WCV treatment can suppress tumor growth and reduce the concentration of CXCL10. The anti-CXCL10 antibody can directly bind to CXCL10 to inhibit its function; similarly, the anti-IL18R1 antibody can directly bind to IL18R1 to inhibit CXCL10–IL18R1 binding. CIBB can compete with CXCL10 for binding to IL18R1, thus blocking the three major downstream signaling pathways of CXCL10–IL18R1: overactivation of primordial follicles (PMFs) caused by AKT and FOXO3A phosphorylation; elevated ovarian inflammation caused by the phosphorylation of P65, which promotes the transcription of IFNγ; and ovarian fibrosis caused by JNK and JUN phosphorylation, which promotes the transcription of COL1A1. GC, granulosa cell; NRTP, non-reproductive tumor progression; TME, tumor microenvironment.

Finally, we sought to examine whether the progression of reproductive tumors follows a similar mechanism. In the ID8 mouse ovarian epithelial cancer model, tumor-bearing mice exhibited a significant reduction in ovary weight. However, CIBB treatment in ID8 tumor-bearing mice (CI-ID8 group) failed to restore ovary weight (Supplementary Fig. 14a,b). Accordingly, CXCL10 levels showed no significant differences among the groups (Supplementary Fig. 14c,d). We further evaluated key downstream markers of the CXCL10–IL18R1 axis. Unlike the trends observed in the other three non-reproductive solid tumor models, most fibrosis-related (Supplementary Fig. 14e–h), follicle activation-related (Supplementary Fig. 14i–k), and inflammation-related markers (Supplementary Fig. 14l–n) did not show consistent alterations. Similarly, the levels of four key sex hormones — AMH, E2, FSH, and luteinizing hormone — also displayed distinct patterns compared with the other models (Supplementary Fig. 14o–r). These results suggest that ovarian damage caused by reproductive tumors may operate through a different mechanism, and the CXCL10–IL18R1 axis along with its three downstream signaling pathways appears to be specifically relevant to NRTPs (Fig. 12).

These results suggest that CXCL10 might also be the key detrimental factor on ovaries under the progression of various non-reproductive tumors.

## Discussion

To the best of our knowledge, this is the first mechanical study showing that NRTP itself impairs ovarian function in multiple ways through pro-inflammatory plasma cytokine binding to an ovarian receptor. By virtue of this specific mechanism, we further successfully developed a strategy of target-blocking this binding to protect the ovary from NRTP-induced damage.

First, we verified that in the plasma of female mice with fibrosarcoma invasion, a typical example of NRTP, CXCL10 was the primary pro-inflammatory cytokine that is detrimental to the ovaries. We demonstrated the damaging effects of CXCL10 both in vitro (CXCL10-treated ovaries cultured in vitro showed increased fibrosis, PMF overactivation, and increased inflammation) and in vivo (CXCL10 antibody injection very effectively rescued fibrosarcoma-induced ovarian damage in the upper three aspects). CXCL10 was previously shown to increase the efficacy of cancer immunotherapy^33^; from this point of view, it is a “beneficial” cytokine. However, in the ovaries, we found that it had multiple detrimental effects on the ovaries. These findings suggest that certain cytokines might exert positive or negative effects on different tissues, suggesting that tissue-specific targeted therapy is an essential strategy to protect other normal tissues, such as the ovary, from NRTP-induced damage.

Second, diagnosis of TP at its early stage is a primary goal most researchers and clinicians are pursuing. We showed that CXCL10 elevated the fastest at the early stage (day 3) of fibrosarcoma invasion. Furthermore, we have also preliminarily verified the key damaging impacts of CXCL10 on ovaries under the invasion of another two non-reproductive tumors (mouse colon cancer and mouse melanoma), making CXCL10 a potentially universal novel marker for the diagnosis of ovarian damages caused by NRTPs at the early stage.

Third, we characterized the primary receptor for CXCL10 in the ovaries, IL18R1, and showed that it dominates over CXCR3 in binding to CXCL10. We demonstrated the key transducing role of IL18R1 both in vitro (IL18R1 antibody treatment significantly reversed CXCL10-induced ovarian damage in in vitro-cultured ovaries) and in vivo (blocking CXCL10–IL18R1 binding could very effectively rescue fibrosarcoma-induced ovarian damage). Although IL18 is a common ligand for IL18R1, in this study, IL18R1 could also be bound by CXCL10. These findings suggest that different cytokines can bind to a specific receptor under different conditions.

Fourth, we designed a peptide, CIBB, that specifically and effectively blocked the interaction between CXCL10 and IL18R1. We showed that CIBB effectively protects ovaries from fibrosarcoma-induced damage. As CIBB is designed on the basis of the CXC10 regions that bind to IL18R1 with high affinity, CIBB blocks only the binding of CXCL10 to IL18R1 without affecting the positive role of CXCL10 in suppressing tumor growth. In comparison, anti-CXCL10 antibody might interfere with other regular tumor therapies as CXCL10 within tumor tissue binds CXCR3 to exert antitumor effect. Besides, CXCL10 antibody will be more costly than CIBB. Therefore, considering future clinical application, CIBB might be more advantageous than CXCL10 antibody as it can effectively protect NRTP-induced ovary damage while not interfering with other regular tumor therapies.

Fifth, we found that WCV treatment has several critical advantages over PD-1 checkpoint inhibitor therapy for improving female fertility, including reducing ovarian fibrosis, preserving the PMF reserve and oocyte quality, and reducing ovarian inflammation. In addition, we confirmed the functionality of WCV treatment in the context of NRTP. Loi  and Hutt reported that PD-L1 inhibitor treatment significantly impaired ovarian function, including oocyte number and quality^34^. Xu et al.^35^ reported that PD-1 blockade decreased primordial follicle reserves but did not affect short-term fertility. However, they did not examine the impact in the context of cancer invasion. Our study suggests that WCV might be an optimum immune therapy.

However, a key remaining question is how whole-cell vaccines effectively inhibit TP while also reversing tumor-induced damage. Regarding the former, unlike single-antigen vaccines such as peptide-based formulations, whole-cell vaccines retain the full antigenic repertoire of tumor cells, enabling simultaneous activation of polyclonal T cell responses against multiple antigens and thereby reducing the likelihood of immune escape. As for the latter, these vaccines can remodel the TME — including its immune and stromal components — leading to a reduction in pro-inflammatory cytokines such as CXCL10 (refs. ^36–38^). This is further supported by scRNA-seq results from our study, which show that many DEGs associated with immunity, cellular senescence, degenerative disease, or inflammation were significantly upregulated or downregulated in the model group but restored toward baseline in the WCV-treated group. By contrast, PD-1 antibodies primarily function by blocking PD-1/PD-L1 interaction and exhibit more limited effects on broad TME remodeling.

Another important question is what are the key factors that influence ovarian fibrosis, primary follicle overactivation, and inflammatory ovarian dysfunction. Do they crosstalk with each other? On the basis of our results, in the context of NRTP, CXCL10–IL18R1 emerges as a primary factor. Nevertheless, other factors likely bind to additional ovary-dominant receptors and contribute collectively to ovarian dysfunction. This notion is supported by the elevation of multiple cytokines in plasma (Fig. 3c) and the widespread changes in DEGs (Figs. 3a and 4a–h) observed in the tumor-bearing group. For example, NRTP also increased plasma CCL24. Cardiac-resident macrophages upregulate CCL24 during pressure overload-induced injury, and CCL24 binds to CCR3 to promote fibroblast proliferation via transforming growth factor-β activation^39^, suggesting a plausible role in ovarian fibrosis — although this has not yet been reported in the ovary. CCL24 also exacerbates airway eosinophilia and lung inflammation via MAP3K8-MEK1/2 signaling in house dust mite-challenged allergic mice^40^. In primary sclerosing cholangitis, CCL24 promotes both inflammation and fibrosis^41^. In Parkinson disease models, microglia drive neuroinflammation via JNK/AKT/NF-κB signaling^42^, and lipopolysaccharide activates inflammation in macrophages through the JNK/p-P65 pathway^43^. These observations collectively suggest that multiple cytokines likely contribute to the three major pathological features observed earlier and that these pathways likely crosstalk with each other.

Several scientific questions remain open. For instance, how is CXCL10 upregulation mediated? This may involve transcription factor activation or repressor inactivation triggered by NRTP-induced immune and metabolic remodeling. Although this lies beyond the scope of this study, it represents an important direction for future investigation.

In conclusion, this is the first study to show that NRTP can impair ovarian function through multiple pathways (fibrosis, PMF overactivation, and inflammation). In particular, fibrosarcoma, a typical tumor with a little-known relationship with the ovary, can increase plasma CXCL10, which reaches the ovaries, binds to IL18R1 on the ovaries, and triggers multiple downstream ovary-disrupting signals. CIBB could be used independently to protect ovaries from fibrosarcoma specifically (Fig. 12). We also preliminarily verified the key damaging impacts of CXCL10 and its downstream effectors on ovarian function under the invasion of another two non-reproductive tumors. This study provides a potentially universal novel strategy to protect ovaries from the damages of diverse NRTPs.

## Supplementary information

Supplementary Information

Supplementary dataset 1

Supplementary dataset 2

Supplementary dataset 3

Supplementary dataset 4

Supplementary movie 1

Supplementary movie 2

## Data Availability

The data that support the findings of this study are available from the corresponding author for scientific research upon reasonable request. Supplementary Datasets 1–4 have also been deposited into Zenodo, and the DOI is 10.5281/zenodo.19632127. The accession codes for the raw bulk RNA-seq (in Fig. 3a) and scRNA-seq data (in Fig. 4a–h) submitted to the NCBI Sequence Read Archive database were GSE248829 and PRJNA1456226, respectively; the accession code for the raw mass spectrometry data (in Fig. 5a) submitted to the ProteomeXchange Consortium was PXD047305.
