# Supplementary material for: Non-reproductive tumor progression impairs ovarian function through CXCL10–IL18R1 axis
Source: Exp Mol Med. 2026 Jul 22;58(7):2368–91. doi: 10.1038/s12276-026-01790-4 (PMC13434249; doi:10.1038/s12276-026-01790-4)
Supplement: Supplementary file 1 [file 12276_2026_1790_MOESM1_ESM.pdf]

Supplementary figures and figure legends

Supplementary Figure 1. Magnified whole ovaries in CTR and M group

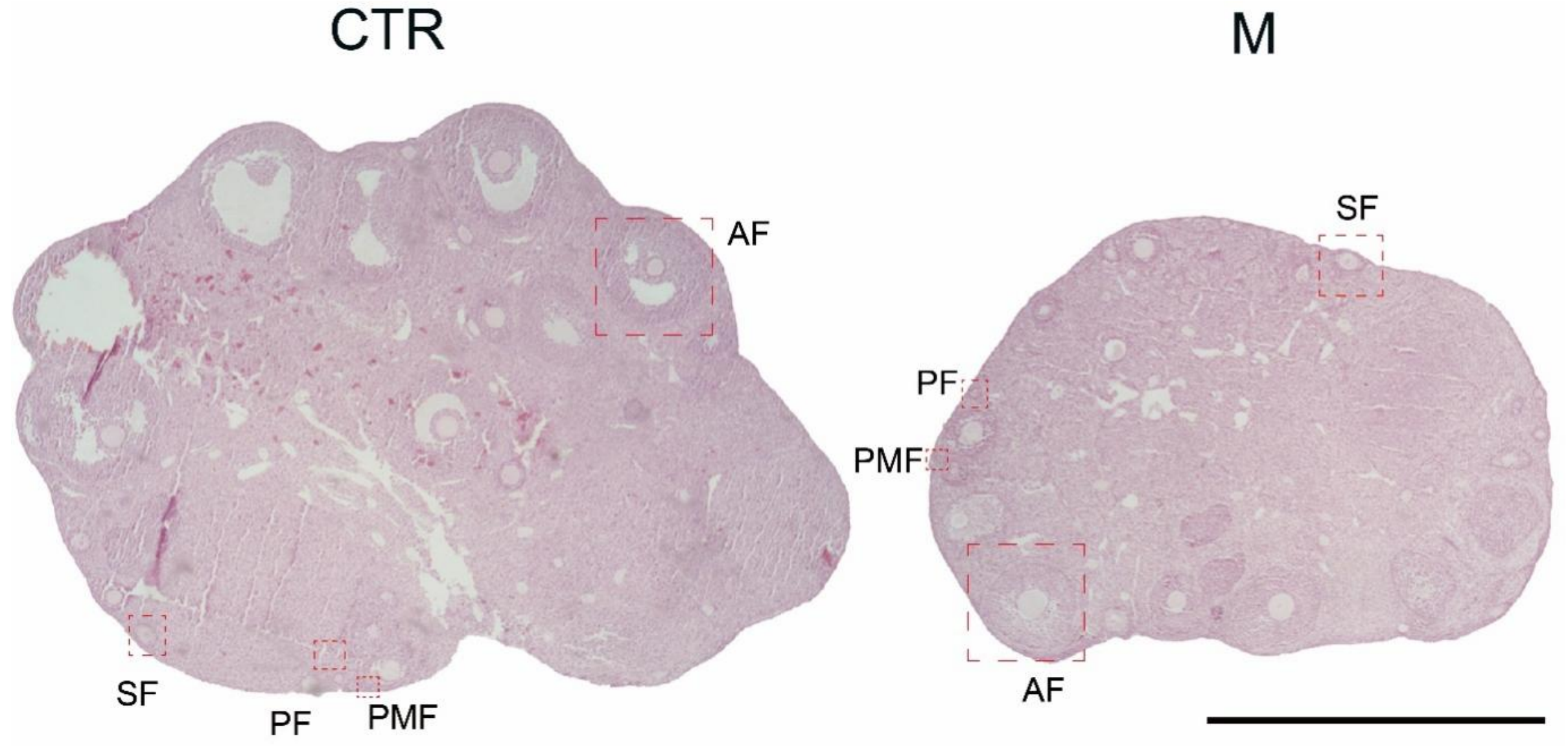

These two ovaries are the same as those in Fig. 1a. The images here are magnified to show the follicle structure more clearly. Typical PMF, PF, SF, and AF are dot-line square-labeled.

Supplementary Figure 2. Heatmaps of all repeats from RNA-seq and multiplex immunoassay showed that CXCL10 might be the key pro-inflammatory cytokine under NRTP

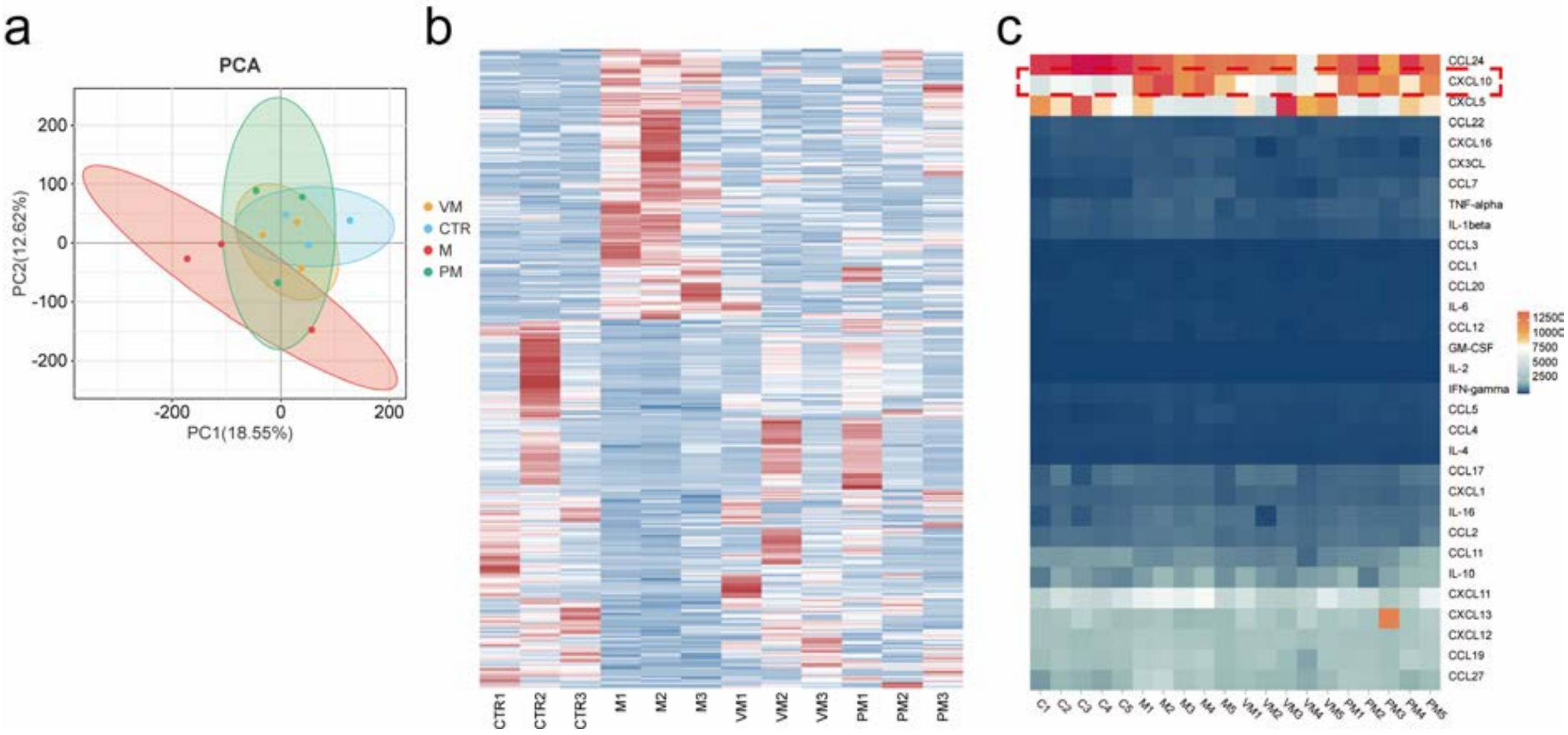

a. PCA analysis showed that the cluster of all 4 repeats in M group almost completely separates from CTR group, the cluster of VM group has the largest overlap with CTR group, while the cluster of PM group has some overlap with both M and CTR groups. **b.** The RNA sequencing heatmap of all three repeats per group revealed 435 DEGs (differentially expressed genes) between the CTR group and the M group at the threshold of |log2(M/CTR)| ≥ 1. The expression profile in the VM group was similar to that in the CTR group, whereas the expression profile in the PM group was only somewhat close to that in the CTR group. **c.** Heatmaps of all five replicates per group in the multiplex immunoassay. CXCL10 was the most highly upregulated gene in the M group (red dotted rectangle) and was restored close to the control level in the VM group; no significant rescue was observed in the PM group.

Supplementary Figure 3. Comparison of all cytokine levels in the multiplex immunoassay showed that CXCL10 might be the key pro-inflammatory cytokine under NRTI

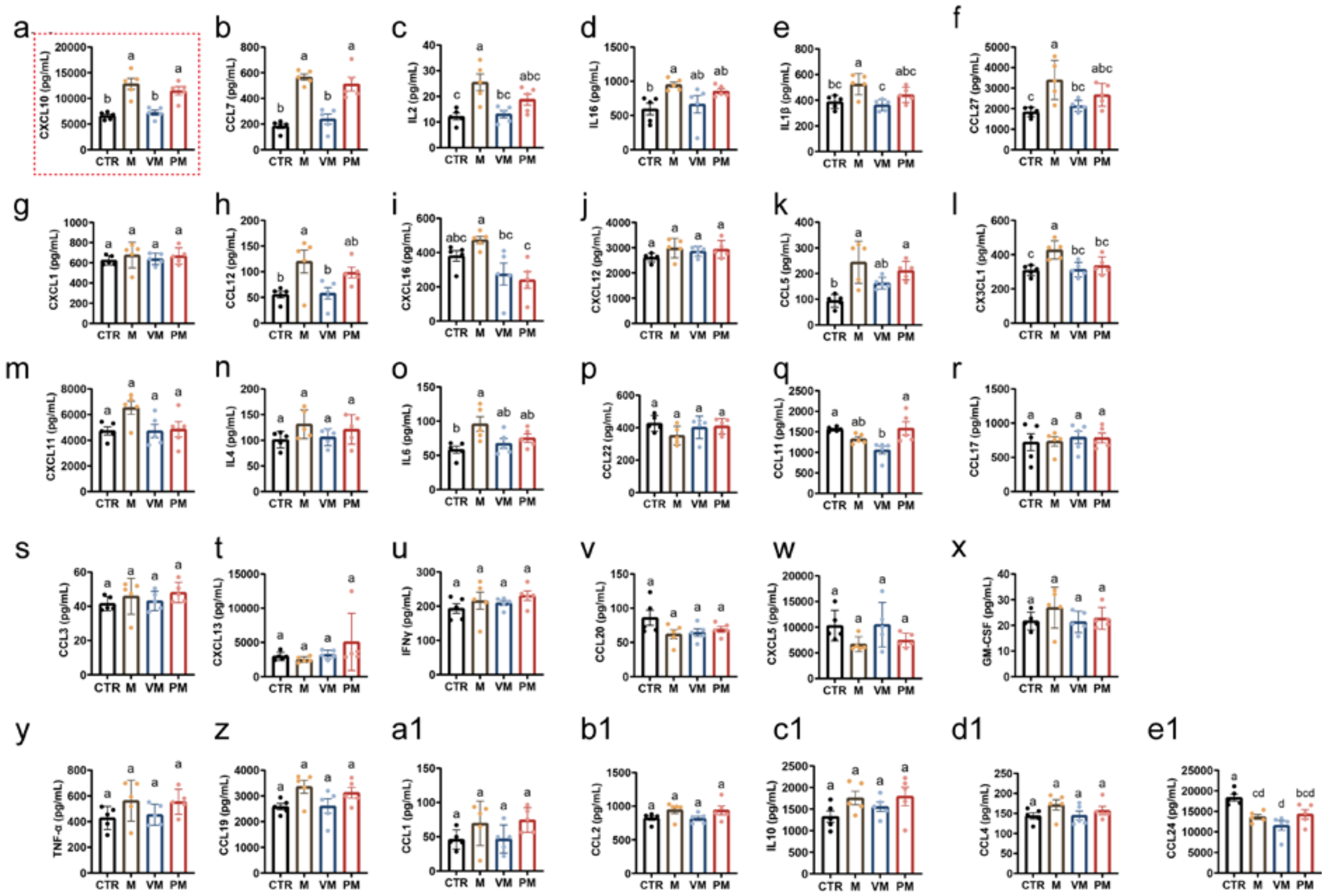

a. For CXCL10, N = 5 for all groups; CTR *vs*. M, *P* < 0.0001; CTR *vs*. PM, *P* = 0.0012; M *vs*. VM, *P* = 0.0003; VM *vs*. PM, *P* = 0.0042. CXCL10 was the most highly upregulated gene in the M group (red dotted rectangle), which was effectively rescued in the VM group. **b.** For CCL7, N = 5 for all groups; CTR *vs*. M, *P* < 0.0001; CTR *vs*. PM, *P* < 0.0001; M *vs*. VM, *P* < 0.0001; VM *vs*. PM, *P* = 0.0004. **c.** For IL2, N = 5 for all groups; CTR *vs*. M, *P* = 0.0027; M *vs*. VM, *P* = 0.005. **d.** For IL16, N = 5 for all groups; CTR *vs*. M, *P* = 0.0316. **e.** For IL-1β, N = 5 for all groups; CTR *vs*. M, *P* = 0.0122; M *vs*. VM, *P* = 0.0034. **f.** For CCL27, N = 5 for all groups; CTR *vs*. M, *P* = 0.003; M *vs*. VM, *P* = 0.0159. **g.** For CXCL1, N = 5 for all groups. **h.** For CCL12, N = 5 for all groups; CTR *vs*. M, *P* = 0.0215; M *vs*. VM, *P* = 0.0277. **i.** For CXCL16, N = 5 for all groups; M *vs*. VM, *P* = 0.0271; M *vs*. PM, *P* = 0.0087. **j.** For CXCL12, N = 5 for all groups. **k.** For CCL5, N = 5 for all groups; CTR *vs*. M, *P* < 0.0001; CTR *vs*. PM,

P = 0.0012. **l.** For CX3CL1, N = 5 for all groups; CTR *vs*. M, *P* = 0.0037; M *vs*. VM, *P* = 0.0052; VM *vs*. PM, *P* = 0.025. **m.** For CXCL11, N = 5 for all groups. **n.** For IL4, N = 5 for all groups. **o.** For IL6, N = 5 for all groups; CTR *vs*. M, *P* = 0.0144. **p.** For CCL22, N = 5 for all groups. **q.** Forf CCL11, N = 5 for all groups; CTR *vs*. VM, *P* = 0.0097; VM *vs*. PM, *P* = 0.0069. **r.** For CCL17, N = 5 for all groups. **s.** For CCL3, N = 5 for all groups. **t.** For CXCL13, N = 5 for all groups. **u.** For IFNγ, N = 5 for all groups. **v.** For CCL20, N = 5 for all groups. **w.** For CXCL5, N = 5 for all groups. **x.** For GM-CSF, N = 5 for all groups. **y.** For TNF-α, N = 5 for all groups. **z.** For CCL19, N = 5 for all groups. **a1.** For CCL1, N = 5 for all groups. **b1.** For CCL2, N = 5 for all groups. **c1.** For IL10, N = 5 for all groups. **d1.** For CCL4, N = 5 for all groups. **e1.** For CCL24, N = 5 for all groups; CTR *vs*. M, *P* = 0.0154; CTR *vs*. VM, *P* = 0.0007; CTR *vs*. PM, *P* = 0.0395. One-way ANOVA is used for statistical comparisons between multiple (3 or more) groups in the graphs, different letters above the columns indicate significant differences. The number of independent biological repeats (R) was the same as technical repeats (N).

Supplementary Figure 4. Comparison of fibrosis under NRTI or immunotherapy

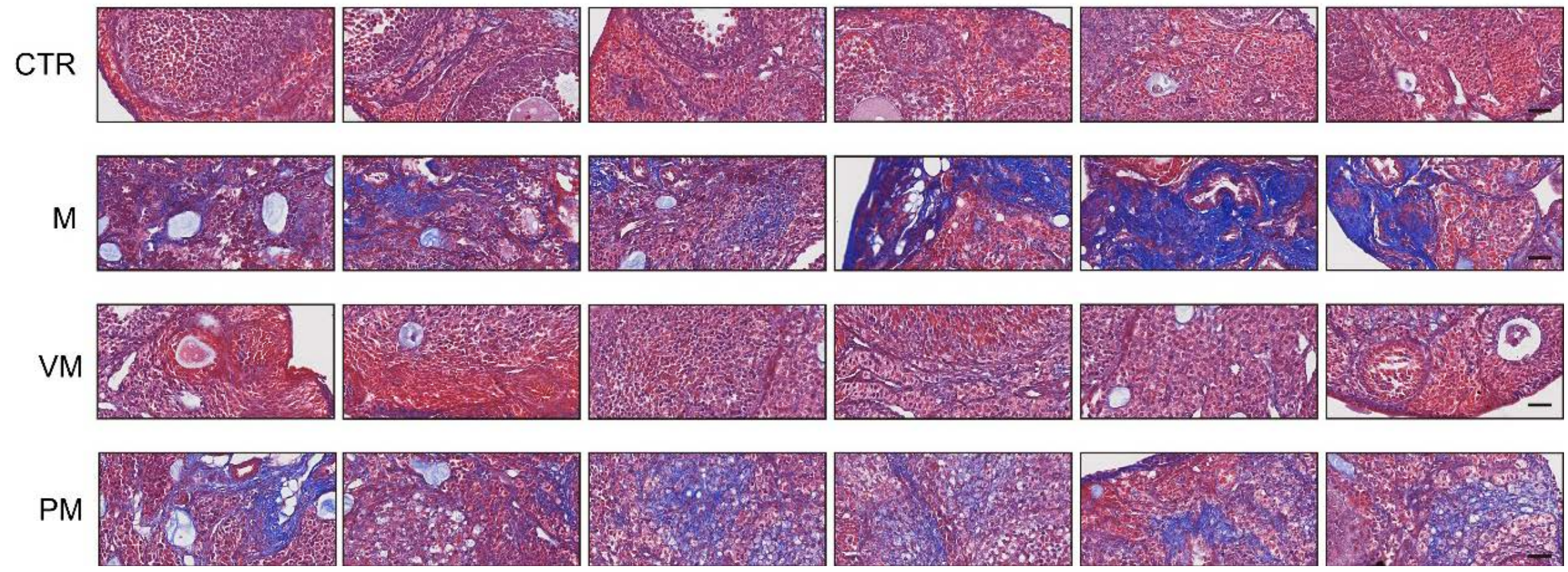

This figure corresponds to Fig. 6a and shows images of all six replicates per group. Scale bar, 50 µm. The number of independent biological repeats (R) was 3.

Supplementary Figure 5. P-c-JUN inhibitor can rescue CXCL10-induced downstream COL1A1 increment in IVC ovaries

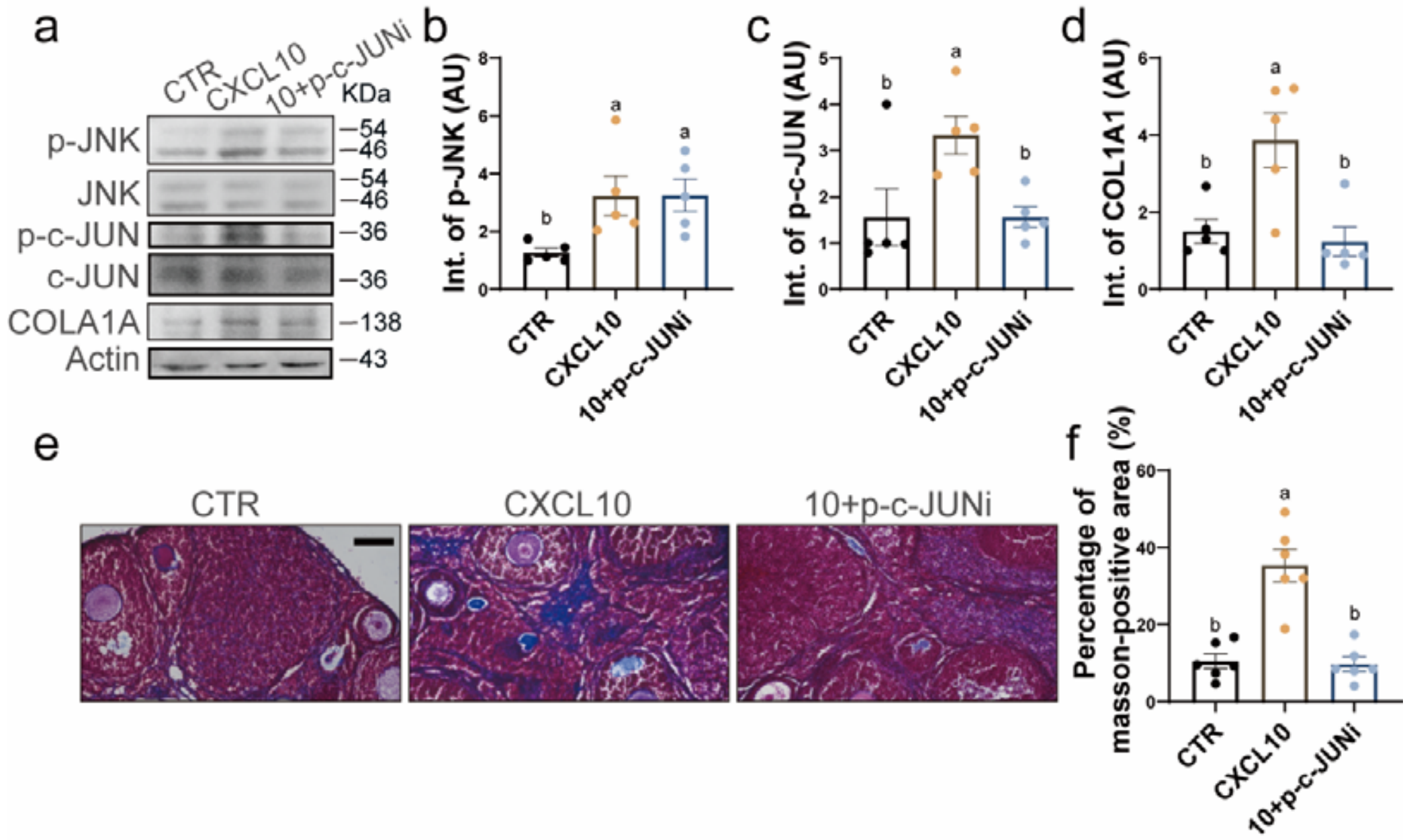

The group setting is CTR *vs*. CXCL10 *vs*. 10+P-c-JUNi for all panels. **a-d**. COL1A1 level significantly increased in CXCL10-treated in-vitro-cultured (IVC) ovaries, p-c-JUN inhibitor (p-c-JUNi) treatment significantly rescued CXCL10-induced COL1A1 upregulation. The specificity and effectiveness of p-c-JUNi was verified by the unchanged p-JNK and decreased p-c-JUN itself. N = 5 for all blots. For p-JNK, CTR *vs*. CXCL10, $P = 0.0492$; CTR *vs*. 10+p-c-JUNi, $P = 0.0461$. For p-c-JUN, CTR *vs*. CXCL10, $P = 0.0378$; CXCL10 *vs*. 10+p-c-JUNi, $P = 0.0384$. For COL1A1, CTR *vs*. CXCL10, $P = 0.0145$; CXCL10 *vs*. 10+p-c-JUNi, $P = 0.0072$. **e and f**. Masson staining showed that fibrosis level significantly increased in CXCL10-treated IVC ovaries; p-c-JUNi significantly rescued CXCL10-induced fibrosis increase. N = 6 for all groups. CTR *vs*. CXCL10, $P < 0.0001$; CXCL10 *vs*. 10+ p-c-JUNi, $P < 0.0001$. Scale bar, 50 µm. One-way ANOVA is used for statistical comparisons between multiple (3 or more) groups in the graphs, different letters above the columns indicate significant differences. For western blot, the number of independent biological repeats (R) was the same as technical repeats (N); for masson staining, the number of independent biological repeats (R) was 3.

Supplementary Figure 6. P-AKT inhibitor can rescue CXCL10-induced downstream p-FOXO3A increment in IVC ovaries

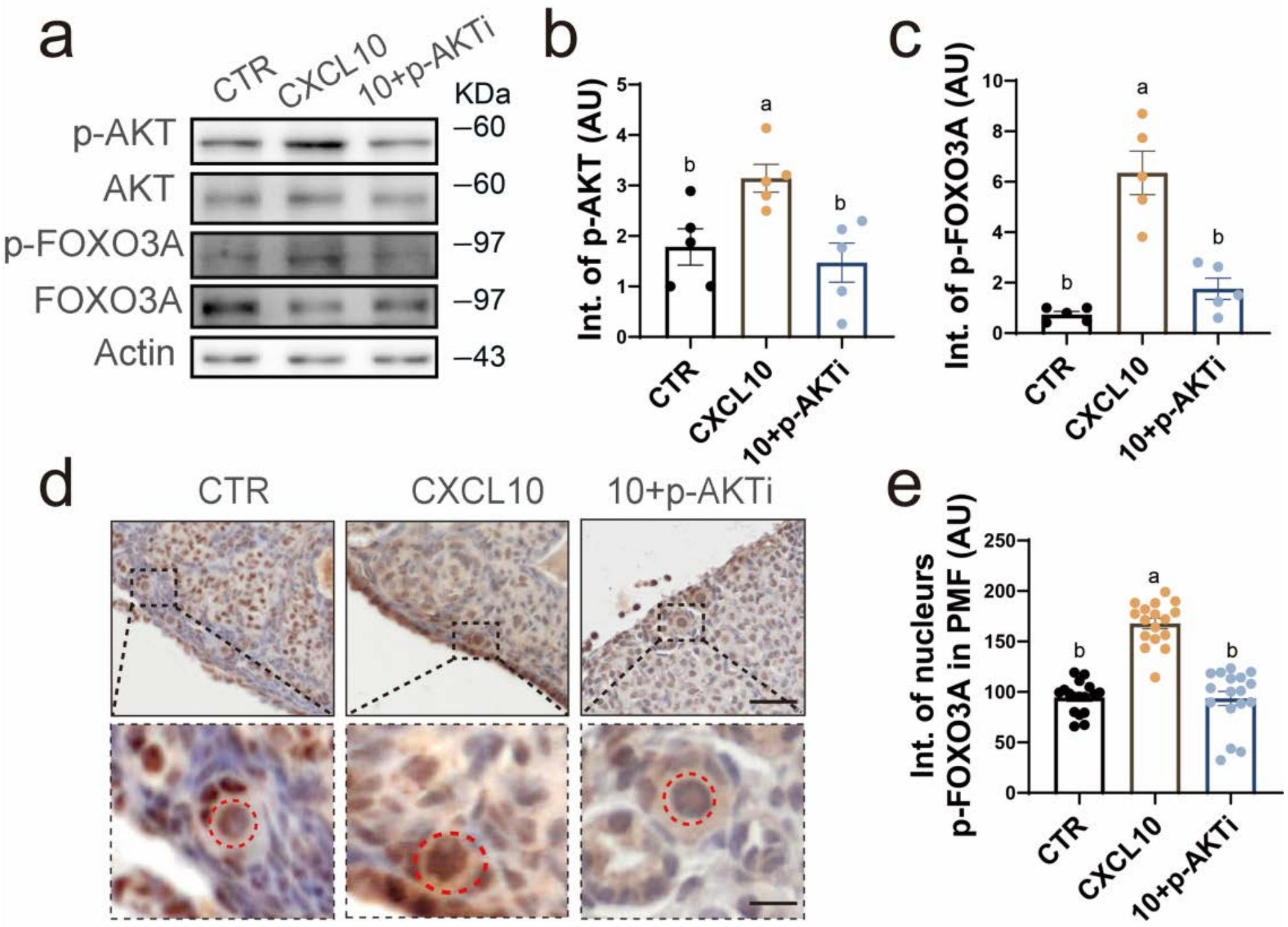

The group setting is CTR *vs*. CXCL10 *vs*. 10+p-AKTi for all panels. **a-c**. P-FOXO3A level significantly increased in CXCL0-treated IVC ovaries, p-AKT inhibitor (p-AKTi) treatment significantly rescued CXCL10-induced p-FOXO3A upregulation. The effectiveness of p-AKTi was verified by the decreased p-AKT itself. N = 5 for all blots. For p-AKT, CTR *vs*. CXCL10, *P* = 0.0406; CXCL10 *vs*. 10+p-AKTi, *P* = 0.0129; For p-FOXO3A**,** CTR *vs*. CXCL10, *P* < 0.0001; CXCL10 *vs*. 10+p-AKTi, *P* = 0.0002. **d and e**. P-FOXO3A immunohistochemistry (IHC) showed that nucleus p-FOXO3A level significantly increased in CXCL10-treated IVC ovaries; p-AKTi significantly rescued CXCL10-induced nucleus p-FOXO3A increase. N = 17 for all groups. CTR *vs*. CXCL10, *P* < 0.0001; CXCL10 *vs*. 10+ 10+p-AKTi, *P* < 0.0001. Scale bar in d, 100 μm, scale bar in Zoom, 20 μm. One-way ANOVA is used for statistical comparisons between multiple (3 or more) groups in the graphs, different letters above the columns indicate significant differences. For western blot, the number of independent biological repeats (R) was the same as technical repeats (N); for FOXO3A staining, the number of independent biological repeats (R) was 3.

Supplementary Figure 7. Flow cytometry gating strategy for ovarian macrophages

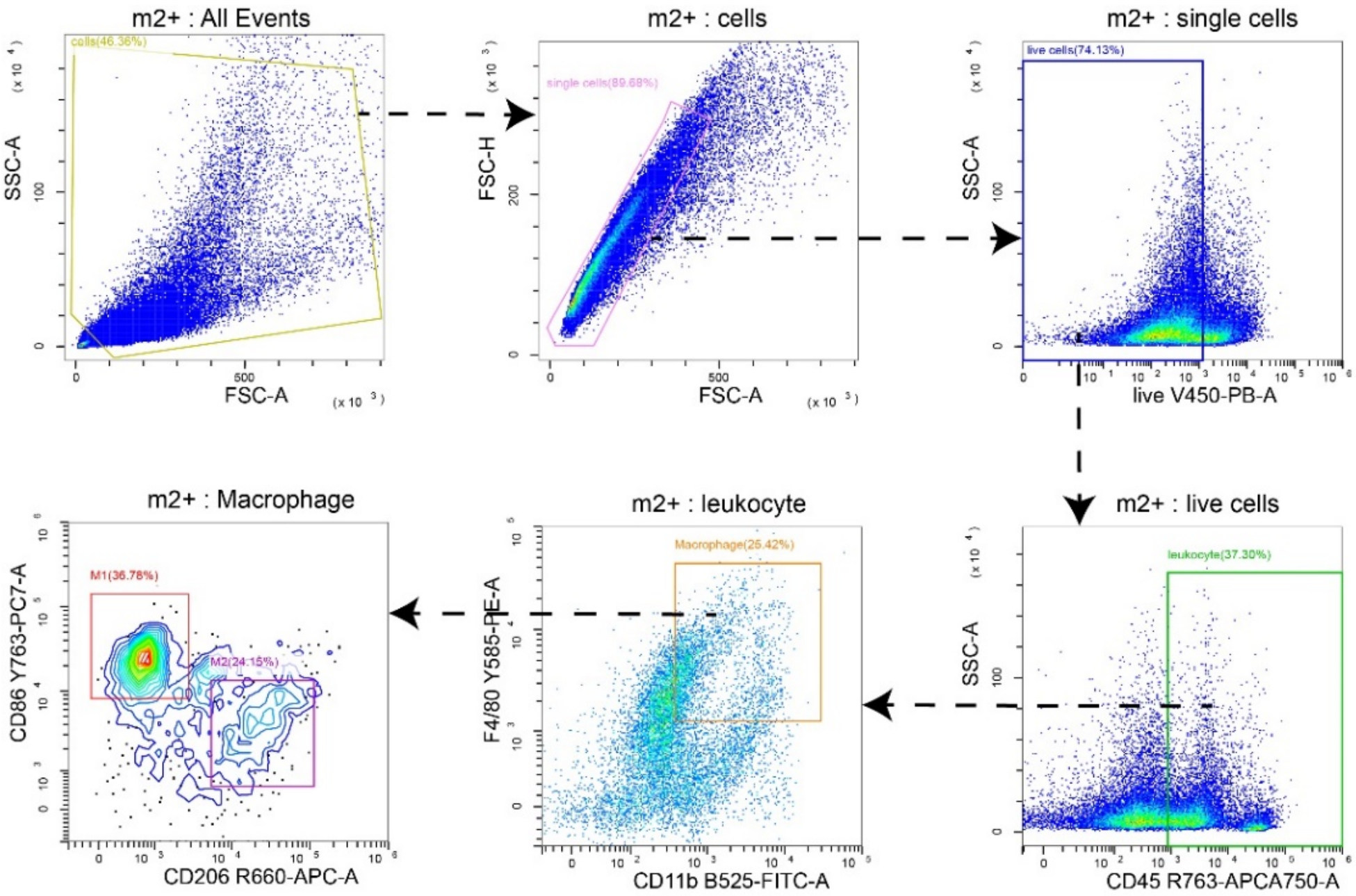

The gating strategy was used to identify ovarian macrophage populations in control mice and tumor-bearing mice with or without immunotherapy treatment. Note that the dotted-line arrows indicate the steps in order.

Supplementary Figure 8. P-P65 inhibitor can rescue CXCL10-induced downstream IFNγ increment in IVC ovaries

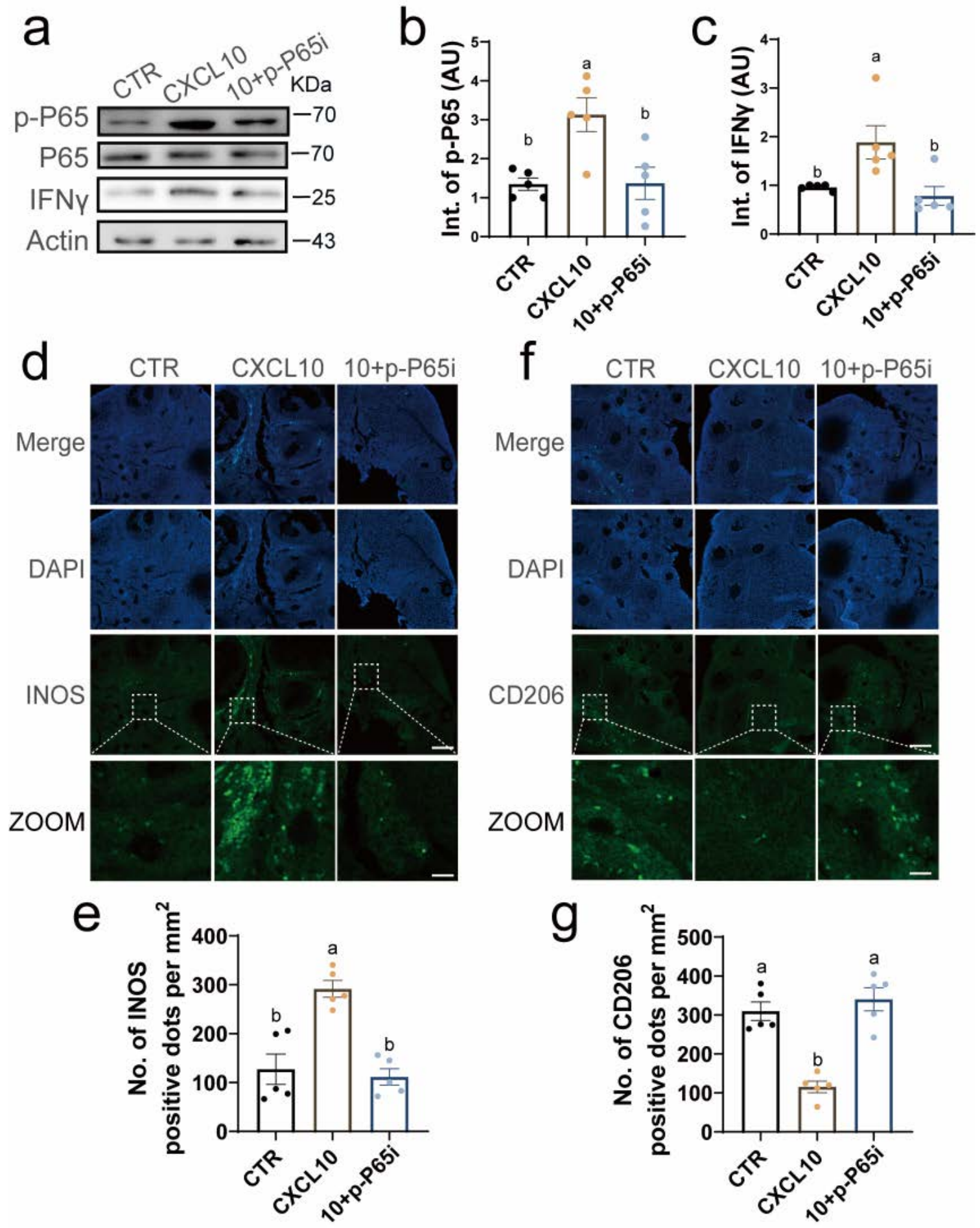

The group setting is CTR *vs*. CXCL10 *vs*. 10+p-P65i for all panels. **a-c**. IFNγ level significantly increased in CXCL0-treated IVC ovaries; p-P65 inhibitor (p-P65i) treatment significantly rescued CXCL10-induced IFNγ upregulation. The effectiveness of p-P65i was verified by the significantly decreased p-P65 itself. N = 5 for all blots. For p-P65, CTR *vs*. CXCL10, *P* = 0.0105; CXCL10 *vs*. 10+p-P65i, *P* = 0.0114; For IFNγ**,** CTR *vs*. CXCL10, *P* = 0.0334; CXCL10 *vs*. 10+p-P65i, *P* = 0.0124. **d and e**. Ovary immunostaining showed that the pro-inflammatory M1 macrophage, as indicated by INOS positive dots, significantly increased in CXCL10-treated IVC ovaries; p-P65i significantly rescued CXCL10-induced M1 macrophage increment. N = 5 for all blots. CTR *vs*. CXCL10, *P* = 0.0007; CXCL10 *vs*. 10+p-P65i, *P* = 0.0003. **f and g**. Ovary immunostaining showed that the anti-inflammatory M2 macrophage, as

indicated by CD206 positive dots, significantly decreased in CXCL10-treated IVC ovaries; p-P65i significantly rescued CXCL10-induced M2 macrophage increment. N = 5 for all groups. CTR *vs*. CXCL10, $P = 0.0002$; CXCL10 *vs*. 10+p-P65i, $P < 0.0001$. Scale bars in d and f, 100 μm; scale bars in zoom, 20 μm. One-way ANOVA is used for statistical comparisons between multiple (3 or more) groups in the graphs, different letters above the columns indicate significant differences. For western blot, the number of independent biological repeats (R) was the same as technical repeats (N); for immunostaining experiments, the number of independent biological repeats (R) was 3.

Supplementary Figure 9. CXCL10 antibody injection rescued the NRTP-induced increase in fibrosis and nuclear p-FOXO3A within ovaries.

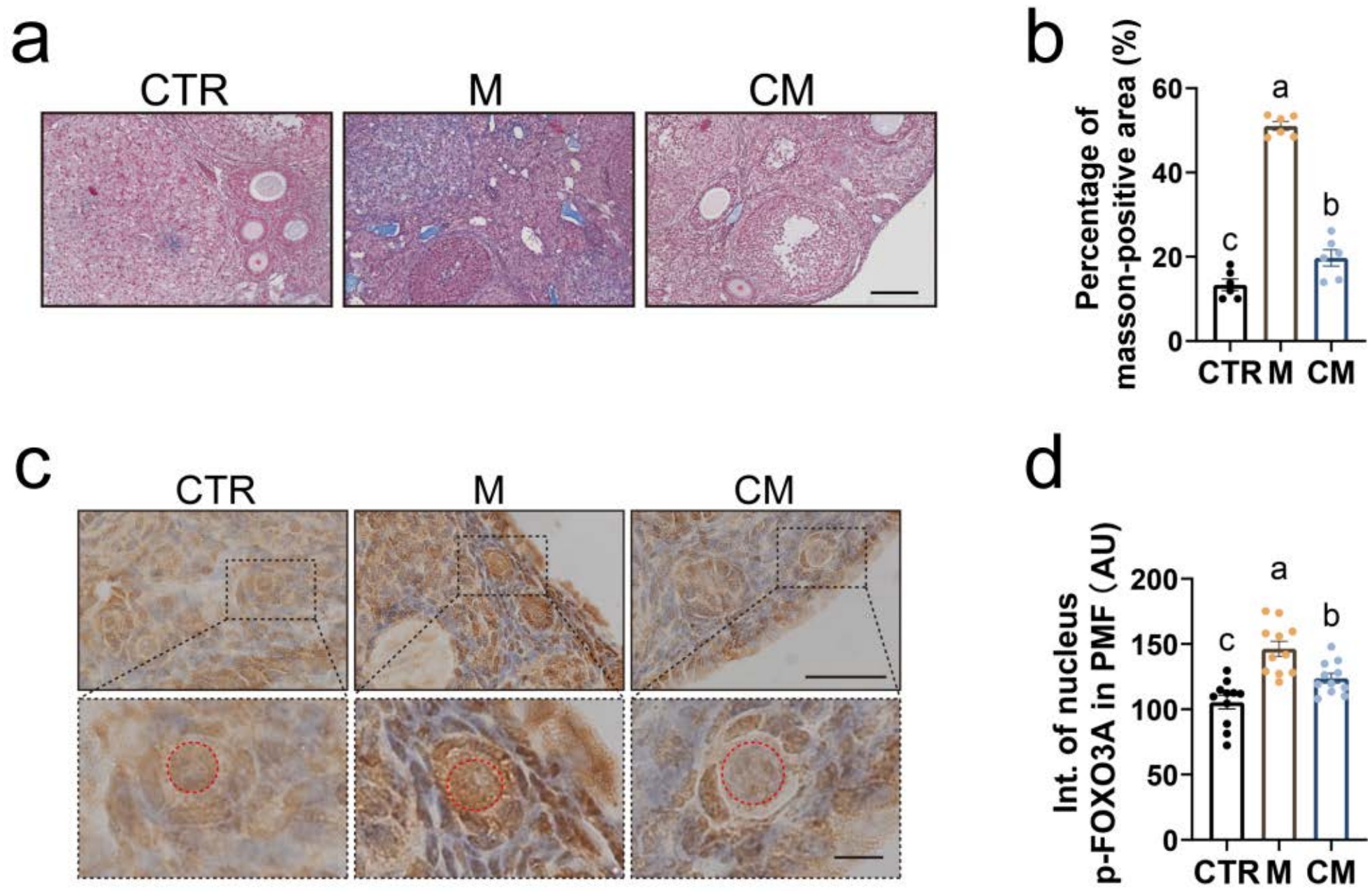

a and **b**. Masson staining and quantification revealed that fibrosis level significantly increased in the ovaries of the M group; anti-CXCL10 antibody treatment significantly rescued the fibrosis level in the CM group. N = 6 for all groups. CTR *vs*. M, $P < 0.0001$; CTR *vs*. CM, $P = 0.0224$; M *vs*. CM, $P < 0.0001$. **c** and **d**. p-FOXO3A immunohistochemistry of ovarian paraffin sections and quantification revealed that nuclear p-FOXO3A intensity (red dotted-line circle) within PMFs significantly increased in the M group, whereas anti-CXCL10 antibody treatment significantly rescued the nucleus p-FOXO3A intensity in the CM group; N = 11 for all groups. CTR *vs*. M, $P < 0.0001$; CTR *vs*. CM, $P = 0.0378$; M *vs*. CM, $P = 0.0093$. Scale bars in a, 200 μm. Scale bars in c, 100 μm; scale bars in Zoom, 20 μm. One-way ANOVA is used for statistical comparisons between multiple (3 or more) groups in the graphs, different letters above the columns indicate significant differences. For all experiments, the number of independent biological repeats (R) was 3.

Supplementary Figure 10. CIBB injection rescued the NRTI-induced increase in fibrosis and nuclear p-FOXO3A levels within ovaries.

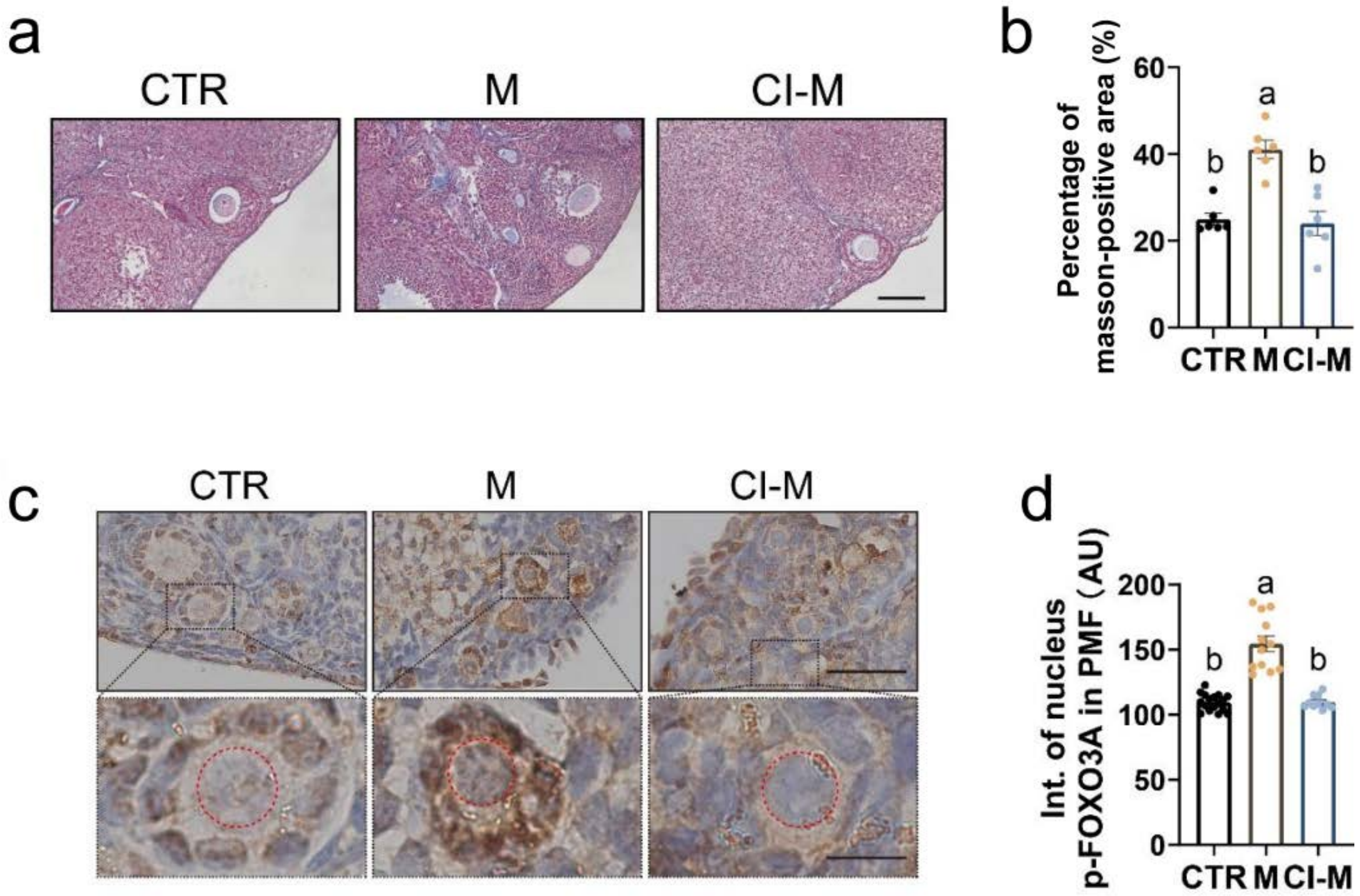

a and **b**. Masson staining and quantification revealed that fibrosis significantly increased in the ovaries of the M group, whereas CIBB treatment significantly rescued the fibrosis level in the CI-M group; N = 6 for all groups. CTR *vs*. M, $P = 0.0003$; M *vs*. CI-M, $P = 0.0002$. **c** and **d**. p-FOXO3A immunohistochemistry of ovarian paraffin sections and quantification revealed that the nucleus p-FOXO3A intensity (red dotted-line circle) in PMFs significantly increased in the M group, whereas CIBB treatment significantly rescued the p-FOXO3A intensity in the CI-M group; n = 17, 12, and 10 for the CTR, M, and CI-M groups; CTR *vs*. M, $P < 0.0001$; M *vs*. CI-M, $P < 0.0001$. Scale bars in a, 200 μm. Scale bars in c, 100 μm; scale bars in Zoom, 25 μm. One-way ANOVA is used for statistical comparisons between multiple (3 or more) groups in the graphs, different letters above the columns indicate significant differences. For all experiments, the number of independent biological repeats (R) was 3.

Supplementary Figure 11. CXCL10 induces downstream pathways through directly binding to the dominant receptor IL18R1 in ovaries.

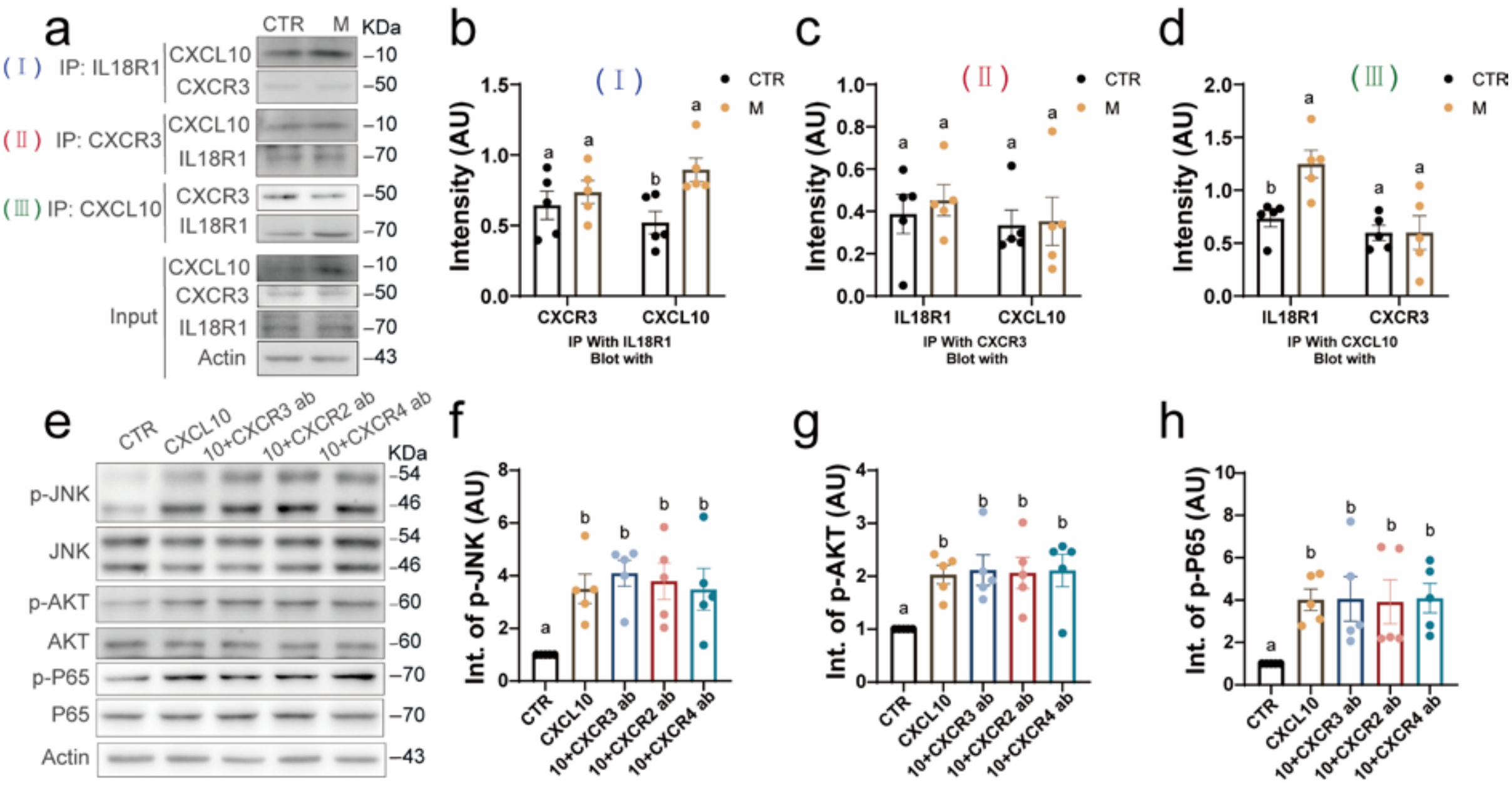

a-d. Comparative co-IP and quantification between CXCL10, CXCR3, and IL18R1. We found that as CXCL10 increased in M group, IL18R1 could bait more CXCL10 than in CTR ovary lysate (a and b), CXCL10 could also bait more IL18R1 than in Ctr ovary lysate (a and c); however, CXCR3, bait about the same amount of CXCL10 or IL18R1 in Ctr *vs*. M group (a and d); and either IL18R1 (a and b) or CXCL10 (a and c) bait about the same amount of CXCR3 in Ctr *vs*. M group. N = 5 for all groups. For IL18R1-baited CXCL10, CTR *vs*. M, *P* = 0.0119; For CXCL10-baited IL18R1, CTR *vs*. M, *P* = 0.0092. **e-h**. In comparison with IL18R1 antibody, we compared the rescuing effects of the antibodies for other three CXCR family members, CXCR3, CXCR2, and CXCR4, which are known or predicted to bind CXCL10. Results showed that none of the three antibodies could rescue the effects of CXCL10 treatment on p-JNK, p-AKT, and p-P65 in IVC ovaries. N = 5 for all groups. For p-JNK, CTR *vs*. CXCL10, *P* = 0.0422; CTR *vs*. CXCL10+CXCR3 ab, *P* = 0.0088; CTR *vs*. CXCL10+CXCR2 ab, *P* = 0.0202; CTR *vs*. CXCL10+CXCR4 ab, *P* = 0.0443. For p-AKT, CTR *vs*. CXCL10, *P* = 0.0483; CTR *vs*. CXCL10+CXCR3 ab, *P* = 0.0286; CTR *vs*. CXCL10+CXCR2 ab, *P* = 0.0397; CTR *vs*. CXCL10+CXCR4 ab, *P* = 0.0298. For p-P65, CTR *vs*. CXCL10, *P* =

0.0378; CTR *vs*. CXCL10+CXCR3 ab, *P* = 0.0348; CTR *vs*. CXCL10+CXCR2 ab, *P* = 0.046; CTR *vs*. CXCL10+CXCR4 ab, *P* = 0.0327. Unpaired two-tailed *t*-test is used for statistical comparison between two groups, one-way ANOVA is used for comparisons between multiple (3 or more) groups in the graphs, different letters above the columns indicate significant differences. For all western blot and co-IP experiments, the number of independent biological repeats (R) was the same as technical repeats (N).

Supplementary Figure 12. WCV treatment instead of PD-1 treatment significantly recovered reduced ovarian weight under MC38 or B16F10 tumor invasion

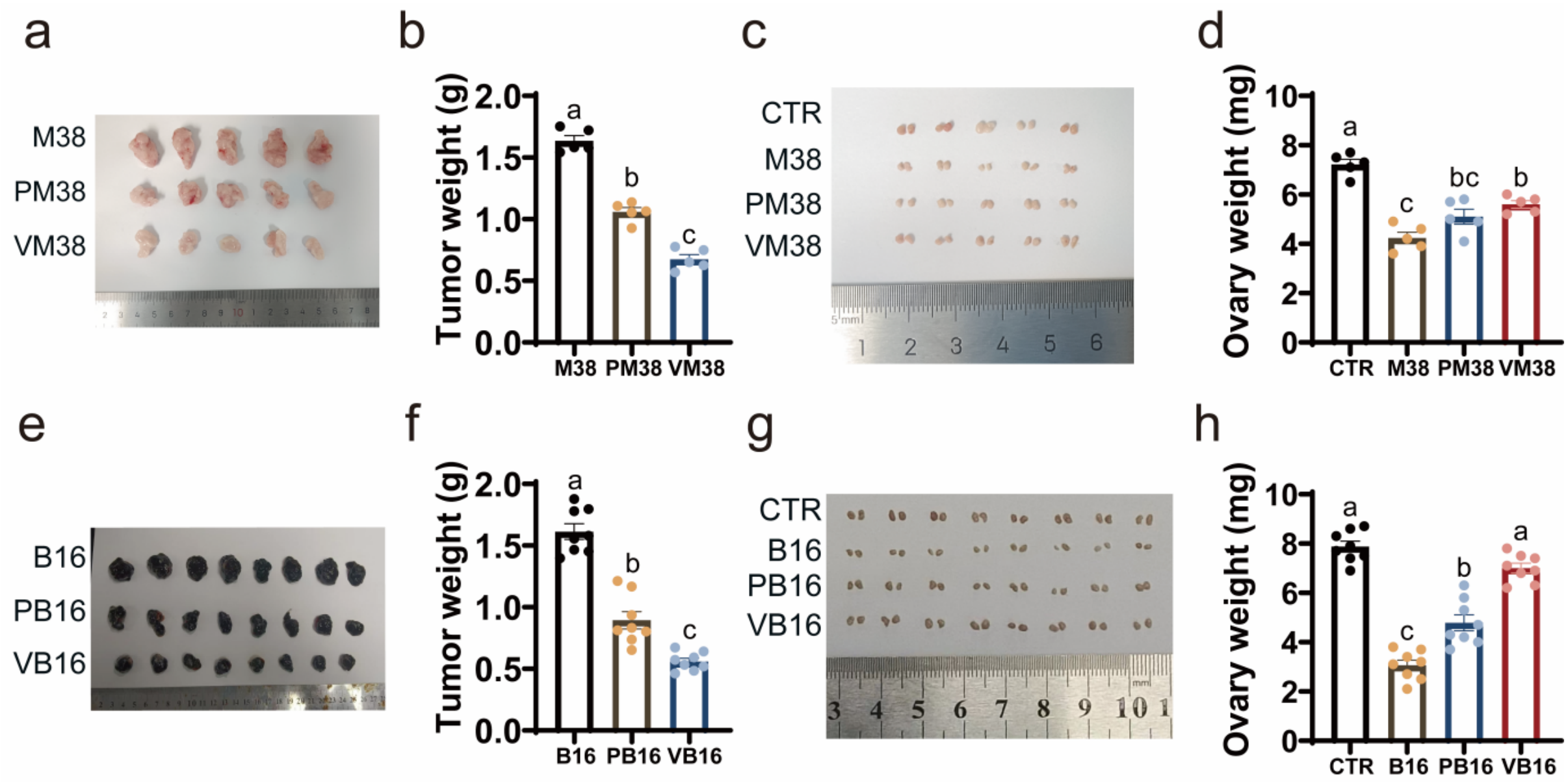

a and **b**. MC38 model. MC38 tumor progression (TP) was successful in M38 group, both WCV treatment in VM38 group and PD-1 treatment in PM38 group significantly reduced tumor weight. N = 5 for all groups. M38 *vs*. PM38, $P < 0.0001$; M38 *vs*. VM38, $P < 0.0001$; VM38 *vs*. PM38, $P < 0.0001$. **c** and **d**. MC38 model. MC38 TP significantly reduced ovarian weight in M38 group, WCV treatment significantly recovered reduced ovarian weight in VM38 group, whereas PD-1 treatment had no obvious impact in PM38 group. N = 5 for all groups. M38 *vs*. CTR, $P < 0.0001$, PM38 *vs*. CTR, $P < 0.0001$, VM38 *vs*. CTR, $P = 0.0008$, M38 *vs*. VM38, $P = 0.0038$. **e** and **f**. B16F10 model. B16F10 TP was successful in B16 group, both WCV treatment in VB16 group and PD-1 treatment in PB16 group significantly reduced tumor weight. N = 8 for all groups. B16 *vs*. VB16, $P < 0.0001$; B16 *vs*. PB16, $P < 0.0001$; VB16 *vs*. PB16, $P = 0.0013$. **g** and **h**. B16F10 model. B16F10 TP significantly reduced ovarian weight in B16 group, WCV treatment significantly recovered reduced ovarian weight in VB16 group, whereas PD-1 treatment had less significant impact in PB16 group. N = 8 for

all groups. B16 *vs*. CTR, $P < 0.0001$; PB16 *vs*. CTR, $P < 0.0001$ B16 *vs*. VB16, $P < 0.0001$; B16 *vs*. PB16, $P = 0.0002$; VB16 vs PB16, $P = < 0.0001$. One-way ANOVA is used for comparisons between multiple (3 or more) groups in the graphs, different letters above the columns indicate significant differences. For all experiments, the number of independent biological repeats (R) was 3.

Supplementary Figure 13. Plasma CXCL10 had the best rescue in WCV-treated group under MC38 or B16F10 progression

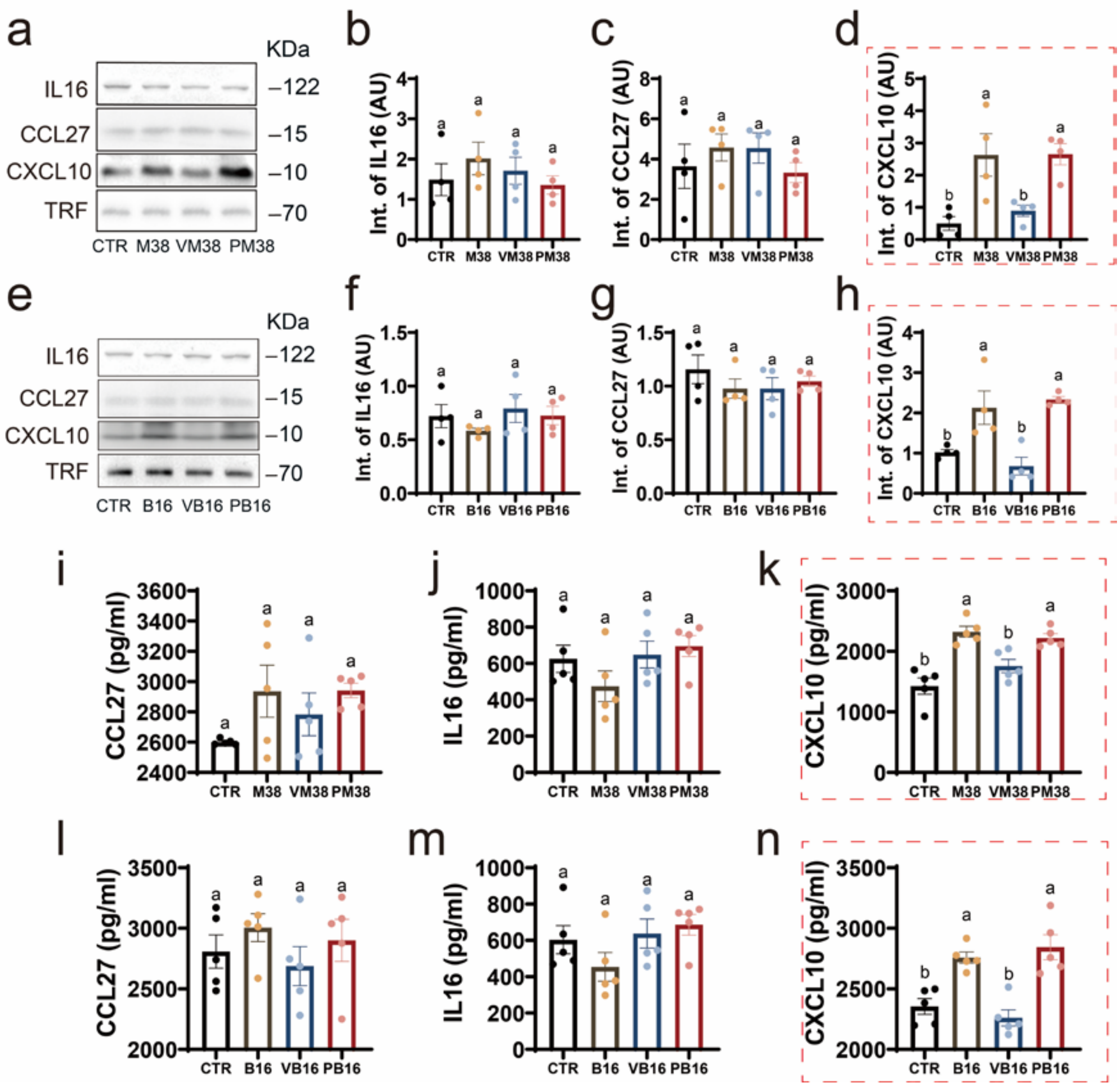

a-d. MC38 model. Western blot and quantification showed that plasma CXCL10 instead of IL16 or CCL27 significantly increased in M38 group, WCV treatment significantly rescued CXCL10 level in VM38 group, whereas PD-1 treatment had no obvious impact in PM38 group. N = 4 for all groups. For CXCL10, M38 *vs*. CTR, P = 0.0113, PM38 *vs*. CTR, P = 0.0105, VM38 *vs*. M38, P = 0.0387, VM38 *vs*. PM38, P = 0.0359. **e-h**. B16F10 model. Western blot and quantification showed that plasma CXCL10 instead of IL16 or CCL27 significantly increased in B16 group, WCV treatment significantly rescued CXCL10 level VB16 group, whereas PD-1 treatment had no obvious impact in PB16 group. N = 4 for all groups. For CXCL10, B16 *vs*. CTR,

P = 0.03, PB16 *vs*. CTR, *P* = 0.0106, VB16 *vs*. B16, *P* = 0.0052, VB16 *vs*. PB16, *P* = 0.0019. **i-k**. MC38 model. Ellisa and quantification showed that CXCL10 instead of IL16 or CCL27 significantly increased in M38 group, WCV treatment significantly rescued CXCL10 level in VM38 group, whereas PD-1 treatment had no obvious impact in PM38 group. N = 5 for all groups. For CXCL10, M38 *vs*. CTR, *P* < 0.0001, PM38 *vs*. CTR, *P* = 0.0003, VM38 *vs*. M38, *P* = 0.0066, VM38 *vs*. PM38, *P* = 0.0274. **l-n**. B16F10 model. Ellisa and quantification showed that CXCL10 instead of IL16 or CCL27 significantly increased in B16 group, WCV treatment significantly rescued CXCL10 level in VB16 group, whereas PD-1 treatment had no obvious impact in PB16 group. N = 5 for all groups. For CXCL10, B16 *vs*. CTR, *P* = 0.0064, PB16 *vs*. CTR, *P* = 0.0012, VB16 *vs*. B16, *P* = 0.001, VB16 *vs*. PB16, *P* = 0.0002. One-way ANOVA is used for comparisons between multiple (3 or more) groups in the graphs, different letters above the columns indicate significant differences. For western blot and ellisa, the number of independent biological repeats (R) was the same as technical repeats (N).

Supplementary Figure 14. CXCL10-IL18R1 and downstream signal pathways are not applicable to ovarian epithelial cancer progression

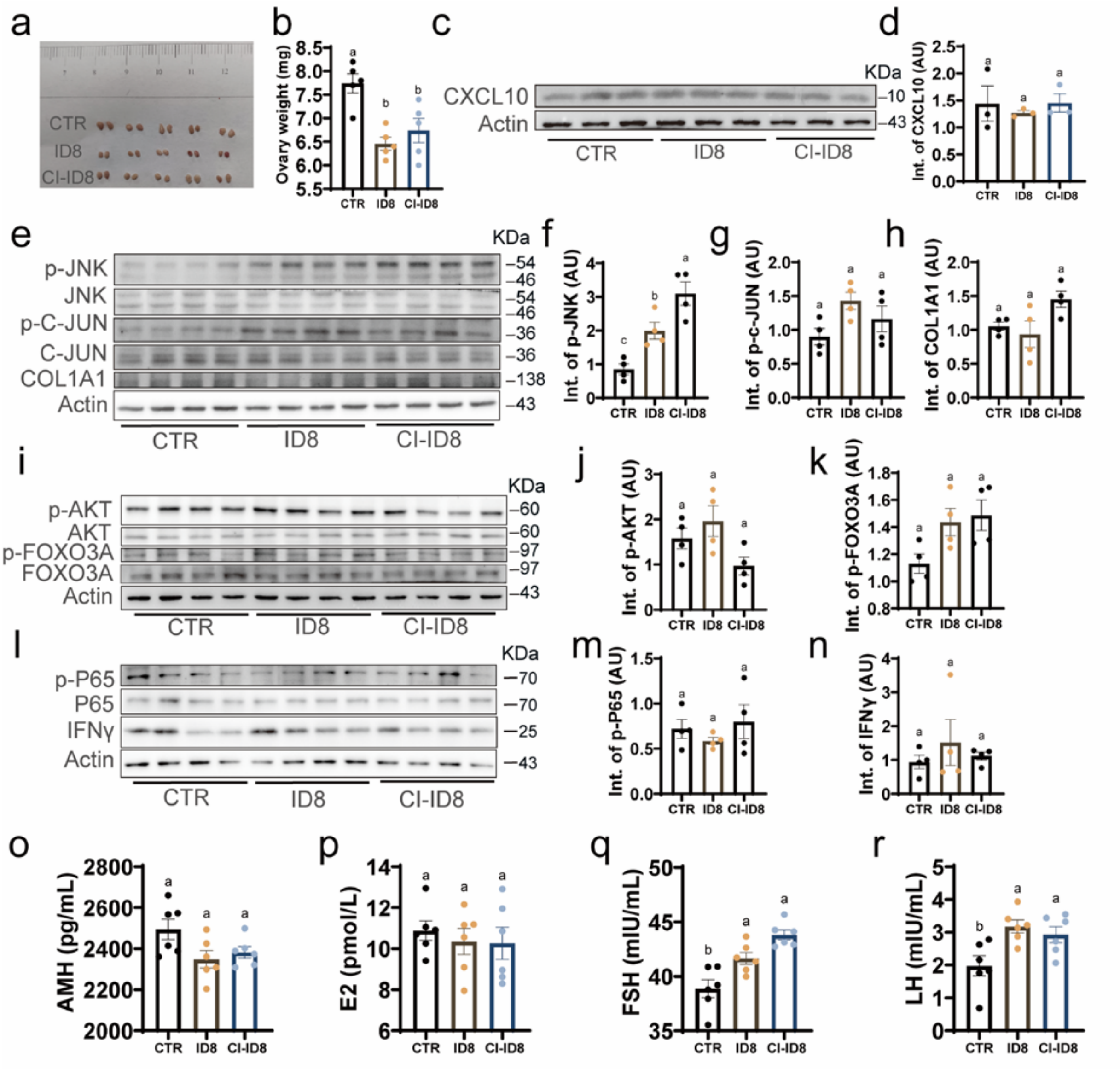

Like Fig. 10, 3 groups were set up: CTR, ID8 (ID8 OECs, ovarian epithelial cells), CI-ID8 (ID8+CIBB). **a and b**. ID8 tumor progression (TP) significantly decreased ovary weight, CIBB failed to rescued ID8 TP-induced ovary weight decrease. N = 5 for all groups. CTR *vs.* ID8, *P* = 0.0023; CTR *vs.* CI-ID8, *P* = 0.0126. **c and d**. Western blot and quantification showed that there were no significant differences between CTR, ID8, and CI-ID8 groups. N = 3 for all groups. **e-h**. Western blot and quantification of

fibrosis-related markers showed that there were no significant differences between ID8 and CI-ID8 groups. N = 4 for all groups. For p-JNK, CTR *vs.* ID8, *P* = 0.0315; CTR *vs.* CI-ID8, *P* = 0.0005; ID8 *vs.* CI-ID8, *P* = 0.0374. **i-k**. Western blot and quantification of PMF activation-related markers showed that there were no significant differences between ID8 and CI-ID8 groups. N = 4 for all groups. **l-n**. Western blot and quantification of inflammation-related markers showed that there were no significant differences between ID8 and CI-ID8 groups. N = 4 for all groups. **o-r**. Ellisa and quantification showed that there were no significant differences between ID8 and CI-ID8 groups for multiple sex hormones. N = 5 for all groups. For FSH, CTR *vs.* ID8, *P* = 0.0171; CTR *vs.* CI-ID8, *P* = 0.0001. For LH, CTR *vs.* ID8, *P* = 0.0115; CTR *vs.* CI-ID8, *P* = 0.045. One-way ANOVA is used for comparisons between multiple (3 or more) groups in the graphs, different letters above the columns indicate significant differences. For western blot and ellisa, the number of independent biological repeats (R) was the same as technical repeats (N); for all other experiments, the number of independent biological repeats (R) was 3.

Supplementary Figure 15. All immunostaining image panels with channel-merged images in all major figures.

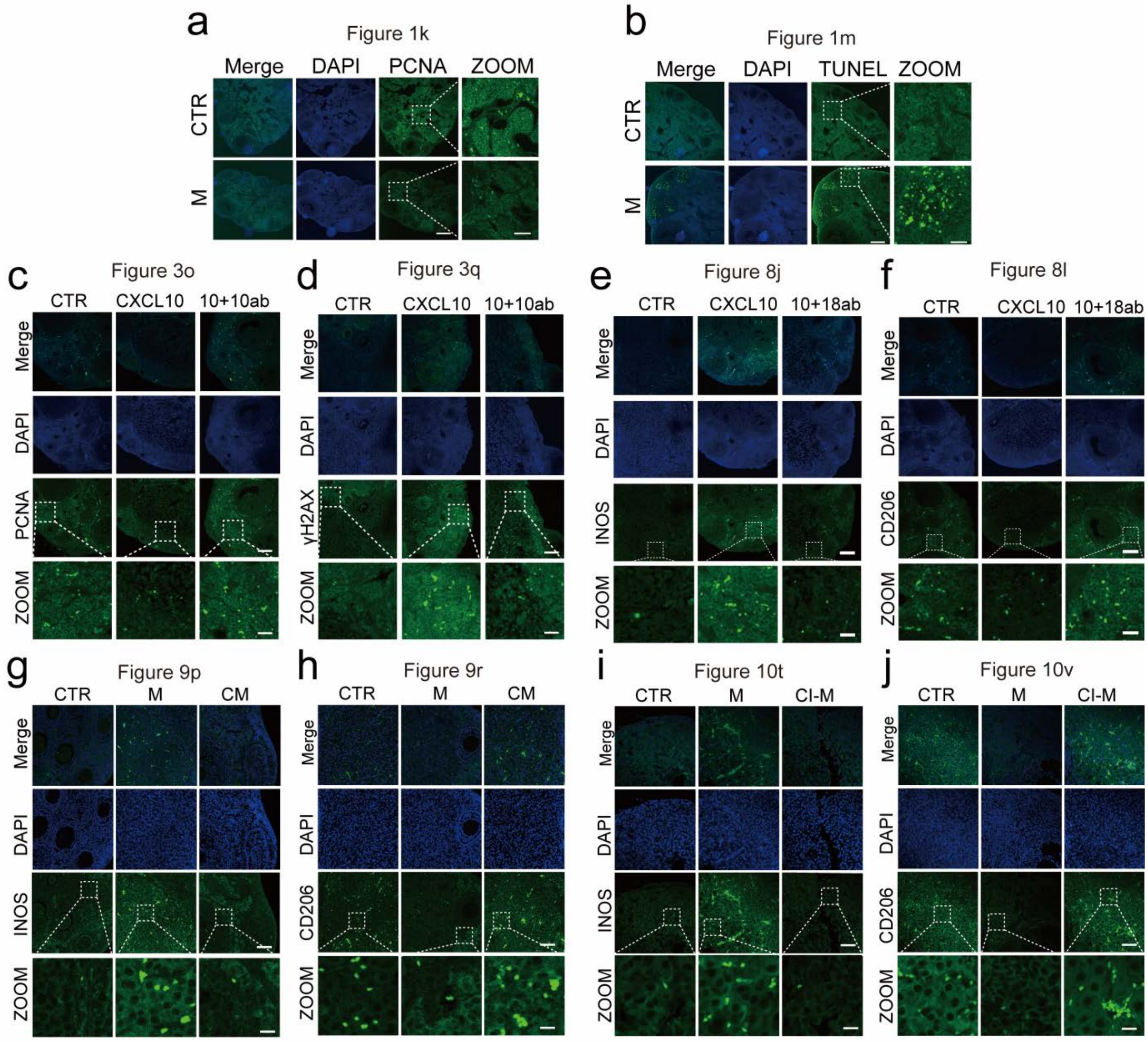

All panels include channel-merged images that correspond to those in major figures. **a** corresponds to Fig. 1k, **b** to Fig. 1m, **c** to Fig. 3o, **d** to Fig. 3q, **e** to Fig. 8j, **f** to Fig. 8l, **g** to Fig. 9p, **h** to Fig. 9r, **i** to Fig. 10t, and **j** to Fig. 10v. Scale bars in zoom image, 25 µm; scale bars in other images, 100 µm. For all immunostaining experiments, the number of independent biological repeats (R) was 3.

Supplementary Tables

Supplementary Table 1.1. Commercial primary antibody information

1^0 or 2^0	Antigen	Host	Clonality	Clone	Company info	Cat #	Lot #
1^0	Strep II-Tag	Mouse	Mono	AMC0521	ABclonal, China	AE066	9200005007
1^0	β-Actin	Mouse	Mono	AC-74	Sigma, USA	A5316	123M4876
1^0	p-H2A.X	Mouse	Mono	D7T2V	CST, USA	80312S	17
1^0	DDDDK-tag	Rabbit	Poly	None	Bioworld, China	AP0007	XS20211117007
1^0	p-c-Jun (Ser73)	Rabbit	Mono	D47G9	CST, USA	3270S	5
1^0	c-Jun	Rabbit	Mono	60A8	CST, USA	9165S	13
1^0	p-P65 (Ser536)	Rabbit	Mono	93H1	CST, USA	3033S	19
1^0	JNK	Rabbit	Poly	None	CST, USA	9252S	18
1^0	p-JNK(Thr183/Tyr185)	Rabbit	Poly	None	CST, USA	9251S	27
1^0	COL1A1	Rabbit	Mono	E8F4L	CST, USA	72026S	5
1^0	P65	Rabbit	Mono	D14E12	CST, USA	8242S	16
1^0	FOXO3A	Rabbit	Mono	D19A7	CST, USA	12829S	6
1^0	IL18R1	Rabbit	Poly	None	ABclonal, China	A2706	0080500101
1^0	p-AKT(Ser473)	Rabbit	Poly	None	BBI, China	D155022	IB22AD0361
1^0	AKT1	Rabbit	Poly	None	BBI, China	D151616	H429AD0352
1^0	p-FOXO3A(Ser253)	Rabbit	Poly	None	BBI, China	D151211	J927AD0355
1^0	AMH	Rabbit	Poly	None	Bioss, China	bs-4687R	AG06126311
1^0	IFN gamma	Rabbit	Poly	None	Bioss, China	bs-0480R	BB03174469
1^0	INOS	Rabbit	Poly	None	Bioss, China	bs-0162R	BB03224889
1^0	CD206	Rabbit	Mono	JF0953	Huabio, USA	ET1702-04	HP0315
1^0	PCNA	Rabbit	Poly	None	BBI, China	D220014	I908AA0007
1^0	CXCR3	Rabbit	Mono	ARC0570	ABclonal, China	A11294	4000000570

1^0 or 2^0, primary or secondary; Host, animals or cells where antibodies are produced; Clonality, monoclonal (mono-) or polyclonal (poly-); Company info., company information; Cat #, Catalog No; Lot #, Lot No; CST, Cell Signaling Technology; BBI, BBI Life Science.

Supplementary Table 1.2. Commercial secondary antibody information

1^0 or 2^0	Antigen	Host	Clonality	Tag	Company info	Cat #
2^0	Mouse IgG	Mouse	Mono	HRP	Jackson, USA	205-032-176
2^0	Rabbit IgG	Goat	Mono	HRP	Beyotime, China	A0208
2^0	Mouse IgG	Donkey	Mono	Cy2	Jackson, USA	715-225-150
2^0	Rabbit IgG	Donkey	Mono	Cy2	Jackson, USA	711-225-152
2^0	Rabbit IgG	Donkey	Poly	TRITC	Jackson, USA	711-025-152

1^0 or 2^0, primary or secondary; Host, animals or cells where antibodies are produced; Clonality, monoclonal (mono-) or polyclonal (poly-); Tag, fluorescence tag or HRP tag; Company info., company information; Cat #, Catalog No; Jackson, Jackson ImmunoResearch.

Supplementary Table 2. Plasmid construction primers for mouse *Cxcl10* and *Il18r1*

Name	Sequence	Length
SalI- IL18R1-F in pFastBac-EGFP-StrepII	ACGCGTCGACTACATCATGAAGAATTAATCTTGAC	1614 bp
NotI- IL18R1-R in pFastBac-EGFP-StrepII	AAGGAAAAAAGCGGCCGCAGGTGCTGAGAGAACAGACC	
EcoRI- Kozac -6xLeu -CXCL10-F in pcDNA3.1&SK(+)	CCGGAATTCATGCTTCTACTTCTACTTCTAAACCCAAGTGCTGCCGTC	297 bp
EcoRV-CXCL10 -R in pcDNA3.1&SK(+)	CCGGATATCAGGAGCCCTTTTAGACCTTTTTT	
HindIII- Kozac -6xLeu -IL18R1-F in pcDNA3.1&SK(+)	CCCAAGCTTGCCACCATGCTTCTACTTCTACTTCTACATCATGAAGAATTAATCTTGAC	2400 bp
XbaI-strepii(IL18R1)-R in pcDNA3.1&SK(+)	CTAGTCTAGATTACTTCTCGAACTGTGGATGACTCC	
EcoRI-CXCL10-F in pGEX 6p1	CCGGAATTCATGAACCCAAGTGCTGCCGTC	297 bp
XhoI-StrepII -CXCL10-R in pGEX6p1	CCGCTCGAGTTACTTCTCGAACTGTGGATGACTCCAAGGAGCCCTTTTAGACCTTTTTT	

Supplementary Table 3. Reverse transcription PCR primers

Name	Sequence	Length
Col1a1-RT-F	CTGGCGGTTCAGGTCCAAT	141 bp
Col1a1-RT-R	CTGGCGGTTCAGGTCCAAT	

Supplementary Table 4. All insert & vector information and related figures

Insert in plasmid	Related figures
CXCL10-TagRFP-Flag in pcDNA3.1(+)	Figure 4C
IL18R1-Egfp-StrepII in pcDNA3.1(+)	Figure 4C
IL18R1-Egfp-StrepII in pFastBac(+)	Figure 4B
CXCL10-strepII in pGEX-6p1	Figure 4A, 4B, 4C and 4F

Supplementary datasets

Supplementary dataset 1

Related to Fig. 3a and 3b. This file includes two sheets. "All_samples-fpkm" includes FPKM values of all identified genes in ovaries from the CTR, M, VM, and PM groups (three repeats per group); "heatmap" includes all average log2 values (from three repeats per group) of 435 DEGs (differentially expressed genes) at the threshold of |log2(M/CTR)| ≥ 1.

Supplementary dataset 2

Related to Fig. 3c. This file includes 33 sheets. "Summary" includes all conc. Values (pg/mL) of 31 plasma DECs (differentially expressed cytokines); "Curves" include standard curves for all 31 cytokines; each of the other 31 sheets includes detailed assays for each plasma cytokine for the CTR, M, VM, and PM groups (five repeats per group).

Supplementary dataset 3

Related to Fig. 5a. This file includes two sheets. “peptides” includes all related values of all identified peptides; “Protein Groups” includes the related info. All the proteins corresponded to the identified peptides.

Supplementary dataset 4

Related to Fig. 4. This file includes 17 sheets. “ECS_C1_DEG” to “ECS_C3_DEG” correspond to DEGs in Cluster 1 to 3 in Fig. 3c; “Fibroblasts_C1_DEG” to “Fibroblasts_C3_DEG” correspond to DEGs in Cluster 1 to 3 in Fig. 3d; “Granulosa Cells_C1_DEG” to “Granulosa Cells_C3_DEG” correspond to DEGs in Cluster 1 to 3 in Fig. 3e; “Theca Cells_C1_DEG” to “Theca Cells_C3_DEG” correspond to DEGs in Cluster 1 to 3 in Fig. 3f; “TandNK_C1_DEG” to “TandNK_C3_DEG” correspond to DEGs in Cluster 1 to 3 in Fig. 3g; “Myeloid Cells_C1_DEG” and “Myeloid Cells_C2_DEG” correspond to DEGs in Cluster 1 and 2 in Fig. 3h. The threshold values of log2(M/CTR), log2(VM/M), log2(PM/VM) are ≥ 0.25 or ≤ -0.25.

Supplementary movies

Supplementary movie 1

Related to Fig. 9a. Model 1 shows the interface contact between CXCL10 and IL18R1. In the magnified interface region, the CXCL10 sequence is highlighted in red, and the IL18R1 sequence is highlighted in green.

Supplementary movie 2

Related to Fig. 9b. Model 2 shows the interface contact between CXCL10 and IL18R1. In the magnified interface region, the CXCL10 sequence is highlighted in purple, and the IL18R1 sequence is highlighted in green.
